# Superdiffusive central limit theorem for a class of driven diffusive systems at the critical dimension

**DOI:** 10.1007/s00440-026-01515-8

**Published:** 2026-07-15

**Authors:** Giuseppe Cannizzaro, Tom Klose, Quentin Moulard

**Affiliations:** 1 University of Warwick, Coventry, UK; 2 University of Oxford, Oxford, UK; 3 Technical University of Vienna, Vienna, Austria

**Keywords:** Stochastic Partial Differential Equations, Critical dimension, Stochastic Burgers equation, Driven diffusive systems, Super-diffusion, Hairer–Quastel universality, 60H17

## Abstract

We study the large-scale behaviour of a class of driven diffusive systems modelled by a Stochastic Partial Differential Equation, the Stochastic Burgers Equation (SBE) with general nonlinearity, at the critical dimension and in infinite volume. Our main result shows that, under a logarithmically superdiffusive space-time scaling, it is given by the same explicit Gaussian Fixed point obtained in [G. Cannizzaro, Q. Moulard, & F. Toninelli, https://arxiv.org/abs/2501.00344, 2025] for the quadratic SBE, but with suitably renormalised coefficients, thereby rigorously justifying and partly correcting the classical Physics derivation of the SBE in [H. van Beijeren, R. Kutner, & H. Spohn, Phys. Rev. Lett., 1986] based on Spohn’s theory of nonlinear fluctuating hydrodynamics. Besides, ours is the first universality-type result for out-of-equilibrium systems and the first extension of [M. Hairer, J. Quastel, Forum of Mathematics, Pi, Vol. 6, 2018, e3], to the critical dimension and beyond weak coupling. The major challenge in our work is the mild growth condition on the nonlinearity which renders even the well-posedness of the microscopic equation non-trivial. Additional key novelties include the derivation of fine estimates on the non-quadratic part of the generator as well as a new approximation for the resolvent associated to the solution of the quadratic SBE.

## Introduction

In the context of out-of-equilibrium statistical mechanics models, the classical theory of nonlinear fluctuating hydrodynamics [49] allows to *heuristically* derive (non-linear) Stochastic Partial Differential Equations (SPDEs) as an effective description for their universal fluctuations around the macroscopic profile. Renowned examples include randomly growing surfaces, which lead to the celebrated KPZ equation [4, 42, 52], lattice gas models, Navier–Stokes dynamics [48], and driven diffusive systems [55].

The goal of the present paper is to rigorously justify (and partly *correct*) this derivation: We show that the large-scale behaviour of a class of driven diffusive systems *at the critical dimension* obeys the same law as that of the corresponding heuristically derived SPDE, the so-called Stochastic Burgers Equation, with *suitably renormalised coefficients*.

### Physical motivation and background

We are interested in a family of random scalar conservation laws taking the form of the non-linear Langevin equation

1.1 $$\begin{aligned} \partial _t u + \nabla \cdot \vec {j}(u) = 0 \, , \quad \vec {j}(u) {\mathop {=}\limits ^{\tiny \text {def}}}- D(u) \nabla u + u \, \vec {w} (u) + \sigma (u) \, \vec {\xi } \, . \end{aligned}$$

where $u=u(t,x)\in \mathbb {R}$ for $(t,x)\in \mathbb {R}_+\times \mathbb {R}^d$, and $D,\,\sigma , \vec {w}$ are arbitrary functions on $\mathbb {R}$, the first two $\mathbb {R}_+$-valued while the latter is $\mathbb {R}^d$-valued. The system in (1.1) was introduced by van Beijeren, Kutner, and Spohn in [55] to model the fluctuations of a conserved scalar quantity *u* in d+1 space-time dimensions. The current density $\vec {j}$ is the result of three contributions: a *diffusive term*, -D(u)∇u, consistent with Fick’s law; an *advective term* $u \,\vec {w}(u)$, which describes the transport of *u* by the flow velocity $\vec {w}(u)$; and a *stochastic term* $\sigma (u) \, \vec {\xi }$, which models random fluctuations via a vector-valued space-time white noise $\vec {\xi } = (\xi _1, \dots , \xi _d)$.

To derive the effective equation governing the behaviour of (1.1), in [55], the authors perform a *perturbative expansion* of the non-linear coefficient $\vec {w}$ around a constant reference density $u_0$ (the *macroscopic profile* alluded to earlier, which is flat as we are implicitly assuming to be at stationarity). This amounts to setting $\bar{u} {\mathop {=}\limits ^{\tiny \text {def}}}u-u_0$ and then Taylor expanding $\vec {w}$ at $u_0$, i.e.

1.2 $$\begin{aligned} \vec {w}(u)= \vec {f}_0 + \vec {f}_1 \, \bar{u} + \vec {f}_2 \, \bar{u}^2 + \dots \,,\quad \text {with }\quad \vec {f}_i=\frac{\vec {w}^{\,(i)}(u_0)}{i!}\,. \end{aligned}$$

In principle, we should also expand *D* and σ but the perturbative corrections beyond the zero-th order term are generally neglected as they are expected to be irrelevant compared to those arising from the nonlinear advection term. We will follow this convention and assume *D* and σ to be constant. For simplicity, we further take $D(u) = \frac{1}{2} \textrm{Id}$ and $\sigma (u) = \textrm{Id}$. From (1.1) and the previous discussion, we obtain an equation for $\bar{u}$ of the form

1.3 $$\begin{aligned} \partial _t \bar{u} =\frac{1}{2} \Delta \bar{u} - \Big [\vec {f}_0\cdot \nabla \bar{u} + \vec {f}_1\cdot \nabla \bar{u}^2 + \vec {f}_2 \, \cdot \nabla \bar{u}^3 + \dots \Big ] - \nabla \cdot \vec {\xi } \, . \end{aligned}$$

Now, the linear term, $\vec {f}_0\cdot \nabla \bar{u}$, can always be removed by performing the Galilean transformation $x \mapsto x + \vec {f}_0 \, t$. Concerning the rest, it is commonly believed (and similarly argued in [55]) that *perturbative terms of degree three and higher have a negligible effect compared to the quadratic one*, a belief which is supported by the following simple scaling argument.

Consider the *diffusive rescaling*, which corresponds to zooming out in space and time as $\bar{u}^\tau (t,x) = \bar{u}^\tau (t,x) = \tau ^{d/4} \, \bar{u}(\tau t, \sqrt{\tau } x)$ for $\tau \gg 1$. Then, equation (1.3) becomes

1.4 $$\begin{aligned} \partial _t \bar{u}^\tau = \frac{1}{2} \Delta \bar{u}^\tau - \Big [\tau ^{1-\frac{d}{2}}\, \vec {f}_1\cdot \nabla (\bar{u}^\tau )^2 + \tau ^{1-d}\vec {f}_2 \, \cdot \nabla (\bar{u}^\tau )^3 + \dots \Big ] - \nabla \cdot \vec {\xi }^\tau \, , \end{aligned}$$

where $\vec {\xi }^\tau$ denotes the appropriately rescaled space-time white noise. Observe that the nonlinear term of degree *m* is now scaled by a factor $\tau ^{1 - (m-1) d/2}$, whose order is decreasing as *m* increases. At large scales, it seems therefore heuristically justified to truncate the expansion in (1.2) beyond the quadratic contribution, leading to the so-called (quadratic) Stochastic Burgers Equation (SBE)

1.5 $$\begin{aligned} \partial _t \bar{u} = \frac{1}{2} \Delta \bar{u} - \,\vec {f_1} \cdot \nabla \bar{u}^2 - \nabla \cdot \vec {\xi } \, . \end{aligned}$$

where the vector $\vec {f_1}$ should be chosen, according to the above heuristic, as in (1.2).

The quadratic SBE has been extensively studied in the Mathematics literature and the characterisation of its (small-scale, only in d=1, and) large-scale behaviour has been at the core of a rich and vibrant area of research. Depending on the spatial dimension *d*, the scaling argument in (1.4) and, in particular, the coefficient of the quadratic term $\tau ^{1-\frac{d}{2}}$ determine whether the equation is *sub-critical*, d=1, *super-critical*, d≥3 or *critical*, d=2. At the sub-critical dimension d=1, the analysis of the small-scales of (1.5) (and of its “primitive” *h*, i.e. $\partial _x h=u$, solving the KPZ equation [42]) has lead to the development of the Theory of Regularity Structures [34, 35], while, at large scales, the SBE was shown to be *polynomially superdiffusive* in [3] and to converge to the KPZ Fixed Point [47, 56]. The super-critical case, d≥3, instead was treated in [11], where diffusive behaviour and asymptotic Gaussianity were determined. At last, the most recent work is [15], which deals with the critical dimension d=2: The solution of SBE is proved to be *superdiffusive*, as in d=1, but only *logarithmically*, and to display, under a logarithmically superdiffusive scaling, *Gaussian fluctuations*, as in d≥3.

The present article is concerned with this latter case and it is the first work which *rigorously formalises* the truncation of (1.3). We consider the system (1.1) at the *critical dimension* d=2 in *infinite volume*, with $D=\frac{1}{2}\sigma =\frac{1}{2} \textrm{Id}$ and $\vec {w}$ unidirectional and smooth, satisfying extremely mild growth assumptions, and show that it converges, under a *logarithmically superdiffusive scaling*, to the same *universal Gaussian limit* as the rescaled solutions to (1.5), but with a choice of the vector $\vec {f}_1$ *different* from that in (1.2), which we explicitly identify (see Theorems 1.3 and 1.4 below for precise statements).

### The model and main results

As mentioned above, our focus is the system (1.1) in dimension d=2. Since we assume $\vec {w}$ to be unidirectional, we can write the advection term as $u \vec {w}(u) = F(u) \, \vec {\mathfrak {w}}$ for some function $F:\mathbb {R}\rightarrow \mathbb {R}$ and constant vector $\vec {\mathfrak {w}} \in \mathbb {S}^1$. Then, the model of interest becomes

1.6 $$\begin{aligned} \partial _t u = \frac{1}{2} \Delta u + \vec {\mathfrak {w}} \cdot \nabla F(u) + \nabla \cdot \vec {\xi } \quad \text {on} \quad \mathbb {R}_+ \times \mathbb {R}^2 \,. \end{aligned}$$

By rotational invariance of the spatial domain, the specific choice of $\vec {\mathfrak {w}}$ is irrelevant; without loss of generality, we fix $\vec {\mathfrak {w}} = \mathfrak {e}_1$, where $(\mathfrak {e}_1,\mathfrak {e}_2)$ denotes the canonical basis of $\mathbb {R}^2$.

As written, the SPDE (1.6) is severely ill-posed due to the singularity of the space-time white noise $\vec {\xi }$. Further, since the dimension d=2 is *critical*, not even the pathwise theories of Regularity Structures [35] or Paracontrolled Calculus [25] apply. In fact, a local solution theory is not even expected to exist. That said, we are interested in *large scales* and thus it is legitimate to start from a microscopically well-defined model. Following the approach of [15], let ρ be a smooth, compactly supported, non-negative, and even function with unit total mass and such that $\Vert \rho \Vert _{L^2(\mathbb {R}^2)} = 1$, and consider the regularised version of (1.6) given by

1.7 $$\begin{aligned} \partial _t u = \frac{1}{2} \Delta u + \mathcal {N}^1_F(u) + \nabla \cdot \vec {\xi }^{ 1} \, \quad \text {on} \quad \mathbb {R}_+ \times \mathbb {R}^2, \end{aligned}$$

where

1.8 $$\begin{aligned} \mathcal {N}_F^1(u) {\mathop {=}\limits ^{\tiny \text {def}}}\rho ^{*2} * \partial _1 F(u) \, , \quad \vec {\xi }^{ 1} {\mathop {=}\limits ^{\tiny \text {def}}}\rho * \vec {\xi } \, . \ \end{aligned}$$

We will require the function $F:\mathbb {R}\rightarrow \mathbb {R}$ to satisfy the following assumption.

#### Assumption 1.1

*(On the non-linearity)* We assume that $F \in \mathcal {C}^\infty (\mathbb {R})$ and that there exists $\kappa \in [0,1/4)$ such that, for every n≥0 and $y \in \mathbb {R}$, it holds that

1.9 $$\begin{aligned} |F^{(n)}(y)| \le \exp (\exp (o(n))) \, \exp (\kappa y^2) \,, \end{aligned}$$

where *o*(*n*) is the usual Landau symbol, i.e. *o*(*n*) is a function of *n* such that *o*(*n*)/*n* converges to 0 as $n\rightarrow \infty$.

Roughly speaking, this assumption encompasses all reasonable smooth functions whose growth is slower than $\exp (x^2/4)$. In particular, it guarantees that the constants $c_1(F)$ and $c_2(F)$, given by

1.10 $$\begin{aligned} c_1(F) {\mathop {=}\limits ^{\tiny \text {def}}}\int _{\mathbb {R}} F'(y) \; \pi _1(\textrm{d}y), \quad c_2(F) {\mathop {=}\limits ^{\tiny \text {def}}}\int _{\mathbb {R}} F''(y) \; \pi _1(\textrm{d}y), \end{aligned}$$

for $\pi _1$ the law of a standard mean-zero real-valued Gaussian random variable, are indeed finite (see Proposition A.5 in Appendix A). We refer the reader to Section 1.3 for a more detailed discussion of Assumption 1.1, interesting examples for which it is satisfied, as well as a more systematic perspective on the constants in (1.10).

Even if the noise (and the nonlinearity) were smoothened in space, it is not at all obvious that (1.7) admits a unique solution. Indeed, the growth condition (1.9) is very weak and we are working in *infinite volume* so that classical analytic tools do not necessarily apply. Our first task in Section 3.1 will then be to identify a good notion of solution (see Definition 3.1) and prove well-posedness of (1.7) (see Proposition 3.2). The results therein are summarised in the next loosely stated proposition.

#### Proposition 1.2

Let *F* be such that Assumption 1.1 holds. Then, (1.7) admits a unique global-in-time solution *u*. Further, *u* is Markov and has $\mathbb {P}^\rho$ as invariant measure, where $\mathbb {P}^\rho$ is the law of $\eta ^1{\mathop {=}\limits ^{\tiny \text {def}}}\eta *\rho$ for η a spatial white noise on $\mathbb {R}^{2}$.

We now turn to the analysis of the large-scale behaviour of *u*. As we expect it to be analogous to that of the solution to (1.7) with *F* quadratic, the relevant scaling should be the one implied by [15, Thm. 1.1]. Since further we need to remove the contribution coming from the linear term (see the discussion after (1.3)), we also place ourselves to a moving frame and consequently define, for a given scale $\tau \ge 1$, the rescaled *u* according to

1.11 $$\begin{aligned} u^\tau (t,x) {\mathop {=}\limits ^{\tiny \text {def}}}\tau ^{1/2} \nu _\tau ^{-1/4} \, u\big (\tau \, t, \tau ^{1/2} R_\tau ^{-1/2} x - \tau t c_1(F) \mathfrak {e}_1\big ) \, \end{aligned}$$

where $c_1(F)$ is as in (1.10) and $R_\tau ,\,\nu _\tau$ satisfy

1.12 $$\begin{aligned} R_\tau {\mathop {=}\limits ^{\tiny \text {def}}}\begin{pmatrix} \nu _\tau &  0 \\ 0 &  1 \end{pmatrix} \, , \qquad \text {for }\quad \nu _\tau {\mathop {=}\limits ^{\tiny \text {def}}}\frac{1}{1 \vee (\log \tau )^{2/3}} \,. \end{aligned}$$

Note that the prefactor in (1.11) is chosen in such a way that the law $\mathbb {P}^\tau$ of the mollified white noise $\eta *\rho _\tau$, with $\rho _\tau (\cdot ) {\mathop {=}\limits ^{\tiny \text {def}}}\tau \nu _\tau ^{-1/2} \, \rho (\tau ^{1/2} R_\tau ^{-1/2} \cdot )$, is a stationary measure for $u^\tau$.

The next is our main result, which is a *superdiffusive central limit theorem* for $u^\tau$. It states that, as $\tau \rightarrow \infty$, the finite dimensional distributions of $u^\tau$ converge to those of $u^{\textrm{eff}}$, the solution to an *anisotropic linear stochastic heat equation* with *explicit renormalised* coefficients that depend solely on $c_2(F)$ as given in (1.10).

#### Theorem 1.3

Let *F* be such that Assumption 1.1 holds and assume $c_2(F) \ne 0$, for $c_2(F)$ as in (1.10). Let $\mathbb {Q}$ be a probability measure, absolutely continuous with respect to the law of a spatial white noise $\mathbb {P}$ and for τ>0, let $(u^\tau (t))_{t \ge 0}$ be as in (1.11) with initial condition $u^\tau (0) = \rho _\tau *u_0$ for $u_0 {\mathop {=}\limits ^{\tiny \text {law}}}\mathbb {Q}$. Then, for any $k \in \mathbb {N}$, times $0 \le t_1 \le t_2 \le \ldots \le t_k$ and test functions $\phi _1, \dots , \phi _k \in \mathcal {S}(\mathbb {R}^2)$, we have

$$\begin{aligned} \big ( \langle {u^\tau (t_1),\phi _1}, \ldots , \langle u^\tau (t_k),\phi _k\rangle \big ) \overset{\textrm{law}}{\Longrightarrow } \big ( \langle {u^{\textrm{eff}}(t_1),\phi _1}, \ldots , \langle u^{\textrm{eff}}(t_k),\phi _k\rangle \big )\qquad \text {as } \tau \rightarrow \infty \text {,} \end{aligned}$$

where $$u^{\textrm{eff}}$$ solves

1.13 $$\begin{aligned} \partial _t u^{\textrm{eff}} = \frac{1}{2} \nabla \cdot D^{\textrm{eff}} \nabla u^{\textrm{eff}} + \nabla \cdot \sqrt{D^{\textrm{eff}}} \vec {\xi } \end{aligned}$$

started at $u^{\textrm{eff}}(0) = u_0$, driven by a 2-dimensional space-time white noise $\mathbf {\xi }= (\xi _1, \xi _2)$, and with diffusion matrix $D^{\textrm{eff}}$ given by

1.14 $$\begin{aligned} \quad D^{\textrm{eff}} {\mathop {=}\limits ^{\tiny \text {def}}}\begin{pmatrix} \displaystyle \frac{3^{2/3}}{2 \pi ^{2/3}} \, |c_2(F)|^{4/3} &  0\\ 0 &  1 \end{pmatrix} \, . \end{aligned}$$

In fact, Theorem 1.3 follows from the more general Theorem 1.4 below, whose statement requires some additional notation. Let $(P^\tau _t)_{t \ge 0}$ (resp. $(P^\textrm{eff}_t)_{t \ge 0}$) be the Markov semigroups of $(u^\tau _t)_{t \ge 0}$ (resp. $(u^\textrm{eff}_t)_{t \ge 0}$). For an initial condition $u_0 \in \mathcal {S}'(\mathbb {R}^2)$, we denote by $\textbf{P}_{u_0}^\tau$ (resp. $\textbf{P}^{\textrm{eff}}_{u_0}$) the law of the process $(u^\tau _t)_{t \ge 0}$ (resp. $(u_t^{\textrm{eff}})_{t \ge 0}$) started at $u_0^\tau = \rho ^\tau * u_0$ (resp. $u_0$), which is well defined for $\mathbb {P}(\textrm{d}u_0)$-almost every initial condition. Let $\textbf{E}_{u_0}^\tau$ (resp. $\textbf{E}^{\textrm{eff}}_{u_0}$) be the corresponding expectation. Finally, for $\theta \in [1,\infty )$, in order to compare elements $G^\tau \in \mathbb {L}^\theta (\mathbb {P}^\tau )$ (on which $P^\tau$ acts) to elements in $\mathbb {L}^\theta (\mathbb {P}^{\textrm{eff}})$ (on which $P^{\textrm{eff}}$ acts), we define the isometric embedding $\iota _\tau : \mathbb {L}^{\theta }(\mathbb {P}^\tau ) \rightarrow \mathbb {L}^\theta (\mathbb {P})$ which maps the functional $\eta ^\tau \mapsto G(\eta ^\tau )$ onto $\eta \mapsto G(\rho ^\tau * \eta )$, see Definition 2.3 below for details. By abuse of notation, we will say that the sequence $(G^\tau )_\tau$ with $G^\tau \in \mathbb {L}^\theta (\mathbb {P}^\tau )$ converges to $G \in \mathbb {L}^\theta (\mathbb {P})$ if $\iota _\tau G^\tau \rightarrow G$ in $\mathbb {L}^\theta (\mathbb {P})$ as $\tau \rightarrow \infty$.

#### Theorem 1.4

In the same setting of Theorem 1.3, let $\theta \in [1,\infty )$.

Then, for any sequence $(G^\tau )_\tau$ which converges to *G* in $\mathbb {L}^\theta (\mathbb {P})$, the sequence $(P^\tau _t G^\tau )_{\tau > 0}$ converges to $P_t^{\textrm{eff}} G$ uniformly in time, that is,

1.15 $$\begin{aligned} \sup _{t \ge 0} \mathbb {E} \left[ \left| \iota _\tau P_t^\tau G^\tau - P_t^{\text {eff}} G \right| ^\theta \right] \longrightarrow 0 \quad \text {as} \quad \tau \rightarrow \infty . \end{aligned}$$

As a consequence, for every k≥1 and continuous functions $f :\mathbb {R}^k \rightarrow \mathbb {R}$ with at most polynomial growth, test functions $\phi _1, \dots , \phi _k \in \mathcal {S}(\mathbb {R}^2)$, and times $0 \le t_1 \le \dots \le t_k$ we have

1.16 $$\begin{aligned} \int \left| \textbf{E}_{u_0}^\tau \left[ f\left( u_{t_1}^\tau (\varphi _1), \dots , u_{t_k}^\tau (\varphi _k)\right) \right] - \textbf{E}_{u_0}^{\text {eff}} \left[ f\left( u_{t_1}^{\text {eff}}(\varphi _1), \dots , u_{t_k}^{\text {eff}}(\varphi _k)\right) \right] \right| ^\theta \mathbb {P}(\textrm{d} u_0) \rightarrow 0, \end{aligned}$$

where, furthermore, the convergence is uniform in $0 \le t_1 \le \dots \le t_k$.

### Discussion of the main results

Theorems 1.3 and 1.4 are the analogues of [15, Thm. 1.3 and 1.4] and, as such, they not only determine the large-scale behaviour of (1.7) but also provide information on the way in which such behaviour is achieved. Indeed, the uniformity in time of (1.15) (due to the mixing properties of the limit $u^\textrm{eff}$) guarantees a control on the law of $u^\tau$ *uniformly across all time scales*. The convergence of the finite-dimensional distributions that (1.16) gives is in $\mathbb {L}^\theta (\mathbb {P})$ for any $\theta \in [1,\infty )$ with respect to the initial condition, so that is *stronger than annealed* (i.e. the convergence in (1.16) for θ=1 but *without* the absolute value) and *stable* (and that of [41, Def. 2.6]).

Furthermore, the above statement realises our main goal as it gives *precise meaning* to the heuristic perturbative argument presented in Section 1.1. Indeed, combining it with the above-mentioned [15, Thm. 1.3], it shows that, as $$\tau \rightarrow \infty $$, $$u^\tau $$ behaves as the quadratic SBE in (1.5) but in which $$\vec {f_1}$$ is *not* that in (1.2) but $$c_2(F)$$ in (1.10) which, in general, is not 0 even if $F^{\prime \prime }\left(0\right)$ is. In other words, higher order terms of the *Taylor* expansion (1.2) *do matter*. Even though such phenomenon has already been observed in the literature (see Section 1.4 for references), let us comment on how it arises and where such constant comes from, as this will also give us the chance to briefly discuss how the proof proceeds.

The stationary solution to (1.7) satisfies $u(t,x) {\mathop {=}\limits ^{\tiny \text {law}}}\pi _1$, for $\pi _1$ as in (1.10), for any given $(t,x) \in \mathbb {R}_+ \times \mathbb {R}^2$. In view of Proposition A.5, Assumption 1.1 ensures that *F* (and all of its derivatives) belong to $L^2(\pi _1)$, so that it admits the Hermite decomposition

1.17 $$\begin{aligned} F = \sum _{m \ge 0} c_m(F) \, H_m\,, \quad c_m(F) {\mathop {=}\limits ^{\tiny \text {def}}}m! \langle F, H_m \rangle _{L^2(\mathcal {N}(0,1))} \, . \end{aligned}$$

where $$(H_m)_m$$ are the classical Hermite polynomials (see (A.1)). Note that this decomposition of *F* is very different from a Taylor expansion: for example, *all even monomials* contribute to the constant $$c_2(F)$$ and so it is not necessarily 0, even if $F^{\prime \prime }\left(0\right)=0$.

Now, let us discuss the contribution of the various terms in the above expansion. W.l.o.g. we can take $c_0(F)=0$ since the derivative in (1.8) would annihilate it anyway. In contrast, the *linear term* (m=1) contributes to $\mathcal {N}^1(u)$ by

1.18 $$\begin{aligned} c_1(F) \, \partial _1 u + c_1(F) \, \partial _1 (\rho ^{*2} - \delta _0) * u \, . \end{aligned}$$

The first summand is a transport term which was removed via the Galilean transformation (see (1.11)). The second is an artefact of our regularisation procedure and, as we shall see later, it is negligible at large scales. Thus, the bulk of the proof of Theorems 1.3 and 1.4 boils down to showing that *all terms of degree* m≥3 *have a negligible effect* on the large-scale dynamics while the *quadratic term* (provided $c_2(F) \ne 0$) gives a dominant superdiffusive contribution.^1^ Making this rigorous is highly non-trivial and a discussion of the difficulties we need to overcome, together with a heuristic as to how we will rigorously do so, can be found in Section 4.1.

In summary, the naive perturbative argument in (1.3) misses a crucial structural fact about the large-scale behaviour of (1.7): what matters in the limit is not the quadratic term in the *Taylor* expansion of the nonlinearity, but the one in its *Hermite expansion*.

#### Remark 1.5

Despite the failure of (1.3) to give the fully correct prediction, let us make the following observation. If one were to work with general $\sigma {\mathop {=}\limits ^{\tiny \text {def}}}\left| \left| [\right| \right| 0]{\rho }_{L^2(\mathbb {R}^2)} > 0$ and decompose $F \in L^2(\pi _\sigma )$ in terms of the rescaled Hermite polynomials $\sigma ^m H_m(\cdot /\sigma )$, then $\sigma ^m H_m(X/\sigma ) \rightarrow X^m/m!$ as $\sigma \rightarrow 0$. In other words, the Hermite and Taylor decompositions agree in the limit when the mollifier ρ becomes flat.

Let us conclude this paragraph by commenting on Assumption 1.1. As will be clearer from the analysis in the following sections, the reason why we require it is that we need the coefficients of the Hermite expansion of *F* in (1.17) to decay sufficiently fast in *m*. That said, in Appendix A (and in particular Lemma A.8), we show that such assumption holds for any analytic function since the growth of their derivatives is at most $n!\ll \exp (\exp (o(n)))$; for example, $F(y)= \sqrt{1 + y^2}$ (see Corollary A.9). We mention the last *F* explicitly as it has played a pivotal role in the original derivation of the KPZ equation from the Eden model [42].

### Comparison to the literature

We conclude the introduction by comparing our result with those present in the literature.

- *Phenomenological derivation of quadratic SBE in dimension* d=1 *at small scales.* In d=1, all the existing results focus on *small scales*, i.e. the goal is to derive the solution of the SBE itself (and not its large-scale behaviour prescribed by the derivative of KPZ Fixed Point) as a suitable scaling limit of (1.6). To do so, one has to tame the exploding factor $\tau ^{1/2}$ which appears in front of the quadratic term in the diffusive rescaling (see (1.4)) and thus the “microscopic” model considered is (1.6) but in *weak coupling*, i.e. with a scale-dependent coefficient $\tau ^{-1/2}$ multiplying *F*. In this setting, the heuristic argument in Section 1.1 was first rigorously formalised in the pioneering work [36], where it is shown that (1.6) with *F* an even polynomial converges to the solution of the SBE with an effective quadratic non-linearity whose strength is the $c_2(F)$ as in (1.10). Several authors have since extended their work to different classes of non-Gaussian driving noises [37, 50], general nonlinearities [23, 38, 39, 45], or both [44], as well as more general smoothing mechanisms [22]. While all of these generalisations present demanding technical challenges that lead to interesting and novel results, in contrast to ours, they are restricted to the *subcritical dimensions* in the *weak coupling regime* and *finite volume*. In particular, equations in this category are within the scope of the pathwise solution theories [25, 35] we mentioned earlier which generally lead to stronger convergence results and typically non-Gaussian limits *without* the additional assumption that the equation is started from stationarity. Another class of results in d=1 is based on the notion of energy solutions in [27, 29], which, as for us, requires a rather explicit knowledge of the invariant measure already at the microscopic level. In particular, Gubinelli and Perkowski in [28] have given another proof of [36] in the case of a general Lipschitz *F*, and their work was later generalised to non-stationary initial conditions and infinite volume in [57, 58]. Roughly speaking, their argument relies on the fact that the time-averaged non-linearity, in probabilistic square norm, gains *strictly more than one power* of the time-increment which they then leverage to get tightness (and ultimately convergence). It is easy to convince oneself that it is precisely at the critical dimension that this strategy breaks down, as one would only gain *exactly one power* by the same argument. On the technical side, one ramification of this observation is that, in contrast to [28], we have to control the action of the generator (and of the resolvent) much more thoroughly, see Section 4.1 for a more precise discussion of this and further aspects at the heart of our strategy.
- *SPDEs and other models at the critical dimension.* Turning to SPDEs and other statistical mechanics models at the critical dimension, recent years have witnessed a flurry of activity most of which though (a) work in *weak coupling scaling* (compared to d=1, the factor that multiplies the non-linearity, the noise, or the initial condition is $1/\sqrt{\log \tau }$), and (b) feature a *polynomial* (mostly quadratic) non-linearity. This is the case of the KPZ equation [5, 17, 33], the AKPZ equation [7, 9], the SBE [11], the fractional KPZ equation [31], the (Landau–Lifshits–)Navier–Stokes equation [13, 43], (self-)interacting diffusions [10, 60]^2^, the Allen–Cahn equation [32], and the multiplicative linear Stochastic Heat Equation in d=2 [2, 16, 18, 24, 53]. Notable results that avoid (a) (but satisfy (b)) include the so-called DCGFF model of [1, 12, 14, 54] and the derivation of scaling exponents for ASEP [59], AKPZ [8], SBE [20], while those that avoid (b) (but satisfy (a)) include the non-linear stochastic heat equation [19, 21, 51] and the Allen–Cahn equation [6]. Compared to the above results, ours is the first in *strong coupling* with a *generic non-linearity* satisfying a weak growth condition, but we still require to be close to the (explicit) invariant measure already at the microscopic scale and impose the non-linearity to be anisotropic (i.e. the flow velocity $\vec {w}$ in (1.1) is unidirectional).

### Organisation of the article

The rest of the article is organised as follows. In Section 2, we recall basic tools from Wiener space analysis and introduce the notations we will use throughout the article. In Section 3.1, we introduce a suitable notion of solution to the microscopic equation (1.7) for a fixed value of τ>0, show that such a solution exists and is unique, and prove that it defines a stationary, skew-reversible Markov process whose invariant measure is a smoothened spatial white noise. In Section 3.2, we then obtain properties of the Markov semigroup associated with (1.7), identify a sufficiently rich set in the domain of its generator, and explicitly describe the action of the latter on the corresponding Fock spaces. The key challenge here is the fact that the nonlinearity has components in every chaos: we overcome it by a polynomial approximation argument for the antisymmetric part of the generator and present a novel, direct proof, based on the Itô trick, that the resolvent is continuous between suitable spaces; we believe the latter to be of independent interest. Section 4 contains the proofs of our main results which, on a heuristic level, we anticipate in Section 4.1. Therein, we present a high-level overview of the major challenges, outline the ideas to overcome them, and describe a roadmap for their implementation. Section 4.2 comprises novel estimates on the non-quadratic part of the generator while Section 4.3 presents a new ansatz for the resolvent of the quadratic SBE which is truncated both in chaos and in Fourier. Finally, we collect all previous results in Section 4.4 to obtain the desired resolvent convergence and then show how it implies Theorems 1.3 and 1.4. At last, Appendix A collects some facts about the connection between the regularity of a function and the decay of its Hermite coefficients, complemented by some representative examples of functions satisfying Assumption 1.1. Appendix B contains some technical proofs concerning the antisymmetric part of the generator in the polynomial case.

## Preliminaries

Let us collect in this section a few preliminary tools and notations which are mainly taken from [15, Section 1.3].

### Notations, Function spaces and Wiener space analysis

Let $\mathfrak {e}_1$ and $\mathfrak {e}_2$ be the canonical basis vectors of $\mathbb {R}^2$ and |·| be the usual Euclidean norm. For *I* a finite subset of $\mathbb {N}$ of cardinality |*I*| and a set of |*I*| vectors $\{p_i\in \mathbb {R}^2:i\in I\}$, we set $p_I\in \mathbb {R}^{2|I|}$ to be the vector $(p_i)_{i\in I}$ and, given a 2×2 matrix *M*, $M p_{I}{\mathop {=}\limits ^{\tiny \text {def}}}(M p_i)_{i\in I}$. We write (for $e \in \mathbb {R}^2$)

2.1 $$\begin{aligned} p_{[I]} = \sum _{i\in I} p_i \, , \quad |p_{I}| {\mathop {=}\limits ^{\tiny \text {def}}}\Big (\sum _{i\in I}|p_i|^2\Big )^\frac{1}{2}\,,\qquad |e \cdot p_{I}| {\mathop {=}\limits ^{\tiny \text {def}}}\Big (\sum _{i\in I} |e \cdot p_i|^2\Big )^\frac{1}{2} \,. \end{aligned}$$

For τ>0, let the matrix $R_\tau$ and $\nu _\tau > 0$ be respectively given according to

2.2 $$\begin{aligned} R_\tau {\mathop {=}\limits ^{\tiny \text {def}}}\begin{pmatrix} \textrm{dis}\nu _\tau &  0\\ 0 &  1 \end{pmatrix}\qquad \text {and}\qquad \nu _\tau {\mathop {=}\limits ^{\tiny \text {def}}}\frac{1}{\big (1 \vee \log \tau \big )^{2/3}} \, , \end{aligned}$$

and note that it holds

2.3 $$\begin{aligned} |\sqrt{R_\tau } p_{I}|^2 = \nu _\tau |\mathfrak {e}_1 \cdot p_{I}|^2 + |\mathfrak {e}_2 \cdot p_{I}|^2 \, . \end{aligned}$$

In the specific case of $I=\{1,\dots ,n\}$, we write I=1:n so that, in particular, $p_{1:n}=(p_1,\dots ,p_n)$.

We denote by $\mathcal {S}(\mathbb {R}^d)$ the Schwartz space of smooth functions whose derivatives decay faster than any polynomial and by $\mathcal {S}'(\mathbb {R}^d)$ its dual. The Fourier transform of $\phi \in \mathcal {S}(\mathbb {R}^d)$ is

$$\begin{aligned} \mathcal {F}(\phi )(p) = \hat{\phi }(p) {\mathop {=}\limits ^{\tiny \text {def}}}\frac{1}{(2 \pi )^{d/2}} \int _{\mathbb {R}^d} \phi (x) \, e^{- \iota \, p \cdot x} \, \textrm{d}x \, , \end{aligned}$$

while, for $f\in \mathcal {S}'(\mathbb {R}^d)$, it is given via duality.

Let $\rho \in \mathcal {S}(\mathbb {R}^2)$ be a given mollifier, i.e. ρ is smooth, compactly supported, even-symmetric, of mass 1 and of $L^2(\mathbb {R}^2)$-norm 1. For τ>0, we set

2.4 $$\begin{aligned} \rho _\tau (\cdot )\! {\mathop {=}\limits ^{\tiny \text {def}}}\! \tau \nu _\tau ^{-1/2} \, \rho (\tau ^{1/2} R_\tau ^{-1/2} \cdot )\,,\quad \text {and}\quad \Theta _\tau (\cdot ) {\mathop {=}\limits ^{\tiny \text {def}}}(2 \pi \hat{\rho }_\tau (\cdot ))^2=(2 \pi \hat{\rho }(\tau ^{-1/2} R_\tau ^{1/2} \cdot ))^2\,, \end{aligned}$$

and, for $n\in \mathbb {N}$, we further define the measure $\Xi _n^\tau$ on $\mathbb {R}^{2n}$ according to

2.5 $$\begin{aligned} \Xi _n^\tau (\textrm{d}p_{1:n}) {\mathop {=}\limits ^{\tiny \text {def}}}\prod _{i=1}^n \Theta _\tau (p_i) \, \textrm{d}p_{1:n} \, ,\qquad p_{1:n}\in \mathbb {R}^{2n} \end{aligned}$$

with the convention that, for $\tau = \infty$, $\Theta _\tau \equiv 1$ and $\Xi _n=\Xi _n^\infty$ is the Lebesgue measure.

For τ>0 and $\gamma \in \mathbb {R}$, let $H^\gamma _\tau (\mathbb {R}^{2n})$ be the τ-dependent (anisotropic) Sobolev space with respect to $\Xi _n^\tau$, i.e. $H^\gamma _\tau (\mathbb {R}^{2n})$ is the completion of $\mathcal {S}(\mathbb {R}^{2n})$ under the norm

2.6 $$\begin{aligned} \Vert f\Vert _{H^\gamma _\tau (\mathbb {R}^{2n})}^2 {\mathop {=}\limits ^{\tiny \text {def}}}\int \Big (1 + \frac{1}{2} \, |\sqrt{R_\tau } p_{1:n}|^2\Big )^\gamma \, |\hat{f}(p_{1:n})|^2 \, \Xi _n^\tau (\textrm{d}p_{1:n}) \, . \end{aligned}$$

For γ=0, $H^0_\tau (\mathbb {R}^{2n})=L_\tau ^2(\mathbb {R}^{2n}) {\mathop {=}\limits ^{\tiny \text {def}}}L^2(\mathbb {R}^{2n}, \Xi _n^\tau )$, with scalar product $\langle \cdot , \cdot \rangle _{L^2_\tau (\mathbb {R}^{2n})}$.

Finally, we will write a≲b if there exists a constant C>0 independent of any quantity relevant for the result, such that a≤Cb and a≍b if a≲b and b≲a. If we want to highlight the dependence of the constant *C* on a specific quantity *Q*, we write instead $\lesssim _Q$.

### Gaussian white noise and Fock spaces

Let η be a space white noise on $\mathbb {R}^2$, and, for $\tau \ge 0$, set $\eta ^\tau {\mathop {=}\limits ^{\tiny \text {def}}}\rho _\tau *\eta$ (with $\eta ^\infty \equiv \eta$). Then, $\eta ^\tau$ (whose law is denoted by $\mathbb {P}^\tau$) is a centred Gaussian field with covariance

$$\begin{aligned} \mathbb {E}^\tau [\eta ^\tau (\phi )\eta ^\tau (\psi )] =\langle \phi ,\psi \rangle _{L_\tau ^2(\mathbb {R}^2)} = \int _{\mathbb {R}^{2}}\hat{\phi }(p)\,\overline{\hat{\psi }(p)}\,\Xi _1^\tau (\textrm{d}p)\,,\qquad \phi ,\psi \in L^2_\tau (\mathbb {R}^2)\,. \end{aligned}$$

Cylinder random variables $\phi \in \mathbb {L}^\theta (\mathbb {P}^\tau )$ are random variables of the form $\phi =f(\eta ^\tau (h_1),\dots ,\eta ^\tau (h_n))$, with $f:\mathbb {R}^n\rightarrow \mathbb {R}$ smooth and growing at most polynomially at infinity, and $h_1,\dots ,h_n\in \mathcal {S}(\mathbb {R}^2)$. The set of cylinder random variables is dense in $\mathbb {L}^\theta (\mathbb {P}^\tau )$, for $\theta <\infty$.

By [46, Theorem 1.1.1], $\mathbb {L}^2(\mathbb {P}^\tau )= \overline{\bigoplus _{n \ge 0} \mathscr {H}_n^\tau }$, where $\mathscr {H}_n^\tau$ is the *n*-*th homogeneous Wiener chaos*, i.e. the closure in $\mathbb {L}^2(\mathbb {P}^\tau )$ of $\textrm{Span}\big \{H_n(\eta ^\tau (h)) \, | \, h \in \mathcal {S}(\mathbb {R}^2), \, \Vert h\Vert _{L_\tau ^2(\mathbb {R}^2)} = 1\big \}$ and $H_n$ is the *n*-th Hermite polynomial (see (A.1)). Let $I^\tau$ be the canonical isometry onto $\mathbb {L}^2(\mathbb {P}^\tau )$ (see [46, Theorem 1.1.2]) obtained as the unique extension of the map that, for every n≥0, assigns $h^{\otimes n}$ to $n! \, H_n(\eta ^\tau (h)) \in \mathcal {H}^\tau _n$ for $h\in \mathcal {S}(\mathbb {R}^2)$ with $L^2_\tau (\mathbb {R}^2)$-norm equal to 1. $I^\tau$ is defined on the Fock space , where  contains functions which are symmetric with respect to permutations of their variables. We endow  with the norm2.7for , and define, for $\gamma \in \mathbb {R}$,  as the Sobolev space whose norm is the same as that in (2.7) but with $\Vert \cdot \Vert _{H^\gamma _\tau (\mathbb {R}^{2n})}^2$ replacing $\Vert \cdot \Vert _{L^2_\tau (\mathbb {R}^{2n})}^2$. Recall that by [46, Theorem 1.1.2], for any $F\in \mathbb {L}^2(\mathbb {P}^\tau )$ there exists  such that $F=\sum _n I_n(f_n)$ and $\mathbb {E}^\tau [F^2]=\Vert f\Vert _\tau ^2$, where $I^\tau _n$ is the restriction of $I^\tau$ to  (which is itself an isometry).

Thanks to the isometry $I^\tau$, we will abuse notation and identify operators acting on $\mathbb {L}^2(\mathbb {P}^\tau )$ with the corresponding operator acting on . An important notion is that of *diagonal* operator (see [15, Def. 1.5]).

#### Definition 2.1

*(Def. 1.5 in* [15]) An operator $\mathcal {D}$ on  is said to be *diagonal* if there exists a family of measurable real-valued kernels $\textbf{d} = (\textbf{d}_n)_{n \ge 0}$ such that for all n≥0 and , it holds $\mathcal {F}{\mathcal {D}\phi } (p_{1:n}) = \textbf{d}_n(p_{1:n}) \, \widehat{\phi }(p_{1:n})$ for (Lebesgue-almost-) every $p_{1:n} \in \mathbb {R}^{2n}$. If $\mathcal {D}^{(1)}$ and $\mathcal {D}^{(2)}$ are diagonal, we write $\mathcal {D}^{(1)} \le \mathcal {D}^{(2)}$ if their kernels satisfy $\textbf{d}^{(1)} \le \textbf{d}^{(2)}$ and say that $\mathcal {D}^{(1)}$ is positive if $\mathcal {D}^{(1)} \ge 0$. Given a diagonal operator $\mathcal {D}$ with kernel $\textbf{d}=(\textbf{d}_n)_{n \ge 0}$ and a measurable scalar function *f*, we define $f(\mathcal {D})$ to be the diagonal operator whose kernel is $f(\textbf{d})=(f \circ \textbf{d}_n)_{n \ge 0}$.

#### Definition 2.2

We define the number operator $\mathcal {N}$ to be the diagonal operator acting on  such that $\Vert \psi _n\Vert _\tau$ decays faster than any polynomial in *n*, as $\mathcal {N}\psi _n{\mathop {=}\limits ^{\tiny \text {def}}}n\psi _n$.

Let us recall the operators $\iota ^\tau$ and $j^\tau$ that appeared in [15] (see [15, Lemma 1.7]).

#### Definition 2.3

Let us define $\iota ^\tau :\mathbb {L}^\theta (\mathbb {P}^\tau ) \rightarrow \mathbb {L}^\theta (\mathbb {P})$, $\theta \in [1,\infty )$, to be the operator that maps the functional $\eta ^\tau \mapsto F(\eta ^\tau )$ to the functional $\eta \mapsto F(\rho _\tau * \eta )$. This is a bijective isometry which, for θ=2, translates to a bijective isometry from  to . We denote  its inverse.

We will also need the product rule for the Wiener-Itô integrals. For  and , it reads

2.8 $$\begin{aligned} I_{n_1}^\tau (f) I_{n_2}^\tau (g) = \sum _{k=0}^{n_1 \wedge n_2} k! \left( {\begin{array}{c}n_1\\ k\end{array}}\right) \left( {\begin{array}{c}n_2\\ k\end{array}}\right) I_{n_1+n_2-2k}\big (\textrm{Sym} (f \otimes _k g)\big ) \, , \end{aligned}$$

where $\textrm{Sym}$ is the symmetrization operator and

2.9 $$\begin{aligned} \mathcal {F}(f \otimes _k g) \, (p_{1:n_1-k}, q_{1:n_2-k}) {\mathop {=}\limits ^{\tiny \text {def}}}\int \Xi ^\tau _k(\textrm{d}r_{1:k}) \hat{f}(p_{1:n_1-k}, r_{1:k}) \, \hat{g}(q_{1:n_2-k}, -r_{1:k}) \, . \end{aligned}$$

## Analysis of the microscopic equation

Let us begin by collecting some basic results concerning our microscopic model, i.e. equation (1.7). Compared to the analysis carried out in Section 2 and Appendix A of [15] the arguments below are more delicate due to the very mild assumptions on the function *F* but, as the setting is similar, we will borrow the same notations (that will be recalled along the way). We will first prove well-posedness of the SPDE (1.7) and subsequently detail the derivation of the generator (and semigroup) of its solution.

Throughout the rest of the paper, let us fix a probability space $(\mathcal {O},\mathcal {F},\textbf{P})$ supporting a couple of independent spatial white noise η on $\mathbb {R}^2$ and 2-dimensional space-time white noise $\vec {\xi }=(\xi _1,\xi _2)$ on $\mathbb {R}_+ \times \mathbb {R}^2$, whose laws are respectively denoted by $\mathbb {P}$ and *P*, and $\textbf{P}{\mathop {=}\limits ^{\tiny \text {def}}}\mathbb {P}\otimes P$. Let $(\mathcal {F}_t)_{t \ge 0}$ be the filtration in which $\mathcal {F}_t$ is the (completed) σ-algebra generated by η and . Let $\rho \in \mathcal {S}(\mathbb {R}^2)$ be the mollifier introduced above and, for τ>0, $\rho _\tau$ be as in (2.4). Let $\eta ^\tau {\mathop {=}\limits ^{\tiny \text {def}}}\eta *\rho _\tau$ and $\vec {\xi }^{\tau } {\mathop {=}\limits ^{\tiny \text {def}}}\vec {\xi }*\rho _\tau =(\xi _1*\rho _\tau ,\xi _2*\rho _\tau )$ the rescaled white noises, whose joint law is $\textbf{P}^\tau {\mathop {=}\limits ^{\tiny \text {def}}}\mathbb {P}^\tau \otimes P^\tau$.

### Well-posedness for the microscopic equation

Our first goal is to prove that the rescaled version of the regularised equation (1.7) admits a unique solution globally in space and time, and that such solution is Markovian and stationary. Throughout this subsection the scale parameter τ>0 will be fixed and bounds and estimates below will (badly) depend on it. By scaling (and shifting) the solution *u* of (1.7) as in (1.11), we obtain

3.1 $$\begin{aligned} \partial _t u^\tau = \frac{1}{2} \, \nabla \cdot R_\tau \, \nabla u^\tau + \mathcal {N}_F^\tau [u^\tau ] + \nabla \cdot \sqrt{R_\tau } \, \vec {\xi }^\tau \, , \end{aligned}$$

where $R_\tau$ is the 2×2 matrix in (1.12) and, for $F:\mathbb {R}\rightarrow \mathbb {R}$, the rescaled nonlinearity $\mathcal {N}_F^\tau$ is

3.2 $$\begin{aligned} \mathcal {N}_F^\tau [u^\tau ] {\mathop {=}\limits ^{\tiny \text {def}}}\tau \nu _\tau ^{1/4} \, \partial _1 \rho _\tau ^{*2} * F(\tau ^{-1/2} \nu _\tau ^{1/4} u^\tau ) - c_1(F) \, \tau ^{1/2} \nu _\tau ^{1/2} \, \partial _1 u^\tau \, \end{aligned}$$

for $c_1(F)$ as in (1.10).

Even though, *apriori*, the function *F* should be subject to Assumption 1.1, for the sake of this section it will be sufficient to require that $F\in \mathcal {C}^1(\mathbb {R})$ and that there exists $\kappa \in (0,1/4)$ (which will be fixed throughout) such that both conditions

3.3 $$\begin{aligned}  &   \Vert F\Vert _{\exp } \overset{\text {def}}{=} \sup _{x \in \mathbb {R}} \left( |F(x)| \vee |F'(x)| \right) e^{-\kappa x^2} < +\infty , \nonumber \\  &   \lim _{|x| \rightarrow \infty } \left( |F(x)| \vee |F'(x)| \right) e^{-\kappa x^2} = 0. \end{aligned}$$

hold^3^. The spaces in which we will look for a solution have to take into account the growth of *F*. Thus, for β>0, we define $\mathcal {X}_\beta$ to be the metric space completion of smooth compactly supported functions on $\mathbb {R}^2$ under the distance

3.4 $$\begin{aligned} d_\beta (f,g) {\mathop {=}\limits ^{\tiny \text {def}}}\sum _{k \ge 0} \min \Big \{2^{-k}, \sup _{x \in \mathbb {R}^2} |f(x)-g(x)| \exp (- 2^{-k} \langle x \rangle ^\beta )\Big \} \, , \end{aligned}$$

where, for $x\in \mathbb {R}^2$, we set $\langle x\rangle {\mathop {=}\limits ^{\tiny \text {def}}}(1+|x|^2)^{1/2}$. Let us point out that if $\beta>\beta '>0$ then $\mathcal {X}_\beta \subset \mathcal {X}_{\beta '}$ and that the choice of the distance in (3.4) guarantees that if $f\in \mathcal {X}_\beta$ then *for all* C>0

3.5 $$\begin{aligned} \sup _{x\in \mathbb {R}^2}e^{-C\langle x\rangle ^\beta }|f(x)|<\infty \,. \end{aligned}$$

The notion of solution we will be adopting is the following.

#### Definition 3.1

Let τ>0, $\kappa \in (0,1/4)$ and $F\in \mathcal {C}^1(\mathbb {R})$ be such that (3.3) holds. We say that $u^\tau$ is a mild solution of (3.1) driven by $(\eta ^\tau ,\vec {\xi }^{\, \tau })$ if

1. $$u^\tau $$ is $$(\mathcal {F}_t)_{t \ge 0}$$-adapted,
2. $\textbf{P}$-almost surely, $u^\tau \in C(\mathbb {R}_+, \mathcal {X}_1)$ and, for $\alpha \in (4\kappa ,1)$,
  3.6 $$\begin{aligned} t\mapsto \mathfrak {E}_\alpha (u^\tau _t){\mathop {=}\limits ^{\tiny \text {def}}}\sup _{x \in \mathbb {R}^2} \frac{e^{\kappa |u^\tau _t(x)|^2}}{\langle x \rangle ^{\alpha }} \in L^1_{\textrm{loc}}(\mathbb {R}_+), \end{aligned}$$
3. for every t≥0
  3.7 $$\begin{aligned} u^\tau _t = K^\tau _t\eta ^\tau + \int _0^t K^\tau _{t-s} \mathcal {N}_F^\tau [u_s] \, \textrm{d}s + \int _0^t K^\tau _{t-s} \, \nabla \cdot \sqrt{R_\tau } \vec {\xi }^{\, \tau }(\textrm{d}s) \, , \end{aligned}$$
  where, for t≥0, $K^\tau _t$ is the heat semigroup associated with $\tfrac{1}{2} \nabla \cdot R_\tau \, \nabla u^\tau$.

Let us briefly comment on Definition 3.1. The first and the last condition are natural as they require $u^\tau$ to be a stochastic process adapted to the filtration induced by the noise that indeed solves (3.1) (in its mild formulation). As we will soon see, the second instead is needed to obtain uniqueness. Even though (3.6) might appear quite strong, in our setting it can be shown to hold since the $u^\tau$ we will construct is stationary. This means that the $\textbf{P}$-almost sure boundedness of the $L^1$-norm (in time) of $\mathfrak {E}_\alpha (u^\tau )$ follows by the boundedness of the expectation of $\mathfrak {E}_\alpha (\eta ^\tau )$ (and this is the reason why we require $L^1$, see Lemma 3.5), which in turn can be easily deduced as $\eta ^\tau$ is a smooth Gaussian process with finite range of dependence.

We are now ready to state the main result of this subsection.

#### Proposition 3.2

Let τ>0 be fixed, $\kappa \in (0,1/4)$ and $F\in \mathcal {C}^1(\mathbb {R})$ be such that (3.3) holds. Then, there exists a unique mild solution $u^\tau$ of (3.1) driven by $(\eta ^\tau ,\vec {\xi }^{\, \tau })$ according to Definition 3.1. Furthermore, for any $h\in \mathcal {C}_c^\infty (\mathbb {R}^2)$, we have that, $\textbf{P}$-almost surely, for every t≥0 it holds

3.8 $$\begin{aligned} u^\tau _t(h) = \eta ^1(h) + \frac{1}{2}\int _0^t u_s(\nabla \cdot R_\tau \, \nabla h) \, \textrm{d}s + \int _0^t \mathcal {N}^\tau _F[u_s](h) \, \textrm{d}s + \int _0^t \vec {\xi }^{\, \tau }(\textrm{d}s, \sqrt{R_\tau }\nabla h) \, . \end{aligned}$$

Besides, $u^\tau$ is a Markov process which is stationary, i.e. for every t≥0, $u^\tau _t {\mathop {=}\limits ^{\tiny \text {law}}}\eta ^\tau$, and skew-reversible, in the sense that for any T>0, under $\textbf{P}$, the process $(u^\tau _{T-t})_{t \in [0,T]}$ has the same law as the process $(\tilde{u}^\tau _t)_{t \in [0,T]}$, for $\tilde{u}^\tau$ the mild solution of (3.1) driven by $(\tilde{\eta }^\tau ,\vec {\tilde{\xi }}^{\, \tau }){\mathop {=}\limits ^{\tiny \text {law}}}(\eta ^\tau ,\vec {\xi }^{\, \tau })$ but with *F* replaced by -F.

#### Remark 3.3

The reason why the weak formulation of (3.1) in (3.8) is stated for compactly supported smooth functions ϕ and not for $\phi \in \mathcal {S}(\mathbb {R}^2)$ is that, in space, the solution *u* only belongs to $\mathcal {X}_1$ (so that in particular it might grow at infinity exponentially fast) and thus the pairing $u_\cdot (\phi )$ might not be well-defined.

As usual, the proof of the previous proposition consists of showing that a solution to (3.1) (1) exists, (2) is unique and (3) enjoys all the stated properties. For (1), we will consider the sequence $(u^{\tau ,M})_{M\in \mathbb {N}}$, where $u^{\tau ,M}$ solves (3.1) on a torus of side-length *M* (and then extended periodically to $\mathbb {R}^2$) whose nonlinearity is not *F* but a polynomial $P_M$ with $P_M$ converging to *F* in a suitable sense (see Lemma A.6). We will show that such sequence is Cauchy and that its limit satisfies (3.7) (and (3.8)), thus obtaining (1) and, as a byproduct, (3), since, by the arguments presented in [15, Appendix A], it is not hard to prove that each of the $u^{\tau ,M}$ is Markov, stationary and skew-reversible (and this is the reason why we chose such approximation) and these properties are carried through in the limit.

The next lemma allows us to simultaneously prove that the sequence $(u^{\tau ,M})_{M\in \mathbb {N}}$ is Cauchy and that the solution to (3.1) is unique, as it shows that the solution map is Lipschitz continuous with respect to *both* the driving noise *and* the nonlinearity, thus allowing us to carry out the outlined strategies for (1) and (2) (and (3)) at once.

#### Lemma 3.4

Let τ>0 be fixed, T>0, $F, G\in \mathcal {C}^1(\mathbb {R})$ satisfying (3.3), $\beta \in (0,1]$ and $X^\tau , Y^\tau \in C([0,T], \mathcal {X}_\beta )$. Assume there exist $u^\tau , v^\tau \in C([0,T], \mathcal {X}_\beta )$ such that, for $\alpha \in (0,\beta )$, $\mathfrak {E}_\alpha (u^\tau ), \mathfrak {E}_\alpha (v^\tau )\in L^1(0,T)$ and, for every t∈[0,T], they respectively satisfy

3.9 $$\begin{aligned} u^\tau _t = X^\tau _t + \int _0^t K^\tau _{t-s} \mathcal {N}^\tau _F[u^\tau _s] \, \textrm{d}s \, , \qquad v^\tau _t = Y^\tau _t + \int _0^t K^\tau _{t-s}\mathcal {N}^\tau _G[v^\tau _s] \, \textrm{d}s \, . \end{aligned}$$

Then there exists a constant $C=C(\tau , T,\alpha ,\beta )>0$ that depends continuously on $\Vert F\Vert _{\exp },$ $\Vert \mathfrak {E}_\alpha (u^\tau )\Vert _{L^1(0,T)}$ and $\Vert \mathfrak {E}_\alpha (v^\tau )\Vert _{L^1(0,T)}$ (where $\Vert \cdot \Vert _{\exp }$ and $\mathfrak {E}_\alpha (\cdot )$ are respectively defined in (3.3) and (3.6)), such that for every t∈[0,T] and $x \in \mathbb {R}^2$

3.10 $$\begin{aligned} |u^\tau _t(x) - v^\tau _t(x)| \le C e^{C \langle x \rangle ^\beta } \, \Big (\Vert F - G\Vert _{\exp } + \sup _{(t,x) \in [0,T]\times \mathbb {R}^2} e^{- \langle x \rangle ^\beta }\, |X^\tau _t(x) - Y^\tau _t(x)| \Big )\, . \end{aligned}$$

#### Proof

Throughout the proof, we will denote by *C* a constant as in the above statement, but whose value may increase from line to line and, since the specific value of τ is irrelevant, we will take it to be 1 and omit the corresponding index (e.g. we write *u*, *X* for $u^\tau , X^\tau$ etc.). Recall that, for τ=1, we have $\nu _\tau = 1$ and thus $R_\tau = \operatorname {Id}_{2}$ and $K_t \equiv K_t^1$ is the classical heat semigroup w.r.t. $\frac{1}{2}\Delta$.

Let us define $w {\mathop {=}\limits ^{\tiny \text {def}}}u - v$. Then, from (3.9) and the definition of $\mathcal {N}^1$ in (3.2), we see that for every t∈[0,T], *w* equals

3.11 $$\begin{aligned} w_t&= \int _0^t K_{t-s} \big (\mathcal {N}_F^1[u_s]-\mathcal {N}_F^1[v_s]\big ) \, \textrm{d}s + \int _0^t K_{t-s} \, \big (\mathcal {N}_F^1[v_s]-\mathcal {N}_G^1[v_s]\big ) \, \textrm{d}s +\big (X_t - Y_t\big )\nonumber \\&=:\mathrm {(I)}_t+\mathrm {(II)}_t+\mathrm {(III)}_t \end{aligned}$$

and we will separately estimate each of the terms above. Let us begin with $\mathrm {(III)}$, which is the easiest as, for every $x\in \mathbb {R}^2$, we immediately have

3.12 $$\begin{aligned} |\mathrm {(III)}_t(x)| \le \, e^{\langle x\rangle ^\beta } \sup _{(t,y) \in [0,T]\times \mathbb {R}^2} e^{- \langle y \rangle ^\beta } \, |X_t(y) - Y_t(y)|\, . \end{aligned}$$

The bound on $\mathrm {(II)}$ is an immediate consequence of two bounds. The first follows by the definition of $\Vert \cdot \Vert _{\exp }$ in (3.3) and of $\mathfrak {E}_\alpha$ in (3.6), and reads

$$\begin{aligned} |(F-G)(v_s(x))| \le \Vert F - G\Vert _{\exp } \exp (\kappa v_s(x)^2) \le \Vert F - G\Vert _{\exp } \mathfrak {E}_\alpha (v_s) \, \langle x \rangle ^{\alpha } \, , \end{aligned}$$

while the second is a simple convolutional bound (which can be obtained by using e.g. Feynman-Kac formula) that takes the form

3.13 $$\begin{aligned} K_{r} \, \big [|\partial _1 \rho ^{*2}| * \langle \cdot \rangle ^{\alpha }\big ](x) \le C \langle x \rangle ^{\alpha } \,, \end{aligned}$$

for some constant C>0, uniformly over r∈[0,T]. Putting them together, we obtain

3.14 $$\begin{aligned} |\mathrm {(II)}_t(x)| \le C \Vert \mathfrak {E}_\alpha (v)\Vert _{L^1(0,T)} \, \Vert F - G\Vert _{\exp } \, \langle x \rangle ^\alpha \le C \, \Vert F - G\Vert _{\exp } \, e^{\langle x\rangle ^\beta } \,, \end{aligned}$$

where the constant *C* in the last bound clearly depends (linearly) on $\Vert \mathfrak {E}_\alpha (v)\Vert _{L^1(0,T)}$.

We now turn to $\mathrm {(I)}$, which is the most delicate. We first notice that

3.15 $$\begin{aligned}  &   |F(u_s(x)) - F(v_s(x))| \le \Vert F\Vert _{\exp } \big (e^{\kappa u_s(x)^2} + e^{\kappa v_s(x)^2}\big ) \, |w_s(x)| \nonumber \\  &   \le \Vert F\Vert _{\exp } (\mathfrak {E}_\alpha (u_s) + \mathfrak {E}_\alpha (v_s)) \, \langle x \rangle ^\alpha \, |w_s(x)| \, . \end{aligned}$$

Now, since both *u* and *v* satisfy (3.5) by assumption, so does *w*, which means that morally we should have $|w_s(x)|\lesssim e^{A\langle x\rangle ^\beta +B}$ for some A,B>0. If we plug this into the previous bound and back to the definition of *I* in (3.11), we will need to estimate

3.16 $$\begin{aligned} K_{r} \, \big [|\partial _1 \rho ^{*2}| *\big (\langle \cdot \rangle ^\alpha e^{A \langle \cdot \rangle ^\beta + B}\big )\big ](x) \le e^{C'(1+A^2)} \, \langle x \rangle ^\alpha e^{A \langle x \rangle ^\beta + B} \end{aligned}$$

where, for some $C^{\prime }=C^{\prime }\left(T,\beta \right)>0$, the bound is uniform over r∈[0,T] and can be derived similarly to (3.13). At this point though, we see a problem. Indeed, while we are assuming *w* to grow as $$e^{A\langle x\rangle ^\beta +B}$$, at the r.h.s. there is an extra polynomial factor in *x* so that in principle it grows *faster* than what is expected and it is unclear how to close the bound. What we have neglected is the integral in time, and to exploit it we will choose *A* and *B* to be *time-dependent*. More specifically, for $$A, B:[0,T]\rightarrow \mathbb {R}$$ to be chosen afterwards and s∈[0,T], set

3.17 $$\begin{aligned} W_s{\mathop {=}\limits ^{\tiny \text {def}}}\sup _{x \in \mathbb {R}^2} |w_s(x)| e^{-A_s \langle x \rangle ^\beta - B_s}\qquad \text {and}\qquad W_t^*{\mathop {=}\limits ^{\tiny \text {def}}}\sup _{s \in [0,t]} W_s\,. \end{aligned}$$

With these definitions at hand together with (3.15) and (3.16), we can estimate $\mathrm {(I)}$ as

3.18 $$\begin{aligned} |\mathrm {(I)}_t(x)|&\le \int _0^t \textrm{d}s \, W_s \, \Vert F\Vert _{\exp } (\mathfrak {E}_\alpha (u_s) + \mathfrak {E}_\alpha (v_s)) \, e^{C'(1+A_s^2)}\langle x \rangle ^\alpha e^{A_s \langle x \rangle ^\beta + B_s} \,\nonumber \\&\le W_t^* \int _0^t \textrm{d}s \, \Vert F\Vert _{\exp } (\mathfrak {E}_\alpha (u_s) + \mathfrak {E}_\alpha (v_s)) \, \big [\langle x \rangle ^\beta + e^{C'(1+A_s^2)}\big ] e^{A_s \langle x \rangle ^\beta + B_s}. \end{aligned}$$

At this point, we want to choose *A* and *B* in such a way that we can control the time integral by a small constant (say 1/2) times the exponential of $A_t \langle x \rangle ^\beta + B_t$ so that, once we get back to (3.11), we can reabsorb this term at the l.h.s. . A convenient choice is to set, for every t∈[0,T],

$$\begin{aligned} A_t&= 1 + 2 \int _0^t \Vert F\Vert _{\exp } (\mathfrak {E}_\alpha (u_s) + \mathfrak {E}_\alpha (v_s)) \textrm{d}s \,,\\ B_t&= 2 \int _0^t \Vert F\Vert _{\exp } (\mathfrak {E}_\alpha (u_s) + \mathfrak {E}_\alpha (v_s)) e^{C' (1+A_s^2)} \textrm{d}s \end{aligned}$$

as these are well-defined, non-negative, non-decreasing and bounded by some constant *C* depending (continuously) on $T,\alpha ,\beta , \Vert F\Vert _{\exp },$ $\Vert \mathfrak {E}_\alpha (u)\Vert _{L^1(0,T)}$ and $\Vert \mathfrak {E}_\alpha (v)\Vert _{L^1(0,T)}$, and, even more importantly, allow us to take care of the polynomial factor. Indeed, from (3.18) we can bound $\mathrm {(I)}$ as

3.19 $$\begin{aligned} |\mathrm {(I)}_t(x)| \le W_t^* \int _0^t \textrm{d}s \, \frac{1}{2} \big (\dot{A}_s \langle x \rangle ^\beta + \dot{B}_s\big ) e^{A_s \langle x \rangle ^\beta + B_s} \le \frac{1}{2} \, W_t^*\,e^{A_t \langle x \rangle ^\beta + B_t} \, , \end{aligned}$$

which concludes the bound on $\mathrm {(I)}$.

We now turn to (3.11) and apply (3.12), (3.14) and (3.19), so that we deduce

$$\begin{aligned} W_t \le \frac{1}{2} W_t^* + C \Big (\Vert F-G\Vert _{\exp } + \sup _{(t,y) \in [0,T]\times \mathbb {R}^2} e^{- \langle y \rangle ^\beta } \, |X_t(y) - Y_t(y)|\Big ) \, , \end{aligned}$$

where we further used that, since $A_0=1$ and *A*, *B* are non-decreasing and non-negative, $\langle x \rangle ^\beta \le A_s \langle x \rangle ^\beta + B_s$ for every $(s,x)\in [0,T]\times \mathbb {R}^2$. Note, in particular, that it is precisely this estimate which takes care of the exponential factor $e^{\langle x\rangle ^\beta }$ in (3.12) as, by definition, $W_t$ contains multiplication by $e^{-A_t \langle x \rangle ^\beta - B_t}$. By assumption, both *u* and *v* (and thus *w*) satisfy (3.5), and *A* and *B* are bounded, which means that, for every t∈[0,T], $W^*_t<\infty$. As a consequence, we can take the $W_t^*/2$ to the l.h.s. , and deduce that for every t∈[0,T]

$$\begin{aligned} |w_t(x)|&\le 2 C \,e^{A_t \langle x \rangle ^\beta + B_t} \Big (\Vert F-G\Vert _{\exp } + \sup _{(t,y) \in [0,T]\times \mathbb {R}^2} e^{- \langle y \rangle ^\beta } \, |X_t(y) - Y_t(y)|\Big )\\&\le 2 C \,e^{A_T \langle x \rangle ^\beta + B_T} \Big (\Vert F-G\Vert _{\exp } + \sup _{(t,y) \in [0,T]\times \mathbb {R}^2} e^{- \langle y \rangle ^\beta } \, |X_t(y) - Y_t(y)|\Big ) \end{aligned}$$

where in the last step we exploited once again the fact that *A* and *B* are non-decreasing. By rebranding the constant *C* according to the prefactor at the r.h.s., (3.10) follows at once. □

In order to make use of the previous lemma, we need to ensure that, for the approximation outlined after the statement of Proposition 3.2, the constant *C* for which (3.10) holds (that depends continuously $\Vert \mathfrak {E}_\alpha (u^\tau )\Vert _{L^1(0,T)}$ and, for us, will be random) is tight and the other terms at the r.h.s. do indeed go to 0. Our approximation consists of solutions to (3.1) with a polynomial nonlinearity and driven by periodic noise and initial condition. These were introduced in [15, eq. (A.7)] and we now recall their definition. Let $(\eta ^\tau , \vec {\xi }^\tau )$ be as above. For M>0, let $(\eta ^\tau _M, \vec {\xi }^\tau _M)$, whose law is denoted $\mathbb {P}^\tau _M \otimes P^\tau _M$, be given by3.20and if M=∞ then we set $(\eta ^\tau _\infty , \vec {\xi }^\tau _\infty ) {\mathop {=}\limits ^{\tiny \text {def}}}(\eta ^\tau , \vec {\xi }^\tau )$.

In the next lemma we show that, for any stationary process $t\mapsto u^\tau _t(\cdot )$, the quantity $\Vert \mathfrak {E}_\alpha (u^\tau )\Vert _{L^1(0,T)}$ is indeed tight.

#### Lemma 3.5

Let τ>0, $\alpha > 4 \kappa$ and, for M>0, define $\eta ^\tau _M$ according to (3.20). Then,

3.21 $$\begin{aligned} \sup _{M \in (0, +\infty ]} \mathbb {E}[\mathfrak {E}_\alpha (\eta ^\tau _M)] < +\infty \, . \end{aligned}$$

As a consequence, for every T>0 and δ>0, there exists a constant $C = C(\tau , \alpha , T, \delta ) > 0$ such that any $\mathbb {P}_M^\tau$-stationary locally continuous process $u^\tau$ (that is for every t>0, $u^\tau _t {\mathop {=}\limits ^{\tiny \text {law}}}\eta ^\tau _M$) it holds

3.22 $$\begin{aligned} \textbf{P}\big (\Vert \mathfrak {E}_\alpha (u^\tau )\Vert _{L^1(0,T)} \ge C\big ) \le \delta \, . \end{aligned}$$

#### Proof

By a straightforward scaling argument using (1.11) and since we do keep track of the dependence in τ, we take τ=1. First, (3.22) follows by (3.21), after a straightforward application of Markov’s inequality and Fubini’s theorem. For (3.21), note that we always have $\mathfrak {E}_\alpha (\eta ^1_M) \le \mathfrak {E}_\alpha (\eta ^1)$, as can be directly checked using that for any $x \in [-M, M]^2$ and $k \in 2 M \mathbb {Z}^2$, $\langle x+k \rangle \ge \langle x \rangle$. Thus it is sufficient to prove that $\mathbb {E}[\mathfrak {E}_\alpha (\eta ^1)]$ is finite.

To prove that the latter, recall that $\eta ^1$ is a Gaussian field such that, for all $y\in \mathbb {R}^2$, $\eta ^1(y)$ is a centred Gaussian random variable of variance 1. Define, for $k\in \mathbb {Z}^2$, the random variable $S_k = \sup _{x \in [-1/2,1/2]^2} |\eta ^1(k+x)|$ and note that, by stationarity, the $S_k$’s are identically distributed and each of them is almost surely finite. By Borell–TIS inequality, we thus deduce that there exists C>0 such that for every A>0 it holds

$$\begin{aligned} \mathbb {P}(S_k > A) \le C \exp (- A^2/2) \, . \end{aligned}$$

By union bound and using that there exists $C^{\prime }>0$ such that $$\langle x+k \rangle \ge (C')^{-1} \langle k \rangle $$ for every $$k \in \mathbb {Z}^2$$ and $$x \in [-1/2,1/2]^2$$, we deduce that for every λ>0 it holds

$$\begin{aligned} \mathbb {P}[\mathfrak {E}_\alpha (\eta ^1)> \lambda ] \le \sum _{k \in \mathbb {Z}^2} \mathbb {P}\Big (e^{\kappa S_k^2}/\langle k \rangle ^\alpha > C' \lambda \Big )\lesssim \sum _{k \in \mathbb {Z}^2} \frac{1}{\lambda ^{\frac{1}{2 \kappa }} \langle k \rangle ^{\frac{\alpha }{2 \kappa }}} \lesssim \frac{1}{\lambda ^{\frac{1}{2 \kappa }}} \, , \end{aligned}$$

where, in the last bound, we used that $\alpha > 4 \kappa$. Since κ<1/4 (actually, here we only need κ<1/2), we get $\mathbb {E}[\mathfrak {E}_\alpha (\eta ^1)] < +\infty$, which concludes the proof. □

We are now ready to complete the proof of Proposition 3.2.

#### Proof of Proposition 3.2

Uniqueness follows immediately from Lemma 3.4 applied with F=G and $X^\tau _t = Y^\tau _t = K^\tau _t \eta ^\tau + \int _0^t K^\tau _{t-s} \nabla \cdot \sqrt{R_\tau } \, \vec {\xi }^\tau (\textrm{d}s)$.

To prove existence and the stated properties, let $(P_M)_M$ be a sequence of polynomials such that $\Vert F-P_M\Vert _{\exp }\rightarrow 0$ as $M\rightarrow \infty$, whose existence is guaranteed by Lemma A.6, and for any *M*, let $u^{\tau , M}$ be the unique (periodic) mild solution of (3.1) driven by $(\eta ^\tau _M, \vec {\xi }^\tau _M)$, defined according to (3.20), and with nonlinearity $P_M$. That such solution exists, is unique, Markov, $\mathbb {P}_M^1$-stationary, skew-symmetric and satisfies the weak formulation is shown in [15, Lemma A.4] for the case of $P_M$ quadratic, but the arguments therein work *mutatis mutandis* for a generic polynomial of any given degree. In particular, (3.7) holds and $\textbf{P}$-almost surely, for every t≥0, we have

$$\begin{aligned} u^{\tau ,M}_t = X^{\tau ,M}_t + \int _0^t K^\tau _{t-s} \mathcal {N}^\tau _{P_M}[u^{\tau ,M}_s] \, \textrm{d}s \, , \end{aligned}$$

where $X^M$ is defined by

$$\begin{aligned} X^M_t {\mathop {=}\limits ^{\tiny \text {def}}}K^\tau _t \eta ^\tau _M + \int _0^t K^\tau _{t-s} \nabla \cdot \sqrt{R_\tau } \, \vec {\xi }^\tau (\textrm{d}s) \, . \end{aligned}$$

We want to show that the sequence $(u^{\tau ,M})_M$ is Cauchy. Let $4 \kappa< \alpha< \beta < 1$ and T>0. Lemma 3.4, applied to $u = u^{\tau ,M}$ and $v = u^{\tau ,M'}$, gives that $\textbf{P}$-almost surely for every $(t,x)\in [0,T]\times \mathbb {R}^2$, we have

$$\begin{aligned}&|u_t^{\tau ,M}(x) - u_t^{\tau ,M'}(x)| \\&\le \ C_M e^{C_M \langle x \rangle ^\beta } \, \Big (\Vert P_M - P_{M'}\Vert _{\exp } + \sup _{(t,x) \in [0,T]\times \mathbb {R}^2} e^{- \langle x \rangle ^\beta }\, |X^{\tau ,M}_t(x) - X^{\tau ,M'}_t(x)| \Big ) \, , \end{aligned}$$

where we recall that $C_M$ is a random positive constant that depends continuously on $\Vert P_M\Vert _{\exp }$, $\Vert \mathfrak {E}(u^{\tau ,M})\Vert _{L^1(0,T)},\Vert \mathfrak {E}(u^{\tau ,M'})\Vert _{L^1(0,T)}$ (the other quantities on which $C_M$ depends are independent of *M*). Since the sequence $(\Vert P_M\Vert _{\exp })_M$ converges and the family $(\Vert \mathfrak {E}_\alpha (u^M)\Vert _{L^1(0,T)})_{M \ge 1}$ is tight in view of Lemma 3.5 (recall that for every *M*, $u^M$ is $\mathbb {P}_M^1$-stationary), we conclude that the sequence $(C_M)_M$ is tight. Further, the two summands in parenthesis converge to 0 as $M,M'\rightarrow \infty$: the first deterministically by Lemma A.6, the second in probability thanks to [15, Lemma A.2] (this would be even true replacing the $\exp (- \langle x\rangle ^\beta )$ by any $\langle x\rangle ^{- \varepsilon }$, ε>0).

Therefore, as β<1, we deduce that the sequence of processes $(u^{\tau ,M})$ is Cauchy in the complete metric space consisting of processes living in $\mathcal {C}([0,T], \mathcal {X}_1)$, equipped with the distance $d(u,v) = \mathbb {E}[1 \wedge \sup _{t \in [0,T]} d_1(u_t,v_t)]$. Thus, up to extracting a subsequence, we conclude that there exists a process $u^\tau \in \mathcal {C}([0,T], \mathcal {X}_1)$ which is the $\textbf{P}$-almost surely limit (along the subsequence) of $(u^{\tau ,M})_{M \ge 1}$ in $\mathcal {C}([0,T], \mathcal {X}_1)$, and, via a diagonal argument, one can prove that $u^\tau \in \mathcal {C}(\mathbb {R}_+, \mathcal {X}_1)$ and that the convergence takes place in the same space.

It is then immediate to pass through the limit all the properties of $u^{\tau ,M}$. In particular, the limit is $(\mathcal {F}_t)_{t \ge 0}$-adapted; $\mathbb {P}^1$-stationary, and thus $t\mapsto \mathfrak {E}(u^\tau _t)\in L^1_{\textrm{loc}}(\mathbb {R}_+)$; it satisfies (3.7), and therefore it is the necessarily unique mild solution to (3.1) driven by $(\eta ^\tau , \vec {\xi }^\tau )$ according to Definition 3.1; the weak formulation (3.8) holds; it is skew-symmetric. As for the Markov property, it is an immediate consequence of the existence, adaptedness and uniqueness of solutions of (3.1). □

### The semigroup and the generator of the generalised SBE

In view of Proposition 3.2, for every τ>0, (3.1) admits a unique solution $u^\tau$ which is a stationary Markov process. Thus, its semigroup, $P^\tau$, given by

3.23 $$\begin{aligned} P_t^\tau \phi {\mathop {=}\limits ^{\tiny \text {def}}}\textbf{E}^\tau \big [\phi (u^\tau _t) \, \big | \, \mathcal {F}_0\big ] \, , \end{aligned}$$

is well-defined. Let us state a preliminary lemma providing basic properties of $P^\tau$ and the generator $\mathcal {L}^\tau$ associated to it.

#### Lemma 3.6

Let $\theta \in [1,\infty )$. Then, $(P^\tau _t)_{t\ge 0}$ in (3.23) defines a contractive strongly continuous semigroup on $\mathbb {L}^\theta (\mathbb {P}^\tau )$ such that, for every $\phi \in \mathbb {L}^\theta (\mathbb {P}^\tau )$,

3.24 $$\begin{aligned} \Vert P_t^\tau \phi \Vert _{\mathbb {L}^\theta (\mathbb {P}^\tau )} \le \Vert \phi \Vert _{\mathbb {L}^\theta (\mathbb {P}^\tau )} \end{aligned}$$

holds uniformly in τ and also for $\theta =\infty$. In particular, the generator $\mathcal {L}^\tau :\textrm{Dom}(\mathcal {L}^\tau ) \subset \mathbb {L}^\theta (\mathbb {P}^\tau ) \rightarrow \mathbb {L}^\theta (\mathbb {P}^\tau )$ associated to $P^\tau$ is closed and, for every μ>0, its resolvent $(\mu -\mathcal {L}^\tau )^{-1}:\mathbb {L}^2(\mathbb {P}^\tau )\rightarrow \mathbb {L}^2(\mathbb {P}^\tau )$ is bounded uniformly in τ by $\mu ^{-1}$ and has range given by $\textrm{Dom}(\mathcal {L}^\tau )$.

#### Proof

The proof follows exactly the same steps as that of [15, Lemma 2.6] and is thus omitted. The only point in which some care is needed is in the proof of the strong continuity of the semigroup: in view of the continuity implied by (3.24), it suffices to check it on a dense subset of $\mathbb {L}^\theta (\mathbb {P}^\tau )$, $\theta \in [1,\infty )$, which should here be taken to be the set of bounded cylinder functionals depending on smooth compactly supported test functions. In the above-mentioned reference, bounded cylinder functions depending on more general Schwartz test functions were used instead, but in the present context, a priori, $u^\tau _\cdot \in \mathcal {X}_1$ cannot be tested against these (see Remark 3.3). □

Our next goal is to identify a suitable subset of $\mathbb {L}^2(\mathbb {P})$ (that, by abusing notation, is identified from here on with the Fock space ) which is contained in the domain of the generator, on which the action of $\mathcal {L}^\tau$ is explicit and which contains all those functionals we need in order to establish the convergence stated in our main theorem. In view of the estimates in Section 4.2, it turns out that a convenient choice is that of the subspace $\textbf{T}^\tau$ consisting of  such that for every *n* the Fourier transform of $f_n$ is compactly supported and $f_n\ne 0$ only for finitely many *n*’s. An example of $\phi \in \textbf{T}^\tau$ is  (corresponding to $m! H_m(\eta ^\tau (h))\in \mathbb {L}^2(\mathbb {P}^\tau )$ for $H_m$ the *m*-th Hermite polynomial in (A.1)) for $m\in \mathbb {N}$ and *h* such that $\textrm{Supp}(\mathcal {F}(h))$ is compact. Notice that, even for such a simple example, since *h* cannot have compact support in real space it is not clear that $t\mapsto \phi (u^\tau _t)$ is continuous (see Remark 3.3) and thus Itô’s formula cannot be applied to easily conclude that $\phi \in \textrm{Dom}(\mathcal {L}^\tau )$.

In the next lemma, we prove a slightly more general statement, namely, that cylinder random variables are contained in $\textrm{Dom}(\mathcal {L}^\tau )$ and thus infer that $\textbf{T}^\tau$ is as well.

#### Lemma 3.7

Let τ>0 be fixed and $\mathcal {L}^\tau$ the generator associated to the semigroup $P^\tau$ in (3.23). Then, any cylinder function ϕ, i.e. $\phi =f(\eta ^\tau (h_1),\dots ,\eta ^\tau (h_n))$ for $f:\mathbb {R}^n\rightarrow \mathbb {R}$ smooth and of at most polynomial growth at infinity, and $h_1,\dots ,h_n\in \mathcal {S}(\mathbb {R}^2)$, belongs to $\textrm{Dom}(\mathcal {L}^\tau )$ and we have $\mathcal {L}^\tau \phi (\eta ^\tau )=S^\tau \phi (\eta ^\tau )+A^\tau \phi (\eta ^\tau )$, where

3.25 $$\begin{aligned}  &   S^\tau \phi (\eta ^\tau ){\mathop {=}\limits ^{\tiny \text {def}}}\sum _{i=1}^n \big \langle \frac{1}{2} \nabla \cdot R_\tau \nabla \eta ^\tau , h_i \big \rangle _{L^2(\mathbb {R}^2)} \, \partial _i f(\eta ^\tau (h_1),\dots ,\eta ^\tau (h_n)) \nonumber \\  &   \qquad + \frac{1}{2} \sum _{1 \le i,j \le n} \langle R_{\tau } \nabla h_i, \nabla h_j\rangle _{L^2(\mathbb {R}^2)} \, \partial _i \partial _j f(\eta ^\tau (h_1),\dots ,\eta ^\tau (h_n))\,,\nonumber \\  &   A^\tau \phi (\eta ^\tau ){\mathop {=}\limits ^{\tiny \text {def}}}\sum _{i=1}^n \big \langle \mathcal {N}^\tau _F[\eta ^\tau ], h_i \big \rangle _{L^2(\mathbb {R}^2)} \, \partial _i f(\eta ^\tau (h_1),\dots ,\eta ^\tau (h_n))\,. \end{aligned}$$

As a consequence, $$\textbf{T}^\tau \subset \textrm{Dom}(\mathcal {L}^\tau )$$.

#### Proof

We will first derive the action of $\mathcal {L}^\tau$ on *good* cylinder functionals, i.e. cylinder functionals $\phi =f(\eta ^\tau (h_1),\dots ,\eta ^\tau (h_n))$ such that both *f* and $h_1,\dots ,h_n$ have compact support, and then extend it to cylinder functionals using that $\mathcal {L}^\tau$ is a closed operator as shown in Lemma 3.6.

Let $\phi =f(\eta ^\tau (h_1),\dots ,\eta ^\tau (h_n))$ be a good cylinder functional. Since $v^\tau$ takes values in $\mathcal {C}(\mathbb {R}_+, \mathcal {X}_1)$ and the test functions $h_1,\dots ,h_n$ belong to $\mathcal {C}_c^\infty (\mathbb {R}^2)$, the weak formulation (3.8) and Itô’s formula imply that, $\textbf{P}$-almost surely, for every t≥0

3.26 $$\begin{aligned} \phi (u^\tau _t) = \phi (u_0^\tau ) + \int _0^t L^\tau \phi (u_s^\tau ) \, \textrm{d}s + M^\tau _t(\phi ) \, , \end{aligned}$$

where $L^\tau \phi =S^\tau \phi +A^\tau \phi$ with $S^\tau \phi ,\,A^\tau \phi$ defined according to (3.25), and $(M^\tau _t(\phi ))_{t \ge 0}$ is a true continuous martingale. Notice that the functional $L^\tau \phi (\cdot )$ is continuous on $\mathcal {X}_1$ (so that $L^\tau \phi (u^\tau )$ is $\textbf{P}$-almost surely continuous) and $L^\tau \phi \in \mathbb {L}^{2+\varepsilon }(\mathbb {P}^\tau )$ (so that $\sup _{s \ge 0} \mathbb {E}[|L^\tau \phi (v_s^\tau )|^{2+\varepsilon }] < +\infty$ by stationarity). To check the last claim, recall the definition of $\Vert \cdot \Vert _{\exp }$ in (3.3) and fix $\varepsilon \in (0,\frac{1}{2\kappa }-2)$. Since $\Vert F\Vert _{L^{2+\varepsilon }(\mathcal {N}(0,1))} \lesssim \Vert F\Vert _{\exp }$, it follows that, for any $h \in \mathcal {C}^\infty _c(\mathbb {R}^2)$ and p≥1, it holds

3.27 $$\begin{aligned} \mathbb {E}[| \mathcal {N}^\tau _F[\eta ^\tau ](h)|^{2+\varepsilon }]^{\frac{1}{2+\varepsilon }} \lesssim _{\tau } \Vert F\Vert _{\exp } \, \Vert h\Vert _{L^1(\mathbb {R}^2)} \, ,\quad \text {and}\quad \mathbb {E}[|\eta ^\tau ( h )|^p]^{\frac{1}{p}} \lesssim _{\tau } \Vert h\Vert _{L^1(\mathbb {R}^2)}\,. \end{aligned}$$

Therefore, we can make sense of both $\mathcal {N}^\tau _F[\eta ^\tau ](h)$ and $\eta ^\tau ( h )$ as elements of $\mathbb {L}^2(\mathbb {P})$ for any $h \in L^1(\mathbb {R}^2)$ (and they satisfy (3.27)).

The continuity and integrability of $L^\tau \phi$, together with the continuity of $s\mapsto v^\tau _s$ ensure that both $\textbf{P}$-almost surely and in $\mathbb {L}^2(\mathbb {P}^\tau )$ we have

$$\begin{aligned} \frac{1}{t} \int _0^t L^\tau \phi (v_s^\tau ) \, \textrm{d}s \xrightarrow [t \rightarrow 0]{} L^\tau \phi (v_0^\tau ) \, , \end{aligned}$$

which, by taking conditional expectation with respect to $\mathcal {F}_0$ and using (3.26), leads to

$$\begin{aligned} \frac{P_t^\tau \phi - \phi }{t} \xrightarrow [t \rightarrow 0]{\mathbb {L}^2(\mathbb {P})} L^\tau \phi (v_0^\tau ) \, . \end{aligned}$$

In other words, ϕ belongs to the domain of $\mathcal {L}^\tau$ and $\mathcal {L}^\tau \phi = L^\tau \phi$.

Now, let $\phi ,\phi '$ be two good cylinder functionals with $\phi =f(\eta ^\tau (h_1),\dots ,\eta ^\tau (h_n))$ and $\phi '=f'(\eta ^\tau (h'_1),\dots ,\eta ^\tau (h'_n))$. Thanks to the fact that $\mathcal {L}^\tau \phi = L^\tau \phi$,  (3.27) and Hölder inequality, it is straightforward to check that for every N≥0 it holds that

$$\begin{aligned} \mathbb {E}[&|\phi (\eta ^\tau ) - \phi '(\eta ^\tau )|^2] \vee \mathbb {E}[|\mathcal {L}^\tau \phi (\eta ^\tau ) - \mathcal {L}^\tau \phi '(\eta ^\tau )|^2]\\&\quad \lesssim _{F, \tau , n, N} \max _{1 \le i \le n} \big \{1, \Vert h_i\Vert _{L^1(\mathbb {R}^2)}, \Vert \nabla h_i'\Vert _{L^2(\mathbb {R}^2)}\big \}^{2 N+4} \times \bigg (\sup _{y_{1:n} \in \mathbb {R}^{n}} \frac{|(f' - f)(y_{1:n})|^2}{1+|y_{1:n}|^{2N}}\\&\qquad +\sup _{y_{1:n} \in \mathbb {R}^{n}, \, \alpha \in \{0,3\}^2} \frac{|\partial _\alpha ^{|\alpha |} f(y_{1:n})|^2}{1+|y_{1:n}|^{2N}} \max _{1 \le i \le N }\{\Vert h'_i - h_i\Vert _{L^1(\mathbb {R}^2)}, \Vert \nabla (h_i' - h_i)\Vert _{L^2(\mathbb {R}^2)}\}\bigg ) \, , \end{aligned}$$

which implies that $\mathcal {L}^\tau$ is continuous on the set of good cylinder functionals endowed with its natural Fréchet metric on the set of cylinder functionals. Since the set of good cylinder functionals is clearly dense both in the set of cylinder functionals and in $\textbf{T}^\tau$, and further $\mathcal {L}^\tau$ is closed on $\mathbb {L}^2(\mathbb {P}^\tau )$, it follows that both cylinder functionals and $\textbf{T}^\tau$ belong to the domain of $\mathcal {L}^\tau$ and (3.25) holds, so that the proof is complete. □

We are now ready to derive an explicit expression for the action of $\mathcal {L}^\tau$ on . For n≥0, we write .

#### Lemma 3.8

Let τ>0 be fixed. Let $\mathcal {L}_0^{\mathfrak {e}_1}$, $\mathcal {L}_0^{\mathfrak {e}_2}$ and $\mathcal {S}^\tau$ be the diagonal operators acting on $\psi \in \textbf{T}^\tau _n$ as

3.28 $$\begin{aligned} \mathcal {F}(\mathcal {L}_0^{\mathfrak {e}_i}\psi )\,(p_{1:n})&{\mathop {=}\limits ^{\tiny \text {def}}}-\frac{1}{2}|\mathfrak {e}_i\cdot p_{1:n}|^2 \, \hat{\psi }(p_{1:n})\,,\qquad i=1,2, \end{aligned}$$

3.29 $$\begin{aligned} \mathcal {F}(\mathcal {S}^\tau \psi )\,(p_{1:n})&{\mathop {=}\limits ^{\tiny \text {def}}}\mathcal {F}\big ((\nu _\tau \mathcal {L}_0^{\mathfrak {e}_1}+\mathcal {L}_0^{\mathfrak {e}_2})\psi \big )\,(p_{1:n})=-\frac{1}{2} \, |\sqrt{R_\tau } p_{1:n}|^2 \, \hat{\psi }(p_{1:n}).\nonumber \\ \end{aligned}$$

and, for m≥1, $j \in \{0,...,m-1\}$ and a=m-2j-1, define the operator  which, for any $n \in \mathbb {N}$, maps $\textbf{T}^\tau _n$ to $\textbf{T}^\tau _{n+a}$ and acts on $\psi \in \textbf{T}^\tau _n$ as follows: if j+1>n then , otherwise for m=1,3.30and for m≥2,3.31where for any u≤v positive integers, $\Delta _{u}^{v} {\mathop {=}\limits ^{\tiny \text {def}}}\{(i_1,\dots ,i_u)\in \mathbb {N}^{u}:1 \le i_1<... < i_{u} \le v\}$. We also set3.32the last sum converging in  uniformly on compact subsets of $\textbf{T}^\tau$. Then, on $\textbf{T}^\tau \subset \textrm{Dom}(\mathcal {L}^\tau )$, $\mathcal {L}^\tau$ coincides with $\mathcal {S}^\tau + \mathcal {A}^{\tau }$, $\mathcal {S}^\tau$ is symmetric and each  (and thus also $\mathcal {A}^{\tau }$) is skew-symmetric as it holds  for every m≥1, $a \in \{-m+1, -m+3, \dots , m-1\}$.

Compared to [15], in the present setting the action of the generator is much more involved. Along the lines of the analogy made therein, we can think of  as the span of *n*-particle states, with each particle carrying a momentum $p_i$, and $\mathcal {A}^{\tau }$ as an operator acting on the particles by changing their number and modifying their momenta. In this perspective, while the $\mathcal {A}^{\tau }$ in [15] coincides with  and thus creates (for a=1) and annihilates (for a=-1) *one single* particle, here, $\mathcal {A}^{\tau }$ creates or annihilates an *arbitrarily large number* of particles as the *m* in the sum in (3.32) is unbounded. More precisely, for $m\in \mathbb {N}$, $a\in \{-m+1,-m+3,\dots ,m-1\}$ and *j* such that a=m-2j-1, one can regard , as an operator that takes j+1 particles with momenta $r_1,\dots ,r_{j+1}$ whose sum is $p_{[I]}$, for $I\subset \{1,\dots ,n\}$ of cardinality m-j, and produces m-j particles, whose respective momenta are $\{p_{i}:i\in I\}$.

Since *m* is unbounded it is not a priori obvious that $\mathcal {A}^{\tau }$ in (3.32) is well-defined *even for finite* τ and, to ensure that this is the case, a crucial role will be played by the decay of the coefficients of the Hermite polynomial expansion of the nonlinearity *F*, which in turn is tightly connected to its regularity (see Appendix A). While this is the first problem one encounters in the proof of the above Lemma, the bulk of it consists of showing that the action of $\mathcal {L}^\tau$ on $\textbf{T}^\tau$ in (3.25) indeed coincides with the expressions in (3.29) and (3.32) for which instead we will approximate *F* by a polynomial using Lemma A.6, prove that the statement holds for such *F* (Lemma B.2) and finally verify that we are allowed to remove the approximation in $\mathbb {L}^2(\mathbb {P}^\tau )$.

#### Proof

Before deriving the action of $\mathcal {L}^\tau$ on the Fock space  and show that (3.25) indeed reduces to the formulas in the statement, let us mention that, as a consequence of Lemma B.1, the operators $\mathcal {L}_0^{\mathfrak {e}_1}$, $\mathcal {L}_0^{\mathfrak {e}_2}$, $\mathcal {S}^\tau$ and , for m≥1 and $a \in \{-m+1,-m+3...,m-1\}$, respectively given in (3.28), (3.29), (3.30) and (3.31) are continuous on $\textbf{T}^\tau$. For $\mathcal {A}^{\tau }$ in (3.32), the argument is more subtle and relies on the quantitative estimate (B.1) and Assumption 1.1. Indeed, for $n\in \mathbb {N}$ and $\psi \in \textbf{T}^\tau _n$, the former impliesand, since by Proposition A.3 and Assumption 1.1$c_m(F)/\sqrt{m!}$ decays faster than any inverse power, the sum at the r.h.s. is bounded (by an *n*-dependent constant) and thus, $\mathcal {A}^{\tau }$ is well-defined and continuous from $\textbf{T}^\tau$ to .

It remains to show that for $\phi \in \textbf{T}^\tau$, $\mathcal {L}^\tau \phi =\mathcal {S}^\tau \phi + \mathcal {A}^{\tau }\phi$ and that $\mathcal {S}^\tau$ and $\mathcal {A}^{\tau }$ satisfy the stated symmetry properties. In view of their continuity, it suffices to prove them for ϕ in a dense subset of $\textbf{T}^\tau$ which we take to be the set of ϕ of the form $I_n^\tau (h^{\otimes n}) = n! H_n( \eta ^\tau (h))$ for $n\in \mathbb {N}$ and *h* such that $\hat{h}\in \mathcal {C}_c^\infty (\mathbb {R}^2)$ and $\Vert h\Vert _{L^2_\tau (\mathbb {R}^2)} = 1$. Notice that such ϕ is a cylinder functional so that, by Lemma 3.7, $\phi \in \textrm{Dom}(\mathcal {L}^\tau )$ and $\mathcal {L}^\tau \phi =S^\tau \phi +A^\tau \phi$, with $S^\tau \phi ,\,A^\tau \phi$ as in (3.25). As shown in, e.g., [30], the Fock space representation of $S^\tau \phi$ coincides with $\mathcal {S}^\tau \phi$ in (3.29), which is clearly symmetric, so that we are left to verify that $A^\tau \phi =\mathcal {A}^{\tau }\phi$ and that the latter is skew-symmetric. Now, when *F* is a polynomial, both properties hold thanks to Lemma B.2 and the linearity of $\mathcal {A}^{\tau }$, and we now show that this implies the same is true for any *F* satisfying Assumption 1.1, via an approximation argument.

Denote by $A_P^\tau \phi$ the quantity $A^\tau \phi$ in (3.25) with F=P and *P* polynomial and assume that, for any *P*, $A_P^\tau \phi =\mathcal {A}_P^\tau \phi {\mathop {=}\limits ^{\tiny \text {def}}}\sum _{m \ge 1} c_m(P) \, \mathcal {A}^{\tau , m} \phi$, with the sum at the r.h.s. having only finitely many terms. Let $(P_M)_{M \ge 1}$ be a sequence of polynomials converging to *F* in the sense of Lemma A.6. The claim follows provided we prove that, as $M\rightarrow \infty$, the sequences $(A_{P_M}^\tau \phi )_M$ and $(\mathcal {A}_{P_M}^\tau \phi )_M$ converge respectively to $A^\tau \phi$ and $\mathcal {A}^\tau \phi$, in $\mathbb {L}^2(\mathbb {P}^\tau )$. For the former, notice that for any $\varepsilon \in (0,\frac{1}{2\kappa },2)$

$$\begin{aligned} \mathbb {E}[| \mathcal {N}^\tau _F[\eta ^\tau ](h)-\mathcal {N}^\tau _{P_M}[\eta ^\tau ](h)|^{2+\varepsilon }]^{\frac{1}{2+\varepsilon }} =\mathbb {E}[| \mathcal {N}^\tau _{F-P_M}[\eta ^\tau ](h)|^{2+\varepsilon }]^{\frac{1}{2+\varepsilon }} \lesssim _{\tau } \Vert F-P_M\Vert _{\exp } \, \Vert h\Vert _{L^1(\mathbb {R}^2)} \end{aligned}$$

where in the last step we applied (3.27) with $F-P_M$ in place of *F* and the norm $\Vert \cdot \Vert _{\exp }$ was defined in (3.3). Hence, by Lemma A.6 the r.h.s. converges to 0 and, using the representation (3.25) and Hölder’s inequality, we conclude that $A_{P_M}^\tau \phi \rightarrow A^\tau \phi$ as $M\rightarrow \infty$ in $\mathbb {L}^2(\mathbb {P}^\tau )$. For the other, recalling the definition of the coefficients $c_m$ in (1.17) and using the estimate (B.1), we havewhere we applied Lemma A.1 with ℓ=m-1 for m≤n+3 and ℓ=n+3 elsewhere. The quantity in parenthesis is bounded (and its value depends on *n*) and the supremum converges to 0 by Lemma A.6. □

As we will see in the next section, the main ingredient in the proof of our main result is the convergence of the resolvent of $u^\tau$ to that of $u^{\textrm{eff}}$ in (1.13) in . To do so, one is led to estimating first the -norm of the (difference of the) resolvent(s), as this is the norm that naturally appears when testing the resolvent equation with its solution (recall that $\mathcal {A}^{\tau }$ is skew-symmetric), in terms of the -norm (by duality of  and ) of the function to which the resolvent is applied. However, this is only possible provided , which ensures that the -norm of the resolvent is indeed bounded. While in [15] Lemma 2.10 therein verifies such condition, in the present context, the singularity of $\mathcal {A}^{\tau }$ due to the sum defining it in (3.32) having infinitely many terms, makes it unclear whether this is the case (and even if the sum were up to a finite *m* with m>2, we would not know how to prove it)^4^. That said, as the next lemma shows, the so-called Itô’s trick in [26] allows us to bypass the problem as it directly proves that the resolvent is continuous from  to  uniformly in τ.

#### Lemma 3.9

There exists a constant C>0 independent of τ such that for every  it holds that3.33

#### Proof

Since $(1-\mathcal {L}^\tau )^{-1}$ is bounded by 1 from  to itself by Lemma 3.6, a density argument ensures that it suffices to prove (3.33) on a dense subspace of . For reasons that will become clear in what follows, we will take it to be the set of functionals ϕ such that $(1-\mathcal {S}^\tau )\psi =\phi$ with ψ a *good* cylinder functional as those used in the proof of Lemma 3.7, i.e. $\psi =f(\eta ^\tau (h_1),\dots ,\eta ^\tau (h_n))$ such that *f* and $h_1,\dots ,h_n$ are smooth with compact support.

Notice first that, by integration by parts and the contractivity bound (3.24), the resolvent satisfies

$$\begin{aligned} (1 - \mathcal {L}^\tau )^{-1} \phi = \int _0^{+\infty } e^{-t} P^\tau _t \phi \, \textrm{d}t = \int _0^{+\infty } e^{-t} \Big (\int _0^t P^\tau _s \phi \, \textrm{d}s\Big ) \, \textrm{d}t\,,\end{aligned}$$

where $P^\tau$ is the semigroup in (3.23). As a consequence, denoting by $u^\tau$ the unique mild solution to (3.1) driven by $(\eta ^\tau , \vec {\xi }^\tau )$, we get

3.34 $$\begin{aligned} \Vert (1 - \mathcal {L}^\tau )^{-1} \phi \Vert _\tau \!\le \! \Big \Vert \int _0^{+\infty } e^{-t} \mathbb {E}\Big [\int _0^t \phi (u^\tau _s) \, \textrm{d}s\Big |\mathcal {F}_0\Big ] \, \textrm{d}t\Big \Vert _\tau \!\le \! \int _0^\infty e^{-t}\textbf{E}\bigg [\Big |\int _0^t \phi (u^\tau _s) \, \textrm{d}s\Big |^2\bigg ]^{\frac{1}{2}}\textrm{d}t \end{aligned}$$

and our goal is to bound the inner expectation in terms of the -norm of ϕ. To do so, we will exploit the same strategy first presented in the proof of the celebrated Itô’s trick in [26].

Proposition 3.2 and Itô’s formula give

$$\begin{aligned} \psi (u_t^\tau ) - \psi (u_0^\tau ) - \int _0^t \mathcal {S}^\tau \psi (u_s^\tau ) \, \textrm{d}s - \int _0^t \mathcal {A}^\tau \psi (u_s^\tau ) \, \textrm{d}s = M_t^\tau (\psi ) \, , \end{aligned}$$

where we further used that $\psi \in \textbf{T}^\tau$ so that, by Lemma 3.8, $\mathcal {L}^\tau \psi =\mathcal {S}^\tau \psi +\mathcal {A}^{\tau }\psi$, and $M^\tau (\psi )$ is a true continuous martingale whose quadratic variation can be readily checked to satisfy

$$\begin{aligned} \textbf{E}[\langle M^\tau (\psi ), M^\tau (\psi ) \rangle _t] = 2 t \, \Vert (- \mathcal {S}^\tau )^{1/2} \psi \Vert _\tau ^2\,. \end{aligned}$$

The very definition of quadratic variation then implies

3.35 $$\begin{aligned} \textbf{E}\Big [\Big |\psi (u_t^\tau ) - \psi (u_0^\tau ) - \int _0^t \mathcal {S}^\tau \psi (u_s^\tau ) \, \textrm{d}s - \int _0^t \mathcal {A}^\tau \psi (u_s^\tau ) \, \textrm{d}s\Big |^2\Big ] \le 2 t \, \Vert (- \mathcal {S}^\tau )^{1/2} \psi \Vert _\tau ^2 \, . \end{aligned}$$

The same argument applied to the mild solution $\tilde{u}^\tau$ of (3.1) with -F in place of *F* together with the fact that, thanks to Proposition 3.2, $(\tilde{u}^\tau _s)_{s\in [0,t]}{\mathop {=}\limits ^{\tiny \text {law}}}(u^\tau _{t-s})_{s\in [0,t]}$, also gives

3.36 $$\begin{aligned} \textbf{E}\Big [\Big |\psi (u_0^\tau ) - \psi (u_t^\tau ) - \int _0^t \mathcal {S}^\tau \psi (u_s^\tau ) \, \textrm{d}s + \int _0^t \mathcal {A}^\tau \psi (u_s^\tau ) \, \textrm{d}s\Big |^2\Big ] \le 2 t \, \Vert (- \mathcal {S}^\tau )^{1/2} \psi \Vert _\tau ^2 \, . \end{aligned}$$

Summing (3.35) and (3.36) (and using the basic inequality $2a^2+2b^2\ge (a+b)^2$, $a,b\in \mathbb {R}$), we end up with

$$\begin{aligned} \textbf{E}\Big [\Big |\int _0^t (-\mathcal {S}^\tau ) \psi (u_s^\tau ) \, \textrm{d}s\Big |^2\Big ] \le 2t \, \Vert (- \mathcal {S}^\tau )^{1/2} \psi \Vert _\tau ^2\,. \end{aligned}$$

We bound the l.h.s. from below as

$$\begin{aligned} \textbf{E}\Big [\Big |\int _0^t (-\mathcal {S}^\tau ) \psi (u_s^\tau ) \, \textrm{d}s\Big |^2\Big ]&\ge \frac{1}{2}\textbf{E}\Big [\Big |\int _0^t (1-\mathcal {S}^\tau ) \psi (u_s^\tau ) \, \textrm{d}s\Big |^2\Big ]-\textbf{E}\Big [\Big |\int _0^t \psi (u_s^\tau ) \, \textrm{d}s\Big |^2\Big ]\\&\ge \frac{1}{2}\textbf{E}\Big [\Big |\int _0^t (1-\mathcal {S}^\tau ) \psi (u_s^\tau ) \, \textrm{d}s\Big |^2\Big ]-t^2\Vert \psi \Vert ^2_\tau \end{aligned}$$

the last step being a consequence of Jensen’s inequality. Now, rearranging the terms and recalling that $(1-\mathcal {S}^\tau )\psi =\phi$, deducewhich can be plugged at the r.h.s. of (3.34) to obtain (3.33). □

#### Remark 3.10

Let us point out that the proof of the previous lemma uses only the stationarity of $u^\tau$ and the boundedness of its resolvent so that its statement holds much more generally.

## Proof of the main result

This section represents the core of the paper as it contains the novel ideas and the crucial estimates thanks to which we can prove our main results, Theorems 1.3 and 1.4. Before delving into the details, let us heuristically present the necessary steps and provide a roadmap of what will follow.

### Main ideas and roadmap

The bulk of the proof of Theorems 1.3 and 1.4 consists of showing that $\mathcal {L}^\tau$ converges to $\mathcal {L}^\textrm{eff}$, the generator of $u^\textrm{eff}$ in (1.13), in the resolvent sense, i.e. that for a sufficiently large class of  we formally have

4.1 $$\begin{aligned} \lim _{\tau \rightarrow \infty }\Vert (1-\mathcal {L}^\tau )^{-1}\phi -(1-\mathcal {L}^\textrm{eff})^{-1}\phi \Vert =0\,. \end{aligned}$$

We will not prove (4.1) directly as we have very little control of the resolvent of $\mathcal {L}^\tau$. Instead, since according to the heuristic presented in the introduction we expect the quadratic component of the nonlinearity *F* (corresponding to its projection onto the space generated by the second Hermite polynomial) to be dominant, we will proceed in two steps and show instead that both the following limits hold

4.2 $$\begin{aligned} \lim _{\tau \rightarrow \infty }\Vert (1-\mathcal {L}^\tau )^{-1}\phi -(1-\mathcal {L}^{\tau ,2})^{-1}\phi \Vert&=0\,, \end{aligned}$$

4.3 $$\begin{aligned} \lim _{\tau \rightarrow \infty }\Vert (1-\mathcal {L}^{\tau ,2})^{-1}\phi -(1-\mathcal {L}^\textrm{eff})^{-1}\phi \Vert&=0 \end{aligned}$$

where  is the generator of the solution of (3.1) with nonlinearity $\mathcal {N}_G^\tau$ with $G(x)=c_2(F)x^2$. The validity of (4.3) is a direct consequence of what was shown in [15]. To see why we can expect (4.2) to be satisfied, notice that by (3.33), we (formally) have4.4where  (and the last equality explains why we chose *G* as such). To develop some intuition as to why the r.h.s. should vanish, let us replace $(1-\mathcal {L}^{\tau ,2})^{-1}\phi$ by a function ψ in . Then, by Lemma 3.8, since j+1≤n=1 implies j=0,  only if a=m-1, so that . By orthogonality of Fock spaces with different indices (and neglecting m=1 for the sake of the present discussion), we get4.5where we used (3.31). Now, the inner integral can be bounded as

$$\begin{aligned} \int _{p_{[1:m]}=p} \frac{ \Xi _{m}^\tau (\textrm{d}p_{1:m})}{1+\frac{1}{2}|\sqrt{R_\tau } p_{1:m}|^2}&= \tau ^{m-2} \nu _\tau ^{\frac{1-m}{2}}\int _{p_{[1:m]}=\tau ^{1/2}R_\tau ^{-\frac{1}{2}}p}\; \frac{ \Xi _{m}^1(\textrm{d}p_{1:m})}{\tau ^{-1}+\frac{1}{2}|p_{1:m}|^2}\\&\le (2\pi )^{m-1}\tau ^{m-2} \nu _\tau ^{\frac{1-m}{2}}\int _{\mathbb {R}^4}\; \frac{ \Xi _{2}^1(\textrm{d}p_{1:2})}{\frac{1}{2}|p_{1:2}|^2} \end{aligned}$$

and the last integral is finite and independent of *m*. Plugging it back into (4.5), we conclude4.6where the norm on *F* is defined in (A.3). Therefore, provided the anisotropic norm of ψ at the r.h.s. converges to 0, as is the case since $\psi \in H^1(\mathbb {R}^2)$, then so does the -norm of , thus suggesting that the operator  should not contribute to the limit.

Even though the arguments above give a useful indication as to what we should expect, they are by no means enough. Let us mention that computations similar to those in (4.5) were first presented in [28], where the authors proved a universality statement for the SBE in dimension d=1 in the so-called weak coupling scaling. But while for them, studying functionals in the first chaos was sufficient, in the present setting this is *not the case*. As a matter of fact, there are several difficulties we need to overcome:

(I) **Chaos Growth:** to bound the r.h.s. of (4.4), we need to control the -norm of  applied to $(1-\mathcal {L}^{\tau ,2})^{-1}\phi$. Now, even for ϕ in the first chaos, the latter will have non-trivial components in *every* chaos. This means that we need to estimate -norm of  for *every* *m* *and* *a*, when applied to rather general elements of the Fock space. As it turns out (and true even for m=2 and a=±1), these operators naturally introduce a dependence in the so-called number operator (see Definition 2.2), which in turn has to be tamed.
(II) **Well-posedness:** the first equality in (4.4) is *not rigorous*. Indeed, for it to make sense we should know that $(1-\mathcal {L}^{\tau ,2})^{-1}\phi$ belongs to the domain of $\mathcal {L}^\tau$, which is all but obvious. According to Lemma 3.8, we only know that $\textbf{T}^\tau \subset \textrm{Dom}(\mathcal {L}^\tau )$, for $\textbf{T}^\tau$ the set of  such that for every *n* the Fourier transform of $f_n$ is compactly supported and $f_n\ne 0$ only for finitely many *n*’s, but $(1-\mathcal {L}^{\tau ,2})^{-1}\phi \notin \textbf{T}^\tau$.

This being said, in order to prove (4.1), there was no need to choose *precisely* $(1-\mathcal {L}^{\tau ,2})^{-1}\phi$ in (4.2) and (4.3) in the first place. To retain (at least part of) the argument above, and in particular that in (4.4) and the results of [15], all we need is an Ansatz that *approximates* $(1-\mathcal {L}^{\tau ,2})^{-1}\phi$ *sufficiently well*. Let us mention that, already in [15], an Ansatz was introduced to show (4.3), but we want to modify it in such a way that, for every fixed τ, it belongs to $\textbf{T}^\tau$ (so that II holds). More precisely, our Ansatz $s^\tau (\phi ) =(s^\tau _n(\phi ))_{n\in \mathbb {N}}$ will have finite chaos expansion, i.e. $s^\tau _n(\phi ) \equiv 0$ for $n \ge \bar{n}$ with $\bar{n}$ suitably depending on τ, and, for each *n*, $s^\tau _n(\phi )$ will have compactly supported Fourier transform, whose radius will depend on *both* *n* *and* τ. It is this latter property that will allow us to tame the growth in the chaos and ultimately overcome point I above.

The rest of the paper is organised as follows. In Section 4.2, we derive fine estimates on , for $\phi \in \textbf{T}^\tau$, that keep track of the size of the support of the Fourier transform of the kernels of ϕ. This is the place at which the Assumption 1.1 on *F* is used in its full strength. Then, in Section 4.3, we recall the Ansatz introduced in [15], describe the novel Ansatz $s^\tau (\phi )$ and show it still satisfies (the analogue of) (4.2) and (4.3). At last, Section 4.4 is devoted to the proof of Theorems 1.3 and 1.4.

### Estimates for the non-quadratic part of the generator

In the following proposition we estimate the -norm of , for every m≠2 and $a\in \{-m+1,\dots , m+1\}$. The elements to which these operators are applied belong to $\textbf{T}^\tau$ and there are two crucial aspects whose dependence in the bounds we want to keep track of:

1. the size of the support of the Fourier transform of their kernels, and
2. the anisotropic -norm of ϕ, i.e. $\Vert (-\nu _\tau \mathcal {L}_0^{\mathfrak {e}_1})^{\frac{1}{2}}\phi \Vert$ (see (4.6)).

The first is essential to tame the growth in the number operator, the latter guarantees that these terms stand a chance of having a vanishing contribution (see (4.6) again).

#### Proposition 4.1

There exists a constant C>0 such that for every τ>0, $m,\, n\in \mathbb {N}$, m≠2, $\chi \in [0,1]$ and  for which $\textrm{Supp}(\hat{\phi })\subset \{p_{1:n} \, :\, |\sqrt{R_\tau p_{1:n}}|^2 \le \tau \chi \}$, it holds that4.7for any $\alpha \in (0,1)$.

#### Proof

Let us first deal with the case m=1. From (3.30), we havewhere the factor *n*! comes from the definition of the -norm (see (2.7) with the $H^{-1}_\tau$-norm in (2.6) in place of the $L^2_\tau$ one). Recall from (2.4) the definition of $\Theta ^\tau$, i.e. $\Theta ^\tau (p) = \big (2 \pi \hat{\rho }(\tau ^{-1/2} \sqrt{R_\tau } p)\big )^2$, and that ρ is smooth, compactly supported and of mass 1 so that $\Theta ^\tau (0) = 1$. This means that $\Theta ^\tau$ is globally Lipschitz and we have $|\Theta ^\tau (p) - 1| \lesssim \tau ^{-1/2} \, |\sqrt{R_\tau } p|$ uniformly in $p\in \mathbb {R}^2$. Thus, Cauchy–Schwarz inequality leads to

$$\begin{aligned} \tau \nu _\tau \, \frac{\big (\sum _{i=1}^n \mathfrak {e}_1 \cdot p_i \, (\Theta ^\tau (p_i) - 1)\big )^2}{1 + |\sqrt{R_\tau } p_{1:n}|^2/2} \lesssim \nu _\tau \frac{|\mathfrak {e}_1 \cdot p_{1:n}|^2 \, |\sqrt{R_\tau } p_{1:n}|^2}{1+|\sqrt{R_\tau } p_{1:n}|^2} \le \nu _\tau |\mathfrak {e}_1 \cdot p_{1:n}|^2 \, \end{aligned}$$

from which we immediately deduce (4.7) with a prefactor independent of *n*.

Now, let m≥3, $j \in \{0, \dots , m-1\}$ and a=m-2j-1. From (3.31), we get

4.8 $$\begin{aligned} \begin{aligned} \Vert \mathscr {A}_{a}^{\tau , m} \varphi \Vert _{\Gamma H_{\tau }^{-1}}^{2}&= (n+a)! \frac{n!^2}{(n+a)!^2 (j+1)!^2} \frac{\tau ^{-m+2} \nu _{\tau }^{\frac{m+1}{2}}}{(2\pi )^{2(m-1)}} \int \frac{\Xi _{n+a}^{\tau }(\textrm{d}p_{1:n+a})}{1 + \frac{1}{2}|\sqrt{R_{\tau }} p_{1:n+a}|^2} \\&\quad \times \left| \sum _{I \in \Delta _{m-j}^{n+a}} \textbf{e}_1 \cdot p_{[I]} \int _{r_{[1:j+1]} = p_{[I]}} \Xi _{j+1}^{\tau }(\textrm{d}r_{1:j+1}) \hat{\varphi }(r_{1:j+1}, p_{1:n+a \setminus I}) \right| ^2. \end{aligned} \end{aligned}$$

To take into account the assumption that ϕ has compactly supported Fourier transform, let $V_\chi ^\tau {\mathop {=}\limits ^{\tiny \text {def}}}\bigsqcup _{k \ge 0} \{x_{1:k} \in \mathbb {R}^{2k} \,, \ |\sqrt{R_\tau } x_{1:k}|^2 \le \chi \, \tau \}$, so that in particular $\textrm{Supp}(\hat{\phi })\subset V_\chi ^\tau$. Note that $V_\chi ^\tau$ is stable under restriction of components meaning that, for subsets $A \subseteq B\subset \mathbb {N}$, if $p_B=(p_i)_{i\in B} \in V_\chi ^\tau$, then so does $p_A$. Hence, for any $p_{1:n}\in \mathbb {R}^{2n}$ and $I\subset \{1:n\}$, we have

4.9 $$\begin{aligned} \hat{\phi }(p_{1:n})=1\!\!1_{p_I\in V^\tau _\chi } \hat{\phi }(p_{1:n})\,, \end{aligned}$$

a fact that will be repeatedly used throughout the proof. With this in mind, let us expand the square in (4.8)

4.10 $$\begin{aligned} \Big |&\sum _{I \in \Delta _{m-j}^{n+a}} \mathfrak {e}_1 \cdot p_{[I]} \int _{r_{[1:j+1]} = p_{[I]}} \Xi _{j+1}^\tau (\textrm{d}r_{1:j+1}) \, \hat{\phi }(r_{1:j+1}, p_{1:n+a\setminus I})\Big |^2\nonumber \\&\le \sum _{I,J \in \Delta _{m-j}^{n+a}} 1\!\!1_{p_{I\setminus J}\in V^\tau _\chi }|\mathfrak {e}_1 \cdot p_{[I]}|\int _{r_{[1:j+1]} = p_{[I]}} \Xi _{j+1}^\tau (\textrm{d}r_{1:j+1}) \, |\hat{\phi }(r_{1:j+1}, p_{1:n+a\setminus I})|\nonumber \\&\qquad \qquad \times 1\!\!1_{p_{J\setminus I}\in V^\tau _\chi }|\mathfrak {e}_1 \cdot p_{[J]}|\int _{r_{[1:j+1]} = p_{[J]}} \Xi _{j+1}^\tau (\textrm{d}r_{1:j+1}) \, |\hat{\phi }(r_{1:j+1}, p_{1:n+a\setminus J})|\nonumber \\&\le \left( {\begin{array}{c}n+a\\ m-j\end{array}}\right) \sum _{I \in \Delta _{m-j}^{n+a}} 1\!\!1_{p_{I \setminus \{1:m-j\} \in V_{\chi }^{\tau }}} |\mathfrak {e}_1 \cdot p_{[I]}|^2 \times \nonumber \\&\qquad \qquad \times \Big (\int _{r_{[1:j+1]} = p_{[I]}} \Xi _{j+1}^\tau (\textrm{d}r_{1:j+1}) \, |\hat{\phi }(r_{1:j+1}, p_{1:n+a\setminus I})|\Big )^2 \end{aligned}$$

where, in the first step, we used (4.9) and that both I\J and $J\setminus I\subseteq (\{1:n+a\}\setminus I)\cup (\{1:n+a\}\setminus J)$, while in the second the trivial bound $2ab\le a^2+b^2$ (fixing w.l.o.g. $J=\{1:m-j\}$) and that the cardinality of $\Delta _{m-j}^{n+a}$ is $$\left( {\begin{array}{c}n+a\\ m-j\end{array}}\right) $$. To bound the square of the integral, let $\delta ,\beta \in [0,1]$ be arbitrary for now (we will choose them later on) and apply Cauchy–Schwarz, so that

4.11 $$\begin{aligned} \bigg (&\int _{r_{[1:j+1]} = p_{[I]}} \Xi ^\tau _{j+1}(\textrm{d}r_{1:j+1}) \, |\hat{\phi }(r_{1:j+1}, p_{1:n+a \setminus I})|\bigg )^2 \nonumber \\&\le \bigg (\int _{ r_{[1:j+1]} = p_{[I]}} \Xi ^\tau _{j+1}(\textrm{d}r_{1:j+1}) \, (\nu _\tau |\mathfrak {e}_1 \cdot r_{1:j+1}|^2)^\delta |\sqrt{R_\tau } r_{1:j+1}|^{2 \beta } \, |\hat{\phi }( r_{1:j+1}, p_{1:n+a \setminus I})|^2\bigg ) \nonumber \\&\qquad \times \bigg (\int _{\tilde{r}_{[1:j+1]} = p_{[I]}} \Xi _{j+1}^\tau (\textrm{d}\tilde{r}_{1:j+1}) \frac{1\!\!1_{\tilde{r}_{1:j+1} \in V_{\chi }^{\tau } }}{(\nu _\tau |\mathfrak {e}_1 \cdot \tilde{r}_{1:j+1}|^2)^\delta |\sqrt{R_\tau } \tilde{r}_{1:j+1}|^{2 \beta }}\bigg )\nonumber \\&\le \bigg (\int _{ r_{[1:j+1]} = p_{[I]}} \Xi ^\tau _{j+1}(\textrm{d}r_{1:j+1}) \, (\nu _\tau |\mathfrak {e}_1 \cdot r_{1:j+1}|^2)^\delta |\sqrt{R_\tau } r_{1:j+1}|^{2 \beta } \, |\hat{\phi }( r_{1:j+1}, p_{1:n+a \setminus I})|^2\bigg ) \nonumber \\&\qquad \times \bigg (\int \Xi _{j}^\tau (\textrm{d}\tilde{r}_{1:j}) \frac{1\!\!1_{\tilde{r}_{1:j} \in V_{\chi }^{\tau } }}{(\nu _\tau |\mathfrak {e}_1 \cdot \tilde{r}_{1:j}|^2)^\delta |\sqrt{R_\tau } \tilde{r}_{1:j}|^{2 \beta }}\bigg ) \end{aligned}$$

where we inserted the indicator function of $\{r_{1:j+1}\in V^\tau _\chi \}$ for free thanks to (4.9) and in the last bound we removed an integration variable and the corresponding constraint on the integral. For the scalar product multiplying the square in (4.10), notice that, since $p_{[I]}=r_{[1:j+1]}$, we can estimate $|\mathfrak {e}_1 \cdot p_{[I]}|^2 \le (m-j) |\mathfrak {e}_1 \cdot p_{I}|^2$ and $|\mathfrak {e}_1 \cdot p_{[I]}|^2 \le (j+1) |\mathfrak {e}_1 \cdot r_{1:j+1}|^2$. Therefore, since further $(m-j)\vee (j+1)\le m+1$, for any $\gamma \in [0,1]$, we get

$$\begin{aligned} |\mathfrak {e}_1 \cdot p_{[I]}|^2\le (m+1) |\mathfrak {e}_1 \cdot p_{I}|^{2(1-\gamma )}|\mathfrak {e}_1 \cdot r_{1:j+1}|^{2\gamma } \end{aligned}$$

which then gives

4.12 $$\begin{aligned} \frac{ |\mathfrak {e}_1 \cdot p_{[I]}|^2}{1 + |\sqrt{R_\tau } p_{1:n+a}|^2/2} \lesssim \nu _\tau ^{-1}(m+1) \, \frac{(\nu _\tau |\mathfrak {e}_1 \cdot r_{1:j+1}|^2)^{\gamma }}{|\sqrt{R_\tau } p_{I}|^{2 \gamma }} \, . \end{aligned}$$

Plugging (4.10), (4.11) and (4.12) into (4.8), relabelling the variables $(r_{1:j+1}, p_{1:n+a\setminus I})=q_{1:n}$ and exploiting the multiplicative structure of $\Xi ^\tau _{n+a}$, we deduce4.13where in the second step we applied the change of variables $\tau ^{-\frac{1}{2}}\sqrt{R_\tau }\tilde{r}_i=x_i$, i=1,⋯,j+1 to the integral over $\tilde{r}_{1:j+1}$ and similarly for that over $p_{I}$. For $\delta ,\beta ,\gamma \in [0,1]$ and $I\in \Delta ^{n+a}_{m-j}$, $\textbf{A}^j_{\delta ,\beta }$ and $\textbf{B}_{\gamma }^I$ are the (essentially) τ-independent integrals defined by

4.14 $$\begin{aligned} \textbf{A}^j_{\delta ,\beta }&{\mathop {=}\limits ^{\tiny \text {def}}}\int \Xi _{j}^1(\textrm{d}x_{1:j}) \frac{1\!\!1_{x_{1:j} \in V_{\chi }^{1} }}{|\mathfrak {e}_1 \cdot x_{1:j}|^{2\delta } | x_{1:j}|^{2 \beta }}\,, \end{aligned}$$

4.15 $$\begin{aligned} \textbf{B}^I_{\gamma }&{\mathop {=}\limits ^{\tiny \text {def}}}\int _{y_{[I]}=\tau ^{-\frac{1}{2}}\sqrt{R_\tau }q_{[1:j+1]}}\Xi _{m-j}^1(\textrm{d}y_{I}) \frac{1\!\!1_{y_{I\setminus \{1:m-j\}}\in V^1_\chi }}{|y_I|^{2\gamma }}\,. \end{aligned}$$

We now choose $\delta ,\beta ,\gamma \in [0,1]$ so that (4.14) and (4.15) are finite and bounded by a quantity that, at least for large enough *m*, depends explicitly on χ. To do so, we will consider four different cases: (1) $j\ge 3,\,m\ge 4$; (2) j=2; (3) j=1; and (4) j=0. In each of them, our choice, which is neither unique nor optimal, will be made to minimise the value of β as this is responsible for the full -norm of ϕ at the r.h.s. of (4.7) (see (4.20)). Our bound is obtained by the following procedure: remove from the denominator the maximal number of variables for which the resulting integral is finite; remove from the indicator function those variables which are still at the denominator; and integrate one of the remaining variables (provided there is one) using the trivial estimate

4.16 $$\begin{aligned} \int \Xi ^1_1(\textrm{d}z) 1\!\!1_{z\in V^1_\chi }=(2\pi )^2 \int \hat{\rho }(z)^2 1\!\!1_{z\in V^1_\chi } \lesssim (2\pi )^2 \chi \,. \end{aligned}$$

More concretely, in case (1) (i.e. $j\ge 3,\,m\ge 4$) we take $\beta =\gamma =0$, and estimate $\textbf{A}^j_{\delta ,0}$ as

4.17 $$\begin{aligned} \textbf{A}^j_{\delta ,0}\le \int \frac{\Xi _{3}^1(\textrm{d}x_{1:3})}{|\mathfrak {e}_1 \cdot x_{1:3}|^{2\delta }} \bigg (\int \Xi _{1}^1(\textrm{d}x) 1\!\!1_{x \in V_{\chi }^{1}} \bigg )^{j-3}\lesssim (2\pi )^{2j}\chi ^{1\!\!1_{j\ge 4}} \end{aligned}$$

the last bound being a consequence of the fact that for any $x\in \mathbb {R}^2$, $\mathfrak {e}_1\cdot x$ is a one-dimensional variable (meaning that the first integral is effectively over $\mathbb {R}^3$) so that it holds for any $\delta \in [0,1]$, while for $\textbf{B}^I_{0}$ we have

4.18 $$\begin{aligned} \textbf{B}^I_{0}\lesssim \int _{y_{[I]}=\tau ^{-\frac{1}{2}}\sqrt{R_\tau }q_{[1:j+1]}}\Xi _{m-j}^1(\textrm{d}y_{I}) 1\!\!1_{y_{I\setminus \{1:m-j\}}\in V^1_\chi }\lesssim (2\pi )^{2(m-j-1)}\chi ^{1\!\!1_{|I\setminus \{1:m-j\}|\ge 2}}\,. \end{aligned}$$

In case (2), i.e. j=2, we take γ=0, and bound $\textbf{B}^I_{0}$ as in (4.18), and $\textbf{A}^j_{\delta ,\beta }$ as

$$\begin{aligned} \textbf{A}^2_{\delta ,\beta }\le \int \frac{\Xi _{2}^1(\textrm{d}x_{1:2})}{|\mathfrak {e}_1 \cdot x_{1:2}|^{2\delta } | x_{1:2}|^{2 \beta }}\le \int \frac{\Xi _{2}^1(\textrm{d}x_{1:2})}{|\mathfrak {e}_1 \cdot x_{1:2}|^{2\delta } | \mathfrak {e}_2\cdot x_{1:2}|^{2 \beta }} \end{aligned}$$

that is bounded by $(2\pi )^2$ for any $\delta ,\beta$ such that $\delta \vee \beta <1$.

In case (3), i.e. j=1, take β=0, so that

$$\begin{aligned} \textbf{A}^1_{\delta ,0}&\le \int \frac{\Xi _{1}^1(\textrm{d}x_1)}{|\mathfrak {e}_1 \cdot x_{1}|^{2\delta } }\lesssim (2\pi )^2 \end{aligned}$$

for any δ<1/2, and

4.19 $$\begin{aligned} \textbf{B}_{\gamma }^I\le \int _{y_{[I]}=\tau ^{-\frac{1}{2}}\sqrt{R_\tau }q_{[1:2]}}\Xi _{m-1}^1(\textrm{d}y_{I}) \frac{1\!\!1_{y_{I\setminus \{1:m-1, i_1,i_2\}}\in V^1_\chi }}{|(y_{i_1},y_{i_2})|^{2\gamma }}\lesssim (2\pi )^{2(m-2)}\chi ^{1\!\!1_{|I\setminus \{1:m-j\}|\ge 3}}\,, \end{aligned}$$

where we took $i_1,\,i_2\in I$, and the last bound is valid for any $\gamma \in [0,1]$.

At last, in case (4), i.e. j=0, $\textbf{A}^j_{\delta ,\beta }=1$ as there is no integral (so we take $\delta =\beta =0$), and the other is estimated as in (4.19) for an arbitrary $\gamma \in [0,1]$.

Overall, getting back to (4.13), we have shown that, for suitable values of $\delta ,\beta ,\gamma \in [0,1]$, and since a=m-2j-1, we have4.20where in the last line we used monotonicity of the binomial. Now, in cases (1), (3) and (4), we take β=0 and $\delta +\gamma =1$ so that the second line simply equals $\Vert (- \nu _\tau \mathcal {L}_0^{\mathfrak {e}_1})^{\frac{1}{2}}\phi \Vert _\tau ^{2}\le \Vert (- \nu _\tau \mathcal {L}_0^{\mathfrak {e}_1})^{\frac{1}{2}}\phi \Vert _\tau ^{2\alpha }\Vert (- \mathcal {S}^\tau )^{\frac{1}{2}} \phi \Vert _\tau ^{2(1-\alpha )}$ which holds for any $\alpha \in [0,1]$. In case (2) instead, we choose $\alpha {\mathop {=}\limits ^{\tiny \text {def}}}\delta +\gamma \in (0,1)$, $\beta {\mathop {=}\limits ^{\tiny \text {def}}}1-\alpha$, and apply Hölder’s inequality with exponents 1/α and 1/(1-α), and thus

$$\begin{aligned} n! \int \Xi ^\tau _{n}(\textrm{d}q_{1:n}) (\nu _\tau |\mathfrak {e}_1\cdot q_{1:j+1}|^2)^{\alpha }&|\sqrt{R_\tau }q_{1:j+1}|^{2(1-\alpha )} |\hat{\phi }(q_{1:n})|^2\\&\le \Vert (- \nu _\tau \mathcal {L}_0^{\mathfrak {e}_1})^{\frac{1}{2}}\phi \Vert _\tau ^{2\alpha } \Vert (- \mathcal {S}^\tau )^{\frac{1}{2}} \phi \Vert _\tau ^{2(1-\alpha )}\,. \end{aligned}$$

As a consequence, upon summing the previous over $j\in \{0,m-1\}$, we have shown that, for every $\alpha \in (0,1)$, we haveand, as the sum can be bounded as

$$\begin{aligned} \sum _{j = 0}^{m+1} \left( {\begin{array}{c}n\\ j\end{array}}\right) \left( {\begin{array}{c}m+1\\ j\end{array}}\right) \chi ^{1\!\!1_{\ell \ge 4}} \lesssim n^3 m^3 + \chi \, 4^n \, m^{n} \,, \end{aligned}$$

the statement follows at once. □

In order to deduce a bound on  from Proposition 4.1, it remains to use the fact that , for  defined in (3.32), and choose suitably the way in which the size of the Fourier support of the elements of $\textbf{T}^\tau$ grows with the number operator so that Assumption 1.1 can be exploited.

#### Corollary 4.2

Under Assumption 1.1, for every $\phi \in \textbf{T}^\tau$ for which, for every n≥1, $\textrm{Supp}(\hat{\phi }_n)\subset \{p_{1:n} \, :\, |\sqrt{R_\tau p_{1:n}}|^2 \le \tau \chi _n\}$ with $\chi _n=\exp (-\exp (c n))$ for some c>0, there exists a constant C=C(c)>0 such that, for any $\alpha \in (0,1)$, we have4.21

#### Proof

In essence, the statement is a consequence of Proposition 4.1 and Proposition A.5. Indeed, we haveand we can estimate the sum at the r.h.s. by using (A.10). In formulas, for every γ>0, we have

$$\begin{aligned} \sum _{m\ge 3} \frac{|c_m(F)|}{\sqrt{m!}} \Big (n^{3} m^{4} + \chi _n \, 4^n m^{n+1}\Big ) \lesssim _\gamma n^{3} + \chi _n \, 4^{n} \exp (\exp (n\gamma /2)) \end{aligned}$$

so that, since $\chi _n=\exp (-\exp (c n))$, it suffices to choose $\gamma \in (0,2c)$, and the statement follows at once. □

### The amended Ansatz for the resolvent of the quadratic Burgers equation

Before presenting the modification we want to perform, let us recall the original Ansatz introduced in [15, Sec. 4.1] for the solution of the Burgers equation with quadratic nonlinearity, and state some of the properties it enjoys. For a heuristic explaining how it was deduced, we refer the interested reader to [15, Sec. 2.3].

Let ϕ be an element of  for some fixed $n_0 \ge 1$. We define $\tilde{s}^\tau (\phi )$ according to [15, Eq. (4.7)], i.e.4.22where, for M≥0, $\mathcal {G}_M^\tau$ is the diagonal operator, according to Definition 2.1, in [15, Eq. (3.18)] given by

4.23 $$\begin{aligned} \mathcal {G}_M^\tau {\mathop {=}\limits ^{\tiny \text {def}}}\big (1 - \mathcal {S}^\tau + \mathcal {g}_M^\tau \big )^{-1}\qquad \text {with}\qquad \mathcal {g}_M^\tau {\mathop {=}\limits ^{\tiny \text {def}}}-g_M^\tau (L^\tau (- \mathcal {S}^\tau )) \, \mathcal {L}_0^{\mathfrak {e}_1}\end{aligned}$$

for $g_M^\tau (x){\mathop {=}\limits ^{\tiny \text {def}}}(M \nu _\tau ) \vee g^\tau (x)$, and $g^\tau$ and $L^\tau$ the functions on $\mathbb {R}_+$ respectively defined by

4.24 $$\begin{aligned} g^\tau (x) {\mathop {=}\limits ^{\tiny \text {def}}}\big (\tfrac{3}{2} \, x + \nu _\tau ^{3/2}\big )^{2/3} - \nu _\tau \quad \text {and}\quad L^\tau (x) {\mathop {=}\limits ^{\tiny \text {def}}}\frac{c_2(F)^2 \nu _\tau ^{3/2}}{\pi } \log \Big (1 + \frac{\tau }{1+x}\Big )\,, \end{aligned}$$

while  is the operator in [15, Eq. (3.7)] whose action in Fourier on a given element  reads4.25for $\chi ^{\sharp ,\tau }$ the multiplier

4.26 $$\begin{aligned} \chi _{q,q'}^{\sharp ,\tau }(p_{1:N}) {\mathop {=}\limits ^{\tiny \text {def}}}1\!\!1_{|\sqrt{R_\tau } q| \wedge |\sqrt{R_\tau } q'| > 2 \, |\sqrt{R_\tau } p_{1:N}|} \, . \end{aligned}$$

For future reference, let us also define  as4.27

#### Remark 4.3

Compared to [15], we adapted the notations to be in line with those in the present paper: $\tilde{s}^\tau (\phi )$ above is $s^\tau$ therein; the operators  above correspond to the operators $\mathcal {A}^{\sharp , \tau }_{\pm },\,\mathcal {A}^{\flat , \tau }_{\pm }$; the generic coupling constant λ>0, which multiplied the nonlinearity in [15, Eq. (1.1)] has been replaced in each of its occurrences by $c_2(F)$ and omitted from the definition of  (this is the reason why it appears as a multiplicative factor in (4.22)).

With the previous definitions at hand, we can now recall the main properties of $\tilde{s}^\tau (\phi )$. We will provide a thorough proof below providing precise references to [15, Sec. 4] for the details.

#### Proposition 4.4

Assume $c_2(F)\ne 0$. Let $n_0\in \mathbb {N}$, $\phi \in \textbf{T}^\tau _{n_0}$ be such that $\textrm{Supp}(\mathcal {F}\phi ) \subset \{- \mathcal {S}^\tau \le R\}$ for some R>2, and $\tilde{s}^\tau (\phi )$ be defined according to (4.22). Then, for every $\delta \in (0,1/4]$ there exists constants $C=C(c_2(F), n_0, R, M, \delta )>0$ and q=q(δ)>0 independent of τ such that4.284.29for $\mathcal {N}$ the number operator in Definition 2.2, and further4.304.31where .

#### Proof

Let us separately discuss each of the estimates (4.28)–(4.31), starting from (4.29).

*Estimate* (4.29). It is a direct consequence of [15, Prop. 4.8 (and Rem. 4.10)] as can be seen upon taking $q\in [0, \log \sqrt{2})$, $w(k){\mathop {=}\limits ^{\tiny \text {def}}}e^{2q k}$ so that the function $f^\tau _w$ in [15, Eq. (4.22)] reads $f_w^\tau (x) = \nu _\tau ^{1-\frac{1}{2}e^{2q}} \, (\nu _\tau + g^\tau (x))^{e^{2q}/2}$. Indeed, [15, Eq. (4.21)] gives

$$\begin{aligned} e^{-q\,n_0}\Vert e^{-q\mathcal {N}} (-\nu _\tau \mathcal {L}_0^{\mathfrak {e}_1})^{\frac{1}{2}}\tilde{s}^\tau (\phi )\Vert _\tau \le 2 \Vert \big (-f^\tau _w(L^\tau (- \mathcal {S}^\tau )) \, \mathcal {L}_0^{\mathfrak {e}_1}\big )^{1/2}\mathcal {G}^\tau _M\phi \Vert _\tau \lesssim \nu _\tau ^{\frac{1}{2}\left( 1-\frac{1}{2}e^{2q}\right) }\Vert \phi \Vert _\tau \end{aligned}$$

where in the last step we used that the operators $-f^\tau _w(L^\tau (- \mathcal {S}^\tau )) \, \mathcal {L}_0^{\mathfrak {e}_1}$ and $\mathcal {G}^\tau _M$ commute, that

$$\begin{aligned} \mathcal {G}^\tau _M&=\big (1-\mathcal {S}^\tau -g_M^\tau (L^\tau (- \mathcal {S}^\tau ))\mathcal {L}_0^{\mathfrak {e}_1}\big )^{-1} \le \big (1-\mathcal {S}^\tau -g^\tau (L^\tau (- \mathcal {S}^\tau ))\mathcal {L}_0^{\mathfrak {e}_1}\big )^{-1} \\&=\big (1-\mathcal {L}_0^{\mathfrak {e}_2}-[\nu _\tau +g^\tau (L^\tau (- \mathcal {S}^\tau ))]\mathcal {L}_0^{\mathfrak {e}_1}\big )^{-1}\le \big (1-[\nu _\tau +g^\tau (L^\tau (- \mathcal {S}^\tau ))]\mathcal {L}_0^{\mathfrak {e}_1}\big )^{-1} \end{aligned}$$

and $\mathcal {G}_M^\tau \le 1$, and that, as a consequence, for any $q\in [0,\log \sqrt{2})$, we get

$$\begin{aligned} (-f^\tau _w(L^\tau (- \mathcal {S}^\tau )) \, \mathcal {L}_0^{\mathfrak {e}_1})^{1/2}\mathcal {G}^\tau _M&\le (-f^\tau _w(L^\tau (- \mathcal {S}^\tau )) \, \mathcal {L}_0^{\mathfrak {e}_1})^{1/2}(\mathcal {G}^\tau _M)^{1/2}\\&\le \nu _\tau ^{\frac{1}{2}\left( 1-\frac{1}{2}e^{2q}\right) }[\nu _\tau +g^\tau (L^\tau (0))]^{\frac{1}{2}\left( e^{2q}-1\right) }\lesssim \nu _\tau ^{\frac{1}{2}\left( 1-\frac{1}{2}e^{2q}\right) }\,. \end{aligned}$$

*Estimate* (4.28). It follows by [15, Eq. (4.13)], (4.28) and the fact that $\mathcal {G}_M^\tau$ is a bounded operator.

*Estimate* (4.30). See Eq. (4.14) in [15, Prop. 4.3].

*Estimate* (4.31). This estimate is not explicitly written in [15] but can be easily deduced from the results therein. Let us provide a few details. By [15, Lem. 4.2], $\tilde{s}^\tau$ satisfieswhere . Let us separately consider each summand. For the first, we exploit the Replacement Lemma [15, Lem. 3.6], which givesFor the second, we use instead [15, Prop. 3.4], obtainingand at last, the bound on the third follows by Eq. (3.31) in [15, Prop. 3.12]. Collecting these estimates, and using (4.29) once again, one can easily deduce (4.31). □

What the previous proposition says is that, on the one hand, $\tilde{s}^\tau (\phi )$ is a good approximation of $(1-\mathcal {L}^{\tau ,2})^{-1}\phi$ (by (4.31)) and, on the other, it is close in  to the resolvent of the effective equation (1.13) applied to ϕ. This latter point follows by (4.30) and the fact that, as $\tau \rightarrow \infty$, the operator $\mathcal {G}^\tau _M$ converges to $(1-\mathcal {L}^\textrm{eff})^{-1}$ as argued in [15, Eq. (3.20)].

In other words, $\tilde{s}^\tau (\phi )$ satisfies most of the main properties we desired an ansatz to have in Section 4.1. At the same time, it lacks arguably the most important: its chaos expansion is infinite and its kernels do not have a compactly supported Fourier transform. Thus, the results of Section 4.2 are not applicable, which in turn implies that we are unable to control the r.h.s. of (4.4).

To remedy this, we introduce an *amended Ansatz* $s^\tau$ which is obtained from $\tilde{s}^\tau (\phi )$ by truncating the support of the Fourier transform (and the chaos) of $\tilde{s}^\tau (\phi )$ in such a way that the assumptions of Corollary 4.2 are satisfied. More precisely, let us define4.32where the r.h.s. in the definition of  is the diagonal operator whose Fourier kernel on the *n*-th chaos is $1\!\!1_{\{ n \le -\frac{1}{c} \log \nu _\tau , \, |\sqrt{R_\tau } p_{1:n}|^2 \le \tau \exp (-\exp (cn)) \}}$, and c>0 is a parameter to be chosen later.

The next proposition is the core of our analysis as it is the one that shows that indeed the amended ansatz tames both I and II and provides an approximation thanks to which we will be able to control the norms in (4.2) and (4.3).

#### Proposition 4.5

Assume $c_2(F)\ne 0$. Let $n_0\in \mathbb {N}$, $\phi \in \textbf{T}^\tau _{n_0}$ be such that $\textrm{Supp}(\mathcal {F}\phi ) \subset \{- \mathcal {S}^\tau \le R\}$ for some R>0. For every $\varepsilon \in (0,1/8]$, there exists $c_0 = c_0(\varepsilon ) > 0$ such that the following holds. Given $c \in (0, c_0)$, define $s^\tau (\phi )$ according to (4.32). First, $s^\tau (\phi ) \in \textbf{T}^\tau$ for every τ>0. Moreover, there exist $\tau _0 > 0$ and a constant $C=C(c_2(F), n_0, R, M, c, \tau _0, \varepsilon )>0$ independent of τ such that, for every $\tau \ge \tau _0$, we have4.334.34

#### Remark 4.6

Let us mention that the choice of $s^\tau (\phi )$ is, clearly, *not unique*. In principle, any element $s^\tau (\phi ) \in \textbf{T}^\tau$ such that both (4.33) and (4.34) hold, would do. The difficulty lies precisely in the ability to *find at least one* such element and we exhibit an explicit one.

#### Remark 4.7

While the statements at the beginning of the section (see (4.2), (4.3)) were purely qualitative, the previous proposition is *quantitative* in that the bounds in (4.33) and (4.34) provide *rates* at which the norms at the l.h.s. converge to 0. That said, we do not expect them to be optimal (especially the second).

In the proof of the above proposition, we will need the following lemma that allows to control the terms arising from the commutator of the operators  in (4.32) and , $\bullet \in \{\sharp ,\flat \}$, in (4.25), (4.27).

#### Lemma 4.8

Let c>0. Then, there exists a constant C=C(c)>0 such that for any sufficiently regular  and $\beta \in (0,1/2)$ we have4.35where ,  as in (4.32), and , for $\bullet \in \{\sharp ,\flat \}$, are the operators in (4.25), (4.27).

#### Proof

Clearly, it suffices to consider  for some $n\in \mathbb {N}$. To lighten the notations, set $\chi _n{\mathop {=}\limits ^{\tiny \text {def}}}\exp (-\exp (c\,n))$ and let $E_1^\tau (\mathcal {N}){\mathop {=}\limits ^{\tiny \text {def}}}\{\mathcal {N}> -\frac{1}{c}\log \nu _\tau \}$ and $E_2^\tau (\mathcal {N}){\mathop {=}\limits ^{\tiny \text {def}}}\{- 2 \mathcal {S}^\tau > \tau \,\chi _n\}$, so that , where we recall that the Fourier representation of $\mathcal {S}^\tau$ in (3.29) clearly depends on the order of the Fock space to which it is applied. We will split the argument in four steps.

*Step 1.* Notice that, for any $\beta ,\gamma \in (0,1)$ with $\beta +\gamma =1$, we have $(1-\mathcal {S}^\tau )^{-1}\le (-\nu _\tau \mathcal {L}_0^{\mathfrak {e}_1})^{-\beta }(-\mathcal {S}^\tau )^{-\gamma }$, so that we can writeWe now focus on the latter norm. As, trivially, $1\!\!1_{E_1^\tau (\mathcal {N})\cup E_2^\tau (\mathcal {N})}\le 1\!\!1_{E_1^\tau (\mathcal {N})}+1\!\!1_{E_2^\tau (\mathcal {N})}$ and, by construction, , the triangle inequality and a simple duality argument imply that if we show that, for k=1,2, there exists $f_k(\tau ,n)>0$ such that for all $n\in \mathbb {N}$ and $\psi \in \textbf{T}^\tau _n$4.36then the same bounds will hold with .

*Step 2.* To verify (4.36), let us begin with a preliminary computation of its l.h.s.. Setting $\chi ^{\flat ,\tau }{\mathop {=}\limits ^{\tiny \text {def}}}1-\chi ^{\sharp ,\tau }$, and $\chi ^{\bullet ,\tau }$ to be either $\chi ^{\sharp ,\tau }$ or $\chi ^{\flat ,\tau }$, the definition of the operator  in (4.25) gives4.37The next two steps describe how to estimate the r.h.s. of (4.37) in the cases k=1 or 2.

*Step 3.* Note that, for k=1, then $E_1^\tau (n)\subset E_1^\tau (n+1)$, so that $(1\!\!1_{E_k^\tau (n+1)})^\theta (1\!\!1_{E_k^\tau (n)})^{1-\theta }\le 1\!\!1_{E_k^\tau (n)}$. Thus, the integral in *q* is upper bounded by

$$\begin{aligned} 1\!\!1_{E_k^\tau (n)} \nu _\tau ^{1/2} \int _{\mathbb {R}^2} \frac{\textrm{d}q \,\Theta ^1(\tau ^{-1/2} \sqrt{R^\tau } q) }{|\sqrt{\nu _\tau } \mathfrak {e}_1 \cdot q|^{2\beta } (1+|\sqrt{R^\tau } q|^{2})^{\gamma }}&=1\!\!1_{E_k^\tau (n)}\nu _\tau ^{1/2} \int _{\mathbb {R}^2} \frac{\textrm{d}q \, \Theta ^1(q) }{| \mathfrak {e}_1 \cdot q|^{2\beta } (\tau ^{-1}+|q|^{2})^{\gamma }}\lesssim \nu _\tau ^{-1} \end{aligned}$$

from which, since $\nu _\tau |\mathfrak {e}_1 \cdot p_{1:n}|^2\le (\nu _\tau |\mathfrak {e}_1 \cdot p_{1:n}|^2)^{\beta }(1+|\sqrt{R^\tau }p_{1:n}|^2)^\gamma$, we ultimately deduce that (4.36) holds with $f_1(\tau ,n) {\mathop {=}\limits ^{\tiny \text {def}}}n^3 1\!\!1_{E_k^\tau (n)} \nu _\tau ^{-\frac{1}{2}}$ .

*Step 4.* For k=2 instead, we claim that (4.37) can be estimated from above by

4.38 $$\begin{aligned} \,&n^3 n! \int \Xi _n^\tau (\textrm{d}p_{1:n}) \, \big (\nu _\tau |\mathfrak {e}_1 \cdot p_1|^2\big ) \, |\hat{\psi }(p_{1:n})|^2 \nu _\tau ^{\frac{1}{2}} \int \textrm{d}q \, \Theta ^1(\tau ^{-1/2} \sqrt{R^\tau } q) \Theta ^1(\tau ^{-1/2} \sqrt{R^\tau } (p_1-q))\nonumber \\&\qquad \qquad \quad \times \frac{1\!\!1_{|\sqrt{R^\tau } q|^2 \gtrsim \tau \chi _n /n} + 1\!\!1_{|\sqrt{R^\tau } (p_1-q)|^2 \gtrsim \tau \chi _n /n} + 1\!\!1_{|\sqrt{R^\tau } q|^2 \le |\sqrt{R^\tau } p_{1:n}|^2}}{|\sqrt{\nu _\tau } \mathfrak {e}_1 \cdot (q,p_1-q,p_{2:n})|^{2\beta } |\sqrt{R^\tau } (q,p_1-q,p_{2:n})|^{2\gamma }}\,. \end{aligned}$$

To see this, observe that if θ=1, then $|\sqrt{R^\tau } (q,p_1-q,p_{2:n})|^2>\tau \chi _{n+1}$ which implies that

4.39 $$\begin{aligned} |\sqrt{R^\tau }q|^2\vee |\sqrt{R^\tau }(p_1-q)|^2\vee \max _{\ell \in \{2,\dots ,n\}} |\sqrt{R^\tau }p_\ell |^2\ge \frac{\tau \chi _{n+1}}{n+1}\gtrsim \frac{\tau \chi _{n}}{n} \end{aligned}$$

where the last bound depends on *c*. On the other hand, if θ=0, then $|\sqrt{R^\tau } p_{1:n}|^2>\tau \chi _{n}$ so that $\max _{\ell \in \{1,\dots ,n\}} |\sqrt{R^\tau }p_\ell |^2\ge \tau \chi _{n}/n$. This means that either $\max _{\ell \in \{2,\dots ,n\}} |\sqrt{R^\tau }p_\ell |^2\ge \tau \chi _{n}/n$ or $|\sqrt{R^\tau }p_1|^2\ge \tau \chi _{n}/n$, and in this latter case, by the triangle inequality, $|\sqrt{R^\tau }q|^2\vee |\sqrt{R^\tau }(p_1-q)|^2\gtrsim \tau \chi _{n}/n$. In other words, no matter whether θ=0 or 1, (4.39) holds. Now, there are two scenarios: (1) $|\sqrt{R^\tau }q|^2\vee |\sqrt{R^\tau }(p_1-q)|^2\gtrsim \tau \chi _{n}/n$; (2) $|\sqrt{R^\tau }p_\ell |^2\gtrsim \tau \chi _{n}/n$ for some $\ell \in \{2,\dots ,n\}$. If we are in the setting of (2), then either $|q|\wedge |p_1-q|\ge |p_\ell |$ so that we fall back into (1), or $|q|\vee |p_1-q|\le |p_\ell |$ thus implying that $|\sqrt{R^\tau }q|^2\le |\sqrt{R^\tau }p_\ell |^2\le |\sqrt{R^\tau } p_{1:n}|^2$. In conclusion, we have one of this three possibilities which correspond to each of the indicator functions at the r.h.s. of (4.38): $|\sqrt{R^\tau }q|^2\gtrsim \tau \chi _{n}/n$; $|\sqrt{R^\tau }(p_1-q)|^2\gtrsim \tau \chi _{n}/n$; $|\sqrt{R^\tau }q|^2\le |\sqrt{R^\tau } p_{1:n}|^2$.

Getting back to (4.38), we estimate the integral in *q* separately for each summand corresponding to a different indicator function. The first two allow for the same bound (by symmetry) which is

4.40 $$\begin{aligned} \nu _\tau ^{\frac{1}{2}}\int \textrm{d}q&\frac{1\!\!1_{|\sqrt{R^\tau } q|^2 \gtrsim \tau \chi _{n}/n} \Theta ^1(\tau ^{-1/2} \sqrt{R^\tau } q) \Theta ^1(\tau ^{-1/2} \sqrt{R^\tau } (p_1-q))}{|\sqrt{\nu _\tau } \mathfrak {e}_1 \cdot (q,p_1-q,p_{2:n})|^{2\beta } (1+|\sqrt{R^\tau } (q,p_1-q,p_{2:n})|^2)^{\gamma }}\nonumber \\&\le \nu _\tau ^{\frac{1}{2}}\int \textrm{d}q \frac{1\!\!1_{|\sqrt{R^\tau } q|^2 \gtrsim \tau \chi _n /n} \Theta ^1(\tau ^{-1/2} \sqrt{R^\tau } q) }{|\sqrt{\nu _\tau } \mathfrak {e}_1 \cdot q|^{2\beta } |\sqrt{R^\tau } q|^{2\gamma }}= \int \textrm{d}q \frac{1\!\!1_{|q|^2 \gtrsim \chi _n /n} \Theta ^1( q) }{|\mathfrak {e}_1 \cdot q|^{2\beta } |q|^{2\gamma }} \nonumber \\&\lesssim \int _{\sqrt{\frac{\chi _n}{n}}}^\infty \frac{\textrm{d}r}{r(1+r^4)}\int _0^{2\pi }\frac{\textrm{d}\theta }{\cos (\theta )^{2\beta }}\lesssim \log (n/\chi _n)\lesssim e^{c \,n} \end{aligned}$$

where we used the decay properties of $\Theta ^1$ to insert $(1+r^4)$ at the denominator and the second to last step holds provided 2β<1. For the third instead, we note that $|\mathfrak {e}_1 \cdot (q,p_1-q,p_{2:n})|\gtrsim |\mathfrak {e}_1 \cdot p_{1:n}|$ and $|\sqrt{R^\tau } (q,p_1-q,p_{2:n})|\gtrsim |\sqrt{R^\tau } p_{1:n}|$, so that

4.41 $$\begin{aligned} \nu _\tau ^{\frac{1}{2}}\int \textrm{d}q&\frac{1\!\!1_{|\sqrt{R^\tau } q|^2 \le |\sqrt{R^\tau } p_{1:n}|^2} \Theta ^1(\tau ^{-1/2} \sqrt{R^\tau } q) \Theta ^1(\tau ^{-1/2} \sqrt{R^\tau } (p_1-q))}{|\sqrt{\nu _\tau } \mathfrak {e}_1 \cdot (q,p_1-q,p_{2:n})|^{2\beta } (1+|\sqrt{R^\tau } (q,p_1-q,p_{2:n})|^2)^{\gamma }}\nonumber \\&\lesssim \frac{\nu _\tau ^{\frac{1}{2}}}{(\sqrt{\nu _\tau }|\mathfrak {e}_1 \cdot p_{1:n}|)^{2\beta }|\sqrt{R^\tau } p_{1:n}|^{2\gamma }}\int \textrm{d}q 1\!\!1_{|\sqrt{R^\tau } q|^2 \le |\sqrt{R^\tau } p_{1:n}|^2}\lesssim \frac{|\sqrt{R^\tau } p_{1:n}|^{2\beta }}{(\sqrt{\nu _\tau }|\mathfrak {e}_1 \cdot p_{1:n}|)^{2\beta }} \end{aligned}$$

since $$\beta +\gamma =1$$.

Plugging (4.40) and (4.41) back into (4.38), it is not hard to conclude that (4.36) holds with $f_2(n,\tau )=\exp (c\,n)$.

*Step 5.* Let us now inspect the r.h.s. of (4.36). As we did in the last step of (4.20), we apply Hölder’s inequality with exponents 1/β and 1/γ and obtainand now, for k=2, the definition of $f_2$ immediately gives the required upper bound in (4.35), while for k=1, we note that

$$\begin{aligned} f_1(\tau ,\mathcal {N})^{\frac{1}{\beta }}=n^{\frac{3}{\beta }}1\!\!1_{n>-\frac{1}{c}\log \nu _\tau }\nu _\tau ^{-\frac{1}{2\beta }}\lesssim e^{\frac{c}{\beta }\,n} e^{-\frac{c}{\beta }\log \nu _\tau ^{-\frac{1}{2c}}} \nu _\tau ^{-\frac{1}{2\beta }}\lesssim e^{\frac{c}{\beta }\,n} \end{aligned}$$

and the same conclusion holds. □

We are now ready to complete the proof of Proposition 4.5.

#### Proof of Proposition 4.5

The double truncation in the definition of $s^\tau (\phi )$ in (4.32) immediately implies that, for every τ>0 fixed, it belongs to $\textbf{T}^\tau$.

Concerning (4.33), note that, as ϕ has compactly supported Fourier transform and $\mathcal {G}^\tau _M$ is diagonal, for τ large enough, . Since $s^\tau (\phi )$ is obtained via a hard Fourier cut-off of $\tilde{s}^\tau (\phi )$ in (4.22) and, by removing the truncation, we make the -norm bigger, we getwhere the last step is a consequence of (4.30).

A similar argument also shows that the  and the weighted anisotropic norms of $s^\tau (\phi )$ can be controlled in terms of those of $\tilde{s}^\tau (\phi )$, i.e.4.424.43thanks to (4.28) and (4.29).

We now turn our attention to (4.34). Since $s^\tau (\phi )\in \textbf{T}^\tau$ and $\textbf{T}^\tau$ is contained in the domain of $\mathcal {L}^\tau$ by Lemma 3.7 we can compute $\mathcal {L}^\tau s^\tau (\phi )$ and, in view of Lemma 3.8, the action of $\mathcal {L}^\tau$ can be explicitly written in terms of $\mathcal {S}^\tau$ in (3.29) and  in (3.32). More precisely, we have4.44where  and  is the operator in (4.32). The first term is controlled by (4.31), while, for the last, we can use Corollary 4.2 as $s^\tau (\phi )$ satisfies all of its assumptions. In particular, (4.21) implies that, for every $\alpha \in (0,1)$,where we used (4.43) and (4.42). It remains to look at the second summand, which is bounded by4.45A priori, it is not immediately clear how to control the -norm of . Indeed, if we simply neglect the cut-off, then what we obtain is of order one by (4.28). But, by [15, Lem. 4.2] (adapted to our notations, see Remark 4.3), we know thatApplying  at both sides, the summand containing ϕ vanishes for τ large enough, thus we deducewhere we used that  and $\mathcal {G}^\tau _M$ commute, and that, by its definition, $\mathcal {G}^\tau _M\le (1-\mathcal {S}^\tau )^{-1}$. Plugging the above estimate into (4.45), we reach a position in which we can apply Lemma 4.8. Its statement gives that, assuming c∈(0,q/2), we have for every $\beta \in (0,1/2)$where in the last step we exploited (4.28), (4.29), which is allowed since we have taken c<q/2.

Collecting all the observations made so far, choosing β sufficiently close to 1/2 and defining ε accordingly, (4.34) follows and the proof of the proposition is thus complete. □

### Resolvent Convergence and proof of Theorems 1.3 and 1.4

In this subsection, we complete the proof of the main results of the paper, Theorems 1.3 and 1.4. Before turning to it, let us highlight in the next theorem the main consequence of the analysis carried out so far (from which both main results will follow as a corollary), namely the convergence of $\mathcal {L}^\tau$ to $\mathcal {L}^\textrm{eff}$, the generator of (1.13), in the resolvent sense. Its proof makes rigorous sense of the analysis sketched in Subsection 4.1 and heavily relies on Proposition 4.5 and the definition of the operator $\mathcal {G}^\tau _M$ in (4.23).

#### Theorem 4.9

For every $F\in \mathbb {L}^2(\mathbb {P})$, we have

4.46 $$\begin{aligned} \iota ^\tau (1 - \mathcal {L}^\tau )^{-1} j^\tau F \xrightarrow [\tau \rightarrow \infty ]{\mathbb {L}^2(\mathbb {P})} (1 - \mathcal {L}^{\textrm{eff}})^{-1} F\,, \end{aligned}$$

where $\iota ^\tau$ and $j^\tau$ are the maps in Definition 2.3 and $\mathcal {L}^\textrm{eff}$ is the generator of (1.13), i.e. the diagonal operator whose action on  coincides with that of $D^\textrm{eff}_{1,1}\mathcal {L}_0^{\mathfrak {e}_1}+\mathcal {L}_0^{\mathfrak {e}_2}$ for $D^\textrm{eff}_{1,1}$ the first entry of the matrix $D^\textrm{eff}$ in (1.14).

#### Proof

As noted in the proof of [15, Thm. 5.1], it suffices to check (4.46) on a dense subset of $\mathbb {L}^2(\mathbb {P})$, e.g. that which consists of elements of the form $I_{n_0}(\phi )$ for $n_0\in \mathbb {N}$ and ϕ smooth and with compactly supported Fourier transform. For such an observable, let $s^\tau (\cdot )$ be our amended ansatz as given in (4.32) and note thatwhere we denoted by $\Vert \cdot \Vert$ the norm on  (without the τ), we used [15, Lem. 1.7] to pass from  to  and to replace $\iota ^\tau \mathcal {G}^\tau _M j^\tau$ with $\mathcal {G}^\tau _M$ (this is a slight abuse of notation, but the two operators have the same kernels) and at last we applied Lemma 3.9 to estimate the -norm with the -norm. Now, thanks to Proposition 4.5, and in particular (4.33) and (4.34), the first two summands go to 0. For the last instead, we exploit the observation made in [15, Eq. (3.20)] according to which the Fourier transform of the kernel of $\mathcal {g}^\tau _M$ converges pointwise in the limit for $\tau , M\rightarrow \infty$ to that of $D^\textrm{eff}_{1,1}\mathcal {L}_0^{\mathfrak {e}_1}$. As a consequence, the kernel of $\mathcal {G}^\tau _M$ converges in the same sense to that of $(1-D^\textrm{eff}_{1,1}\mathcal {L}_0^{\mathfrak {e}_1}-\mathcal {L}_0^{\mathfrak {e}_2})^{-1}=(1-\mathcal {L}^\textrm{eff})^{-1}$. Since ϕ is smooth, by the dominated convergence theorem, the conclusion follows at once. □

We are now ready to present the proof of Theorems 1.3 and 1.4.

#### Proof of Theorems 1.3 and 1.4

In light of Theorem 4.9, the proof follows the exact same steps as that of Theorems 1.3 and 1.4 detailed in [15, Sec. 5]. The proof therein uses in no special way the specific structure of the equation or of its generator but only the analog of Theorem 4.9 and properties of the limiting equation. Since our limiting equation is the same, that proof applies here *mutatis mutandis*.

To witness, an immediate consequence of Theorem 4.9 and the Trotter–Kato theorem [40, Thm. 4.2] is the convergence of the semigroup $P^\tau$ to that of the effective equation, $P^\textrm{eff}$, in $\mathbb {L}^2(\mathbb {P})$ uniformly on compact time intervals. The extension to infinite time horizons and to $\mathbb {L}^\theta (\mathbb {P})$, for $\theta \in [1,\infty )$ which would imply (1.15), is given in [15, *Part I*, pg. 45]. It only relies on the contractivity of $P^\tau$ (which we have by Lemma 3.6) and of $P^\textrm{eff}$, and on mixing properties of the latter (see [15, Lem. 2.11]), all of which hold in the present context as well.

The argument in [15, *Part II*, pg. 46] ensures that (1.15) implies that if for $\theta \in [1, +\infty )$ and k≥1 $(F_1^\tau )_{\tau> 0},..., (F_k^\tau )_{\tau > 0}$ converge to $F_1,..., F_k$ in $\mathbb {L}^\theta (\mathbb {P})$ then for any times $0 \le t_1 \le \dots \le t_k$,

4.47 $$\begin{aligned} \int \left| \textbf{E}_{u_0}^\tau \left[ F_1^\tau (u_{t_1}^\tau ) \dots F_k^\tau (u_{t_k}^\tau ) \right] - \textbf{E}_{u_0}^{\text {eff}} \left[ F_1 (u_{t_1}^{\text {eff}}) \dots F_k (u_{t_k}^{\text {eff}}) \right] \right| ^\theta \mathbb {P}(\textrm{d} u_0) \rightarrow 0. \end{aligned}$$

Hence, upon taking $F^\tau _i(\eta ^\tau ) {\mathop {=}\limits ^{\tiny \text {def}}}f_i(\eta ^\tau (\phi _i))$, for i=1,⋯,k, $\phi _1,\dots ,\phi _k\in \mathcal {S}(\mathbb {R}^2)$ and $f_1,\dots ,f_k$ bounded continuous functions on $\mathbb {R}$, we immediately deduce Theorem 1.3 for $\mathbb {Q} = \mathbb {P}$; the extension to $\mathbb {Q} \ll \mathbb {P}$ is then obvious.

But in (4.47), there is no need to take the $f_i$’s to be *bounded*, thus we also deduce that (1.16) holds for *f* polynomial. To move to a generic *f* continuous and of at most polynomial growth, we proceed by approximation. Indeed, for such *f*, there exists a sequence of polynomials $(P_n)_{n \ge 1}$ such that for every c>0 it holds that

$$\begin{aligned} \sup _{x_{1:k}\in \mathbb {R}^k} \frac{|f(x_{1:k}) - P_n(x_{1:k})|}{\sum _{i=1}^k e^{c x_i^2} } \rightarrow 0 \,, \qquad \hbox { as}\ n \rightarrow \infty . \end{aligned}$$

But then, by Jensen’s inequality and stationarity, we get

$$\begin{aligned} \int \mathbb {P}(\textrm{d}u_0) \,&\Big |\textbf{E}_{u_0}^\tau \big [(f - P_n)\big (u^{\tau }_{t_1}(\phi _1), \dots , u^{\tau }_{t_k}(\phi _k)\big )\big ] - \textbf{E}_{u_0}^{\textrm{eff}}\big [(f-P_n)\big (u^{\textrm{eff}}_{t_1}(\phi _1), \dots , u^{\textrm{eff}}_{t_k}(\phi _k)\big )\big ]\Big |^p\\&\le \textbf{E}^\tau \big [|f - P_n|^p\big (u^{\tau }_{t_1}(\phi _1), \dots , u^{\tau }_{t_k}(\phi _k\big )\big ] + \textbf{E}^{\textrm{eff}}\big [|f-P_n|^p\big (u^{\textrm{eff}}_{t_1}(\phi _1), \dots , u^{\textrm{eff}}_{t_k}(\phi _k)\big )\big ]\\&\lesssim \sup _{x_{1:k}\in \mathbb {R}^k} \frac{|f(x_{1:k}) - P_n(x_{1:k})|}{\sum _{i=1}^k e^{c x_i^2} } \sum _{i=1}^k \Big (\mathbb {E}[e^{p c \langle \eta ^\tau , \phi _i\rangle ^2}] + \mathbb {E}[e^{p c \langle \eta , \phi _i\rangle ^2}] \Big )\,. \end{aligned}$$

Now, the sum is finite uniformly in τ and *n* provided $c < \frac{1}{2 p \sigma ^2}$ with $\sigma = \max \{\Vert \phi _i\Vert _{L^2(\mathbb {R}^2)}:i=1,\dots ,n\}$ since $\langle \eta ^\tau , \phi _i\rangle$ and $\langle \eta , \phi _i\rangle$ are centred Gaussian random variables of variance $\Vert \phi _i\Vert _{L_\tau ^2(\mathbb {R}^2)} (\le \Vert \phi _i\Vert _{L^2(\mathbb {R}^2)})$ and $\Vert \phi _i\Vert _{L^2(\mathbb {R}^2)}$, and the prefactor converges to 0 as $n\rightarrow \infty$ from which (1.16) follows at once. □

## Data Availability

No datasets were generated or analysed during the current study.

## Appendix A Regularity, approximation, and Hermite coefficients decay

The goal of this appendix is to provide some insight over the relation between the regularity properties of a function $F:\mathbb {R}\rightarrow \mathbb {R}$ and its coefficients in the Hermite polynomials expansion, so to have a better understanding of Assumption 1.1. What follows is probably classical but since we could not find any good reference, we present it here.

Recall that $\pi _1$ denotes the law of a real valued centred Gaussian random variable with unit variance, and $L^2(\pi _1)$ is the space of square-integrable functions with respect to $\pi _1$. For $m\in \mathbb {N}$, denote by $H_m$ the *m*-th Hermite polynomial which is defined by

A.1 $$\begin{aligned} H_m(x){\mathop {=}\limits ^{\tiny \text {def}}}\frac{(-1)^m}{m!} e^{\frac{x^2}{2}}\frac{\textrm{d}^m}{\textrm{d}x^m}\big (e^{-\frac{x^2}{2}}\big )\,,\qquad x\in \mathbb {R}\,. \end{aligned}$$

The family $(\sqrt{m!} \, H_m)_{m \ge 0}$ forms an orthonormal basis of $L^2(\pi _1)$, so that any $F\in L^2(\pi _1)$ can be written in terms of the Hermite coefficients $(c_m(F))_{m\ge 0}$ as

A.2 $$\begin{aligned} F = \sum _{m \ge 0} c_m(F) \, H_m \, , \qquad c_m(F) {\mathop {=}\limits ^{\tiny \text {def}}}m! \int _{\mathbb {R}} F(x) \, H_m(x) \, \frac{e^{-x^2/2}}{\sqrt{2 \pi }} \, \textrm{d}x \, , \end{aligned}$$

and its $L^2(\pi _1)$-norm satisfies

A.3 $$\begin{aligned} \Vert F\Vert _{L^2(\pi _1)}^2 = \sum _{m \ge 0} \frac{c_m(F)^2}{m!} \, . \end{aligned}$$

For convenience, let us introduce the *normalised* Hermite coeffients of *F*, i.e. the coefficients that correspond to the coordinates of *F* in the orthonormal basis $(\sqrt{m!} \, H_m)_{m \ge 0}$, as

A.4 $$\begin{aligned} \hat{c}_m(F) {\mathop {=}\limits ^{\tiny \text {def}}}\frac{c_m(F)}{\sqrt{m!}} = \langle F, \sqrt{m!}H_m\rangle _{L^2(\pi _1)}\,. \end{aligned}$$

At first, we will establish the relation between the decay in *m* of $\hat{c}_m(F)$ and the $L^2(\pi _1)$-norms of the derivatives of *F*. The following lemma provides an upper bound of the former in terms of the latter.

### Lemma A.1

Let $F\in \mathcal{C}^k(\mathbb {R})$ be such that $F^{(\ell )}\in L^2(\pi _1)$ for all $\ell \le k$. Then, for every $\ell \le k$ and $m\ge \ell$, we have

A.5 $$\begin{aligned} |\hat{c}_m(F)|\le \frac{\Vert F^{(\ell )}\Vert _{L^2(\pi _1)}}{\sqrt{(m-\ell +1) \dots m}}\,. \end{aligned}$$

### Proof

The result is a direct consequence of basic properties of Hermite polynomials. In particular, the key identity is [46, Eq. (1.2)] which states that for every n≥0, $H_{n+1}' \equiv H_{n}$. This, together with a density argument, implies that for any $\ell \le k$,

A.6 $$\begin{aligned} \hat{c}_m(F^{(\ell )}) = \sqrt{(m+1) \dots (m+\ell ) } \, \hat{c}_{m+\ell }(F)\,, \end{aligned}$$

and thus, by (A.3) we get

A.7 $$\begin{aligned} \Vert F^{(\ell )}\Vert _{L^2(\pi _1)}^2 = \sum _{m \ge \ell } (m-\ell +1) \dots m \, \hat{c}_m(F)^2 \end{aligned}$$

which, since the l.h.s. is finite by assumption, immediately gives (A.5). □

### Remark A.2

Compare (A.6) to the classical analogue for the Fourier transform, say for $F \in \mathcal {S}(\mathbb {R})$,

$$\begin{aligned} \mathcal {F}(F^{(\ell )}) \, (m) = (\iota m)^\ell \ \mathcal {F}(F) \, (m) \, . \end{aligned}$$

Here the square root of the ascending ℓ-power of *m* has been replaced by $(\iota m)^\ell$. This suggests that, as for the Fourier transform, the regularity of *F* is tightly connected to the decay of $(c_m(F))_{m \ge 0}$.

The next proposition addresses the main question of the appendix as it states a necessary and sufficient condition for the normalised Hermite coefficients of a function to decay faster than any polynomial.

### Proposition A.3

Let $F \in L^2(\pi _1)$. Then, the sequence $(\hat{c}_m(F))_{m \ge 0}$ decays faster than any inverse power of *m* if and only if the derivatives of every order of *F* belong to $L^2(\pi _1)$.

### Remark A.4

In view of the analogy with the Fourier transform as explained above, this is the exact analogue of the classical fact that a (say periodic, to avoid integrability issues) function is smooth if and only if its Fourier transform decays faster than any inverse powers.

### Proof

“⟸” is a straightforward consequence of Lemma A.1.

“⟹” Note that (A.7) gives that for any fixed δ>0 and every k≥0, we have

A.8 $$\begin{aligned} \Vert F^{(k)}\Vert _{L^2(\pi _1)}&\le \Big (\sum _{m \ge k} \frac{1}{1 + m^{1+2\delta }}\Big )^{1/2} \sup _{m \ge k} (1 + m^{k/2+1/2+\delta }) \, |\hat{c}_m(F)|\nonumber \\&\lesssim _\delta \sup _{m \ge k} (1+m^{k/2+1/2+\delta }) \, |\hat{c}_m(F)| \, , \end{aligned}$$

from which the implication, and thus the statement, follow at once. Strictly speaking, to apply (A.7) we would need to a priori know that the derivatives of *F* belong to $L^2(\pi _1)$; to avoid this difficulty, one can apply (A.7) to the Hermite expansion $(\sum _{m=0}^N c_m(F) H_m)_{N \ge 0}$ to show that all its derivatives are Cauchy in $L^2(\pi _1)$: it is then easy to see that their $L^2(\pi _1)$-limit must coincide with the derivatives of *F* which concludes the proof. □

Let us now see how Assumption 1.1 fits into the previous statement. A first immediate result is that the normalised Hermite coefficients of *F* decay faster than any inverse power, but we can obtain a more quantitative control on this fast decay.

### Proposition A.5

Let $F:\mathbb {R}\rightarrow \mathbb {R}$ be such that Assumption 1.1 holds. Then, there exists a constant C>0 such that for every $n\in \mathbb {N}$

A.9 $$\begin{aligned} \Vert F^{(n)}\Vert _{L^2(\pi _1)}\le C\,\exp (\exp (o(n)))\,. \end{aligned}$$

As a consequence, the normalised Hermite coefficients of *F*, $(\hat{c}_m(F))_{m\ge 0}$ in (A.4), decay faster than any inverse power of *m*, and, for every γ>0 there exists a constant C=C(γ) such that for every k≥0 it holds

A.10 $$\begin{aligned} \sum _{m \ge 1} m^k \, |\hat{c}_m(F)| \le C \exp (\exp (\gamma k)) \, . \end{aligned}$$

### Proof

Note that if *G* is an arbitrary continuous function on $\mathbb {R}$, then we can bound (using that κ<1/4)

A.11 $$\begin{aligned} \Vert G\Vert _{L^2(\pi _1)} \le \Big (\int e^{-(1/2 - 2 \kappa ) \, x^2} \, \textrm{d}x\Big )^{1/2} \sup _{x \in \mathbb {R}} |G(x)| e^{-\kappa x^2} \lesssim _\kappa \sup _{x \in \mathbb {R}} |G(x)| e^{-\kappa x^2} \, . \end{aligned}$$

Therefore, we immediately deduce (A.9) from Assumption 1.1. Then, Proposition A.3 implies the fast decay of the normalised Hermite coefficients of *F*.

At last, we turn to (A.10). Using Cauchy–Schwartz, the trivial bound $m^k \le (2k)^k + 2^k \, m \dots (m-k+1)$, and the identity (A.6), we obtain

$$\begin{aligned}&\sum _{m \ge 1} m^k \, |\hat{c}_m(F)| \lesssim \Big (\sum _{m \ge 1} m^{k+2} \, \hat{c}_m(F)^2\Big )^{1/2}\\&\lesssim \Big ((2k+4)^{k+2} \sum _{m \ge 1} \hat{c}_m(F)^2 + 2^{k+2} \sum _{m \ge 1} m \dots (m - k - 1) \, \hat{c}_m(F)^2\Big )^{1/2} \,\\&=\Big ((2k+4)^{k+2} \Vert F\Vert _{L^2(\pi _1)}^2 + 2^{k+2} \Vert F^{(k+2)}\Vert _{L^2(\pi _1)}^2\big )^{1/2} \, ,\\&\lesssim _\gamma \exp (\exp (\gamma k))\, , \end{aligned}$$

where we applied (A.9) thanks to which γ>0 can be chosen arbitrary small. □

In the next lemma, we show that functions for which Assumption 1.1 holds can be approximated arbitrarily well by suitably chosen polynomials. The statement is a bit more general as in Section 3.1 our assumption on *F* can be relaxed.

### Lemma A.6

Let $\kappa \in (0,1/4)$, $k\in \mathbb {N}\cup \{+\infty \}$ and $F\in \mathcal{C}^k(\mathbb {R})$ be such that, for every $\ell \le k$, $\sup _x |F^{(\ell )}(x)| e^{-\kappa x^2}<\infty$ and $F^{(\ell )}(x)=o(C e^{\kappa x^2})$ for |*x*| large, where C=C(ℓ)>0 is a positive constant. Then, there exists a sequence of polynomials $(P_M)_{M \ge 1}$ such that for every $\ell \le k$,

A.12 $$\begin{aligned} \lim _{M\rightarrow \infty }\sup _{x \in \mathbb {R}} |F^{(\ell )}(x) - P^{(\ell )}_M(x)| e^{- \kappa x^2} = 0 \, . \end{aligned}$$

### Remark A.7

Upon choosing k=1 in the above statement, its assumptions on *F* coincide with (3.3), while for k=∞ we are in the setting of Assumption 1.1.

### Proof

We first claim that the statement holds provided it does for *F* smooth and compactly supported. Let $(\vartheta _N)_{N \ge 1}$ be a sequence of mollifiers, and $(\chi _N)_{N \ge 1}$ a sequence of smooth cut-off functions that converges locally to 1 and have uniformly bounded $\mathcal {C}^k(\mathbb {R})$-norm. Then, for N≥1, define $F_N = \vartheta _N * [\chi _N F]$. Since $F\in \mathcal {C}^k$ and, for every $\ell \le k$, $F^{(\ell )}(x)=o(C e^{\kappa x^2})$, it is easy to check that

$$\begin{aligned} \sup _{x \in \mathbb {R}} |F^{(\ell )}(x) - F_N^{(\ell )}(x)| e^{ -\kappa x^2} \xrightarrow [N \rightarrow \infty ]{} 0 \, , \end{aligned}$$

from which the claim follows.

We now consider *F* smooth and compactly supported. Recall that for any σ>0, the family $(H_m^\sigma )_{m\ge 0}$, where $H_m^\sigma (\cdot ){\mathop {=}\limits ^{\tiny \text {def}}}\sqrt{m!} H_m(\cdot /\sigma )$ and $H_m$ is the *m*-th Hermite polynomial on $\mathbb {R}$, forms an orthonormal basis of $L^2(\pi _\sigma )$, for $\pi _\sigma$ the law of a real-valued centred Gaussian random variable with variance $\sigma ^2$. Denoting by $P_M^\sigma (F)$ the polynomial obtained by projecting *F* onto the space generated by $(H_m^\sigma )_{m\le M}$, i.e.

A.13 $$\begin{aligned} P^\sigma _M(F) = \sum _{m=0}^M \langle F, H_m^\sigma \rangle _{L^2(\pi _\sigma )} H_m^\sigma \end{aligned}$$

we have $\Vert F-P_M^\sigma (F)\Vert _{L^2(\pi _\sigma )}\rightarrow 0$ as $M\rightarrow \infty$. Applying [46, Eq.’s (1.2), (1.3)] and the scaling relation above, it is not hard to see that, for any k≥1, $[P^\sigma _M(F)]^{(\ell )} = P^\sigma _{M-k}(F^{(\ell )})$, so that the previous argument shows that for every k≥1, $\Vert F^{(\ell )}-P_M^\sigma (F)^{(\ell )}\Vert _{L^2(\pi _\sigma )}\rightarrow 0$ as $M\rightarrow \infty$. As a consequence, (A.12) follows provided we are able to bound the (semi-)norm at the l.h.s. by the $L^2(\pi _\sigma )$-norm, which is indeed possible upon choosing σ appropriately. In formulas, we claim that for a function *G* on $\mathbb {R}$ such that $G,G'\in L^2(\pi _{\sigma })$, we have

A.14 $$\begin{aligned} \sup _{x\in \mathbb {R}}|G(x)| e^{-\frac{x^2}{2\sigma ^2}}\lesssim \Vert G\Vert _{L^2(\pi _{\sigma })}+\Vert G'\Vert _{L^2(\pi _{\sigma })} \end{aligned}$$

from which we deduce (A.12) by taking $\sigma ^2=\frac{1}{2\kappa }>1$ and $G=F^{(\ell )}-P_M^{1/\sqrt{2\kappa }}(F)^{(\ell )}$.

It remains to prove (A.14). By Cauchy-Schwartz inequality, we easily see that, for any $x,y\in \mathbb {R}$, we have

$$\begin{aligned} |G(y)-G(x)|&\le \int _{x\wedge y}^{x\vee y}|G'(z)|\textrm{d}z\lesssim \Vert G'\Vert _{L^2(\pi _\sigma )}\Big (\int _{x\wedge y}^{x\vee y}e^{\frac{z^2}{2\sigma ^2}}\textrm{d}z\Big )^{\frac{1}{2}}\\&\lesssim \Vert G'\Vert _{L^2(\pi _\sigma )} \sqrt{|x-y|}\Big (e^{\frac{x^2}{2\sigma ^2}}+e^{\frac{y^2}{2\sigma ^2}}\Big )^{\frac{1}{2}}\lesssim \Vert G'\Vert _{L^2(\pi _\sigma )} \Big (e^{\frac{x^2}{3\sigma ^2}}+e^{\frac{y^2}{3\sigma ^2}}\Big )\,. \end{aligned}$$

and thus

$$\begin{aligned} |G(x)|&\le \left| G(x) - \int _{\mathbb {R}} G(y) \frac{1}{\sqrt{2\pi }\sigma } e^{-\frac{y^2}{2\sigma ^2}} \, \textrm{d}y \right| + \int _{\mathbb {R}} |G(y)| \frac{1}{\sqrt{2\pi }\sigma } e^{-\frac{y^2}{2\sigma ^2}} \, \textrm{d}y \\&\le \int _{\mathbb {R}} |G(x) - G(y)| \frac{1}{\sqrt{2\pi }\sigma } e^{-\frac{y^2}{2\sigma ^2}} \, \textrm{d}y + \Vert G\Vert _{L^2(\pi _\sigma )} \\&\lesssim \Vert G'\Vert _{L^2(\pi _\sigma )} e^{\frac{x^2}{2\sigma ^2}} + \Vert G\Vert _{L^2(\pi _\sigma )} \end{aligned}$$

from which (A.14) follows at once.

We conclude this section with a few concrete examples of functions that satisfy or not the Assumption 1.1.

### Lemma A.8

Let $F :\mathbb {R}\rightarrow \mathbb {R}$ admit a complex analytic extension on the strip $\{z :|\Im (z)| < \delta \}$ for some δ>0, such that the sup-norm of *F* on the rectangle $Q_{M,\delta }:= [-M, M] \times [-\delta , \delta ]$ is dominated by $\exp (\kappa M^2)$ for some $\kappa \in (0,1/4)$. Then, for any $\kappa ' \in (\kappa , 1/4)$, there exists a constant $C \in (0, \infty )$ such that for every n≥0

A.15 $$\begin{aligned} |F^{(n)}(x)| \le C \, \delta ^{-n} \, n! \, \exp (\kappa x^2) \, . \end{aligned}$$

In particular, any such *F* satisfies Assumption 1.1.

### Corollary A.9

The functions $F(x) = \sqrt{a+x^2}$ for a>0 and $G(x) = \Re \exp (\omega x)$ for $\omega \in \mathbb {C}$ satisfy Assumption 1.1.

### Remark A.10

Note that the growth of the derivates in (A.15) is much smaller than what is required by Assumption 1.1. Indeed, it can be shown that it implies the existence of constants $c, C \in (0, \infty )$ such that for every m≥0

A.16 $$\begin{aligned} |\hat{c}_m(F)| \le C \exp (- c \sqrt{m}) \, . \end{aligned}$$

### Proof of Lemma A.8

By the Cauchy integral formula we deduce

$$\begin{aligned} \frac{|F^{(n)}(x)|}{n!} \le \delta ^{-n} \sup _{z \in D(x, \delta )} |F(z)| \lesssim \delta ^{-n} \exp (\kappa \, (x+\delta )^2) \lesssim \delta ^{-n} \exp (\kappa ' x^2) \, , \end{aligned}$$

which concludes. □

Now, let us give an example of a function that does not satisfy Assumption 1.1. Of course, any function that is not smooth trivially fails, such as F(x)=|x|. Let us comment that for the latter, an explicit computations involving integrations by parts shows that

$$\begin{aligned} c_m(F) \sim (-1)^{\frac{m-2}{2}} \frac{2^{3/4}}{\pi ^{3/4}} \frac{1}{m^{5/4}} \, , \qquad \text {for { m} even, as } m\rightarrow \infty \text {,} \end{aligned}$$

which is consistent with Proposition A.3.

Let us now give an example of a smooth function that does not satisfy Assumption 1.1. Let *F* be a smooth function which is flat at every order at 0, such that for $x \in [-\delta , \delta ]$ (for some $\delta \in (0,e^{-1})$) we have

$$\begin{aligned} F(x) = |x|^{(\gamma + o(1)) \log \log (1/x)} \, , \end{aligned}$$

for some γ>0. Then, we claim that

$$\begin{aligned} \sup _{x \in [-\delta ,\delta ]} |F^{(n)}(x)| \ge \exp (\exp ((\gamma ^{-1} + o(1)) \, n)) \,, \end{aligned}$$

so that (1.9) fails. First, the Taylor-Lagrange inequality (relative to the trivial Taylor expansion of *F* at 0) gives for every $x \in (0, \delta )$

$$\begin{aligned} F(x) \le \Vert F\Vert _{\infty , [0, \delta ]} \frac{x^n}{n!} \, , \end{aligned}$$

so that we can lower bound (using the change of variable $\ell = \log (1/x)$)

$$\begin{aligned} \frac{\Vert F^{(n)}\Vert _{\infty , [0, \delta ]}}{n!} \ge \sup _{x \in (0, \delta )} \frac{F(x)}{x^n} = \sup _{\ell \in (\log (1/\delta ), \infty )} \exp (- (\gamma + o(1)) \log (\ell ) \ell + n \ell ) \, . \end{aligned}$$

Optimizing over ℓ (the maximizer is $\ell _* = \exp (n/(\gamma + o(1)) - 1)$, provided *n* is sufficiently large so that $\ell _* \ge \log (1/\delta )$), we deduce

$$\begin{aligned} \Vert F^{(n)}\Vert _{\infty , [0, \delta ]} \ge \exp ((\gamma + o(1)) \exp (n/(\gamma + o(1)) - 1)) = \exp (\exp ((\gamma ^{-1} + o(1)) \, n)) \, . \end{aligned}$$

## Appendix B Some technical results

In this section, we collect a few technical results which are needed in the paper. The first ensures that for every τ>0 fixed, the operators $\mathcal {L}_0^{\mathfrak {e}_1}$, $\mathcal {L}_0^{\mathfrak {e}_2}$, $\mathcal {S}^\tau$ and  are continuous from $\textbf{T}^\tau$ equipped with its natural Fréchet metric to  and derives a quantitative (but τ-dependent) estimate on the latter.

### Lemma B.1

Let τ>0 be fixed. The operators $\mathcal {L}_0^{\mathfrak {e}_1}$, $\mathcal {L}_0^{\mathfrak {e}_2}$, $\mathcal {S}^\tau$ and , for m≥1 and $a \in \{-m+1,-m+3...,m-1\}$, respectively given in (3.28), (3.29), (3.30) and (3.31) are continuous on $\textbf{T}^\tau$. Furthermore, for every n≥1, there exists a constant C=C(τ,n)>0 such that for every *m* and every $\psi \in \textbf{T}^\tau _n$ we have

B.1 $$\begin{aligned} \Vert \sqrt{m!} \,\mathcal {A}^{\tau , m} \psi \Vert _\tau \le C \, m^{n/2} \, \Vert \psi \Vert _\tau \, , \end{aligned}$$

### Proof

The continuity of the operators $\mathcal {L}_0^{\mathfrak {e}_1}$, $\mathcal {L}_0^{\mathfrak {e}_2}$ and $\mathcal {S}^\tau$ from $\textbf{T}^\tau$ to  is straightforward. For , it follows from the more quantitative estimate (B.1). The case m=1 is trivial, so let us look at m>1 (notice that the bound for m=2 was given in [15, Lemma B.2]). Let $\psi \in \textbf{T}^\tau _n$ and recall the definition of $\mathcal {A}_j^{\tau , m} \psi$ in (3.31). Since $\hat{\psi }$ is symmetric with respect to permutation of its variables, we see that

$$\begin{aligned} \Vert \mathcal {A}_j^{\tau , m}&\psi \Vert _\tau ^2 \lesssim \tau ^2\nu _\tau \frac{n!^2}{(n+a)! (j+1)!^2} \left( {\begin{array}{c}n+a\\ m-j\end{array}}\right) ^2\\&\times \tau ^{-m} \nu _\tau ^{\frac{m}{2}}\int \Xi _{n+a}^\tau (\textrm{d}p_{1:n+a}) \, \Big |\int _{r_{[1:j+1]} = p_{[1:m-j]}} \Xi _{j+1}^\tau (\textrm{d}r_{1:j+1}) \, \psi (r_{1:j+1}, p_{m-j+1:n+a}) \Big |^2\\&\lesssim \tau ^2\nu _\tau \frac{n!^2}{(n+a)! (j+1)!^2} \left( {\begin{array}{c}n+a\\ m-j\end{array}}\right) ^2\Vert \psi \Vert _\tau ^2 \end{aligned}$$

where in the last step we applied Cauchy-Schwarz to the integral with respect to the measure $\Xi _{j+1}^\tau$, used that, by (2.5), $\Xi _{n+a}^\tau (\textrm{d}p_{1:n+a})=\Xi _{m-j}^\tau (\textrm{d}p_{1:m-j})\Xi _{n+a-m+j-1}^\tau (\textrm{d}p_{m-j+1:n+a})$ and that, by the assumptions on ρ and the definition of $\rho _\tau$ and $\Theta ^\tau$ in (2.4), the total mass of $\Theta ^\tau$ is proportional to $\tau \nu _\tau ^{-1/2}$. Therefore, it remains to estimate the prefactor of the -norm of τ, which satisfies

$$\begin{aligned} \frac{n!}{(n+a)! (j+1)!^2} \left( {\begin{array}{c}n+a\\ m-j\end{array}}\right) ^2=\frac{1}{(m+1)!} \left( {\begin{array}{c}n\\ j+1\end{array}}\right) \left( {\begin{array}{c}m+1\\ j+1\end{array}}\right) \left( {\begin{array}{c}n+m-2j-1\\ n-j-1\end{array}}\right) \lesssim _n \frac{m^n}{m!} \end{aligned}$$

the last step being a consequence of $$\left( {\begin{array}{c}M\\ N\end{array}}\right) \le M^N$$. Putting the two together, (B.1) follows at once. □

Next, we want to check that if *F* is a Hermite polynomial of given degree then the operator $A^\tau$ given in (3.25) coincides with $\mathcal {A}^{\tau }$ in Lemma 3.8. The proof is rather standard and, for *F* quadratic, can be found in, e.g., [30], but since here the computations are more involved and the case of Hermite polynomials of degree different than 2 has never been treated, we provide below full details.

### Lemma B.2

For any τ>0, $m\in \mathbb {N}$, $n\in \mathbb {N}$ and $\phi =I_n^\tau (h^{\otimes n}) = n! H_n( \eta ^\tau (h))$ with *h* such that $\hat{h}\in \mathcal {C}_c^\infty (\mathbb {R}^2)$ and $\Vert h\Vert _{L^2_\tau (\mathbb {R}^2)} = 1$, we have

where $A^\tau _{H_m}=A^\tau$ is defined in (3.25) with *F* given by the *m*-th Hermite polynomial $H_m$, and  in (3.32). Further, on the set of ϕ’s as above,  is skew-symmetric, i.e. .

### Proof

Let ϕ be as in the statement. Recall that by [46, Eq. (1.2)], for any Hermite polynomial $H_n$ we have $H_n'\equiv H_{n-1}$. Hence,

B.2 $$\begin{aligned} A^\tau \phi (\eta ^\tau )&= \langle \mathcal {N}_{H_m}^\tau (\eta ^\tau ), h \rangle _{L^2(\mathbb {R}^2)} n! H_{n-1}( \eta ^\tau (h)) = \langle \mathcal {N}_{H_m}^\tau (\eta ^\tau ), h \rangle _{L^2(\mathbb {R}^2)} \, n I_{n-1}^\tau (h^{\otimes n-1}) \nonumber \\&=- \tau \nu _\tau ^{1/4} \, \langle H_m(\tau ^{-1/2} \nu _\tau ^{1/4} \eta ^\tau ), \partial _1 (\rho _\tau ^{*2} - 1\!\!1_{m=1} \delta _0) * h \rangle _{L^2(\mathbb {R}^2)} \, n I_{n-1}(h^{\otimes n-1})\,.\nonumber \\ \end{aligned}$$

We want to write the scalar product in terms of the isometry $I_m$. To do so, notice that, for every $x\in \mathbb {R}^2$

$$\begin{aligned} \tau ^{-1/2} \nu _\tau ^{1/4} \eta ^\tau (x) = \eta ^\tau \big (\tau ^{-1/2} \nu _\tau ^{1/4} \delta _x \big )\qquad \text {and}\qquad \Vert \tau ^{-1/2} \nu _\tau ^{1/4} \delta _x\Vert _{L^2_\tau (\mathbb {R}^2)} = 1\,, \end{aligned}$$

and thus

$$\begin{aligned} H_m\big (\tau ^{-1/2} \nu _\tau ^{1/4} \eta ^\tau (x)\big )&= \frac{1}{m!} \, I_m^\tau \big ((\tau ^{-1/2} \nu _\tau ^{1/4} \delta _x)^{\otimes m}\big ) = \frac{\tau ^{-m/2} \nu _\tau ^{m/4}}{m!} \, I_m^\tau \big (\delta _x^{\otimes m}\big ) \, . \end{aligned}$$

We will focus on the case m≥2, as for m=1 the computations are similar and simpler. The definition of $I_m^\tau$ and its linearity ensure that

$$\begin{aligned} \langle&H_m\big (\tau ^{-1/2} \nu _\tau ^{1/4} \eta ^\tau \big ), \partial _1 \rho _\tau ^{*2} * h \rangle _{L^2(\mathbb {R}^2)} = \frac{\tau ^{-m/2} \nu _\tau ^{m/4}}{m!} \, I_m^\tau \Big (\int _{\mathbb {R}^2} \textrm{d}x \, [\partial _1 \rho _\tau ^{*2} * h](x) \, \delta _{x}^{\otimes m}\Big ) \ , \end{aligned}$$

so that the product rule in (2.8) gives

B.3 $$\begin{aligned} A^\tau \phi (\eta ^\tau )=n\frac{\tau ^{1-\frac{m}{2}}\nu _\tau ^{\frac{m+1}{4}}}{m!}\sum _{j=0}^{m\wedge (n-1)} j! \left( {\begin{array}{c}m\\ j\end{array}}\right) \left( {\begin{array}{c}n-1\\ j\end{array}}\right) I_{m+n-1-2j}\big ( \textrm{Sym}(\psi _{m,j})\big ) \end{aligned}$$

where $\psi _{m,j}\in L_\tau ^2(\mathbb {R}^{2(n+m-2j-1)})$ is defined via (2.9), i.e. its Fourier transform is given by

$$\begin{aligned} \mathcal {F}&(\psi _{m,j})(p_{1:m-j}, q_{1:n-1-j})\\&{\mathop {=}\limits ^{\tiny \text {def}}}\int \Xi ^\tau _j(\textrm{d}r_{1:j}) \, \mathcal {F}\Big (\int _{\mathbb {R}^2} \textrm{d}x \, [\partial _1 \rho _\tau ^{*2} * h](x) \, \delta _{x}^{\otimes m}\Big )(p_{1:m-j}, r_{1:j})\mathcal {F}(h^{\otimes n-1})(q_{1:n-1-j}, -r_{1:j})\\&=- \frac{\iota }{(2\pi )^{m-1}}\int \Xi ^\tau _j(\textrm{d}r_{1:j}) \, \Theta ^\tau (p_{[1:m-j]} - r_{[1:j]}) \, \mathfrak {e}_1 \cdot (p_{[1:m-j]} -r_{[1:j]})\\&\qquad \qquad \times \hat{h}(p_{[1:m-j]} - r_{[1:j]}) \, \hat{h}(q_1) ... \hat{h}(q_{n-1-j}) \, \hat{h}(r_1) ... \hat{h}(r_j) \end{aligned}$$

Now, if j=m≤n-1, we extract those $\hat{h}$ depending on $q_i$’s and the remaining integral reads

$$\begin{aligned} \frac{\iota }{(2\pi )^{m-1}}\int \, \Theta ^\tau (- r_{[1:m]})&\, \mathfrak {e}_1 \cdot (-r_{[1:m]})\hat{h}(- r_{[1:m]}) \hat{h}(r_1) ... \hat{h}(r_m) \,\Xi ^\tau _m(\textrm{d}r_{1:m})\\&=\int [\partial _{\mathfrak {e}_1}(\rho _\tau ^{*2}*h)](x)\, (\rho _\tau ^{*2}*h)^m(x)\,\textrm{d}x=0 \end{aligned}$$

the first equality being a consequence of the definition of $\Xi ^\tau _m$ in (2.5) and Fourier inversion formula, and the second from the fact that the integrand is a total derivative. Instead, for $j=0,\dots (m-1)\wedge (n-1)$ we have

$$\begin{aligned} \mathcal {F}&(\psi _{m,j})(p_{1:m-j}, q_{1:n-1-j})\\&=- \frac{\iota }{(2\pi )^{m-1}}\int _{r_{[1:j+1]} = p_{[1:m-j]}} \Xi _{j+1}^\tau (\textrm{d}r_{1:j+1})\,\mathfrak {e}_1 \cdot r_{j+1}\, \hat{\phi }(r_{1:j+1}, q_{1:n-1-j})\\&=- \frac{\iota }{(2\pi )^{m-1}}\frac{\mathfrak {e}_1 \cdot p_{[1:m-j]}}{j+1} \int _{r_{[1:j+1]} = p_{[1:m-j]}} \Xi _{j+1}^\tau (\textrm{d}r_{1:j+1}) \, \hat{\phi }(r_{1:j+1}, q_{1:n-1-j}) \end{aligned}$$

where in the second step we introduced the fictitious variable $r_{j+1}=p_{[1:m-j]} - r_{[1:j]}$ and identified ϕ with its kernel $h^{\otimes n}$ and in the last we symmetrised the integral with respect to the variables $r_{1:j+1}$. At this point, for $j\in \{0,\dots (m-1) \wedge (n-1)\}$ the above computations show that, before symmetrisation, the Fourier transform of the kernel of the *j*-th summand at the r.h.s. of (B.3) equals

$$\begin{aligned} -\iota&\frac{\tau ^{1-\frac{m}{2}} \nu _\tau ^{\frac{1+m}{4}}}{(2\pi )^{m-1}} \frac{n}{j+1}\, j! \left( {\begin{array}{c}m\\ j\end{array}}\right) \left( {\begin{array}{c}n-1\\ j\end{array}}\right) \times \\&\times \mathfrak {e}_1 \cdot p_{[1:m-j]} \int _{r_{[1:j+1]} = p_{[1:m-j]}} \Xi _{j+1}^\tau (\textrm{d}r_{1:j+1}) \, \hat{\phi }(r_{1:j+1}, q_{1:n-1-j}) \end{aligned}$$

which, by setting $a{\mathop {=}\limits ^{\tiny \text {def}}}m-1-2j$, relabelling the variables $q_{1:n-j}=p_{1:n+a\setminus \{1:m-j\}}$ and symmetrising with respect to $p_{1:n+a}$, coincides with the r.h.s. of (3.31). In other words, we proved that

and since, for $F=H_m$,  the result follows.

It remains to prove that  is skew-symmetric. But this is an immediate consequence of the fact that  which in turn can be verified via a direct computation using the expression of these operators in (3.31).

□

## Footnotes

1 In contrast, when $c_2(F) = 0$, we expect the system (1.7) to be *diffusive* at large scales.

2 Even though these works do not directly discuss SPDEs but SDEs, quadratic SPDEs still appear at the level of the so-called *environment seen by the particle*.

3 The second condition is always satisfied up to taking any larger κ.

4 The problem essentially reduces to the fact that we do not know whether $\textbf{T}^\tau$ is a *core* for $\mathcal {L}^\tau$ for τ finite. Indeed, even for fixed τ, the graded sector condition of [41, Section 3.7.4] does not hold due to the growth in the number operator (see e.g. (B.1)).
